# Co-targeting of BAX and BCL-XL proteins broadly overcomes resistance to apoptosis in cancer

**DOI:** 10.1038/s41467-022-28741-7

**Published:** 2022-03-07

**Authors:** Andrea Lopez, Denis E. Reyna, Nadege Gitego, Felix Kopp, Hua Zhou, Miguel A. Miranda-Roman, Lars Ulrik Nordstrøm, Swathi-Rao Narayanagari, Ping Chi, Eduardo Vilar, Aristotelis Tsirigos, Evripidis Gavathiotis

**Affiliations:** 1 Department of Biochemistry, Albert Einstein College of Medicine, Bronx, NY USA; 2 Department of Medicine, Albert Einstein College of Medicine, Bronx, NY USA; 3 Albert Einstein Cancer Center, Albert Einstein College of Medicine, Bronx, NY USA; 4 Department of Pathology, NYU Langone Health and School of Medicine, New York, NY USA; 5 Laura and Isaac Perlmutter Cancer Center, NYU Langone Health and School of Medicine, New York, NY USA; 6 Applied Bioinformatics Laboratories, NYU School of Medicine, New York, NY USA; 7 Human Oncology and Pathogenesis Program, Memorial Sloan Kettering Cancer Center, New York, NY USA; 8 Louis V. Gerstner Jr. Graduate School of Biomedical Sciences, Memorial Sloan Kettering Cancer Center, New York, NY, USA; 9 Department of Cell Biology, Albert Einstein College of Medicine, Bronx, NY USA; 10 Department of Medicine, Memorial Sloan Kettering Cancer Center, New York, NY USA; 11 Department of Medicine, Weill Cornell Medicine, New York, NY USA; 12 Department of GI Medical Oncology, The University of Texas MD Anderson Cancer Center, Houston, TX USA

**Keywords:** Targeted therapies, Apoptosis, Target validation

## Abstract

Deregulation of the BCL-2 family interaction network ensures cancer resistance to apoptosis and is a major challenge to current treatments. Cancer cells commonly evade apoptosis through upregulation of the BCL-2 anti-apoptotic proteins; however, more resistant cancers also downregulate or inactivate pro-apoptotic proteins to suppress apoptosis. Here, we find that apoptosis resistance in a diverse panel of solid and hematological malignancies is mediated by both overexpression of BCL-XL and an unprimed apoptotic state, limiting direct and indirect activation mechanisms of pro-apoptotic BAX. Both survival mechanisms can be overcome by the combination of an orally bioavailable BAX activator, BTSA1.2 with Navitoclax. The combination demonstrates synergistic efficacy in apoptosis-resistant cancer cells, xenografts, and patient-derived tumors while sparing healthy tissues. Additionally, functional assays and genomic markers are identified to predict sensitive tumors to the combination treatment. These findings advance the understanding of apoptosis resistance mechanisms and demonstrate a novel therapeutic strategy for cancer treatment.

## Abstract (web summary)

Deregulation of the BCL-2 family interactions ensures cancer resistance to apoptosis and is a major challenge to current treatments. Here the authors describe a novel therapeutic strategy to overcome two anti-apoptotic mechanisms for cancer therapy.

## Introduction

Deregulated apoptosis is a hallmark of cancer^1^. Cancer cells prevent apoptosis to ensure their survival and growth, and becoming resistant to current treatments^2–5^. The intrinsic or mitochondrial pathway of apoptosis is regulated by the BCL-2 family of proteins that include: the pro-apoptotic or effector proteins (BAX, BAK, and BOK), the anti-apoptotic or survival proteins (e.g., BCL-2, BCL-XL, MCL-1), and the pro-apoptotic BH3-only proteins classified either as activators (e.g., BIM, BID) or sensitizers (e.g., BAD, HRK)^3,6^. Frequently, cancer cells upregulate anti-apoptotic BCL-2 family members to inhibit pro-apoptotic BCL-2 members BAX, BAK, and BH3-only proteins, thus blocking apoptosis^7–9^. Furthermore, more resistant cancers also downregulate or inactivate pro-apoptotic BH3-only proteins, making these tumors more insensitive to current treatments^4,10–15^.

Considering the critical role of anti-apoptotic BCL-2 proteins in apoptosis resistance by cancer cells, selective drugs designed to inhibit anti-apoptotic BCL-2 proteins, termed BH3 mimetics, have been developed. BH3 mimetics, including Venetoclax, Navitoclax, S63845, and AMG176, induce apoptosis primarily by releasing BH3-only proteins (e.g., BIM and BID) from the anti-apoptotic BCL-2 proteins to successively activate BAX and BAK^16–19^. In preclinical and clinical studies, BH3 mimetics showed significant efficacy in tumors when their survival is highly dependent on the targeted anti-apoptotic BCL-2 family protein^20–22^. However, these molecules have shown limited single-agent activity in tumors that upregulate additional non-targeted anti-apoptotic BCL-2 family proteins to ensure survival^16–19,23–27^. Therefore, the full potential of BH3 mimetics to induce tumor apoptosis is yet to be determined by using rational and safe combination treatments to help overcome resistance mechanisms to apoptosis and the identification of predictive biomarkers for precision therapy^20,28,29^.

Pro-apoptotic BAX is the critical effector of mitochondrial apoptosis induced by most BH3 mimetics and chemotherapeutic agents^25,30–32^. Typically, upon pro-apoptotic stimuli, BH3-only proteins use their BH3-domain helix to activate BAX, leading to BAX translocation and oligomerization at the mitochondrial outer membrane (MOM)^33,34^. This causes the MOM permeabilization (MOMP) and release of apoptogens such as cytochrome c and Smac/Diablo that activate the caspase cascade of apoptosis^31^. The elucidation of the BAX trigger site where BH3-domain helix binds to induce BAX activation^33–36^, enabled the discovery of small-molecule BAX activators that engage the trigger site and mimic BH3-only proteins in inducing conformational activation of BAX and apoptosis^37^. Previously, we reported the development of the BAX activator BTSA1 that binds with high affinity and specificity to the BAX trigger site inducing all hallmarks of BAX activation^38^. BTSA1 induced BAX-mediated apoptosis in Acute Myeloid Leukemia (AML) in vitro and in vivo while sparing healthy tissues^38^. While these studies provided the first proof-of-concept for direct BAX activation as an effective treatment strategy in AML; the full therapeutic potential of this emerging strategy to other tumors resistant to apoptosis has not been exploited.

Based on the understanding that cancer cells contain functional BAX and only infrequently BAX is mutated or not expressed^39^, we compared the efficacy of direct and indirect BAX activation in a variety of solid tumors and hematological malignancies. We identified mechanisms of apoptotic resistance mediated by high protein levels of BCL-XL and an unprimed apoptotic state. These survival mechanisms can be overcome by the pharmacological combination of BAX activation and BCL-XL inhibition. Furthermore, we addressed the therapeutic potential of this novel drug combination treatment, in vitro and in vivo, and determined functional assays and biomarkers to predict tumor sensitivity to this treatment. Our findings provide insights into apoptotic resistance mechanisms and investigate the combination of direct BAX activation and BCL-XL inhibition as a novel therapeutic strategy that can broadly overcome apoptotic resistance in cancer.

## Results

### Characterization of a novel BAX activator BTSA1.2

To improve biological activity and in vivo properties of the small molecule BAX activator, BTSA1^38^, we performed further medicinal chemistry optimization. A new analog, BTSA1.2, which has two methyl groups on the thiazole group of BTSA1, was generated to increase van der Waals contacts with the BAX trigger site based on the previously determined binding pose of BTSA1^38^ (Supplementary Fig. 1a and Supplementary Table 1). In addition, the two methyl groups were installed to increase metabolic stability and avoid potential generation of the reactive and toxic metabolite aminothiazole from in vivo metabolism of BTSA1 (Supplementary Table 2). BTSA1.2 has the phenyl attached to the pyrazolone group as BTSA1, which provided significantly increased binding to BAX and apoptotic activity compared to BAM7 and Compound 4 that lack this phenyl group (Supplementary Table 1). BTSA1.2 has the thiazolhydrazone moiety as Compound 4 showed thiazolhydrazone improved binding compared to the ethoxy phenylhydrazone of BAM7 (Supplementary Table 1). Installing a carboxylic acid to the phenylhydrazone of Compound 5 and to the phenylthiazol of Compound 6 provided less active compounds compared to BTSA1.2 and BTSA1 suggesting that hydrophobic groups are better tolerated to these two rings (Supplementary Table 1).

BTSA1.2 demonstrated increased binding to BAX and more potent cellular activity in a set of lymphoma cell lines compared to BTSA1 (Supplementary Fig. 1b, c and Supplementary Table 1). Moreover, a significant increase in the melting temperature of cellular BAX but not BAK using a Cellular Thermal Shift Assay (*CETSA*) assay provided evidence of BTSA1.2 directly engaging with cellular BAX (Supplementary Fig. 1d–g). Pharmacokinetic analysis of BTSA1.2 demonstrated favorable properties by oral administration such as substantial half-life (T_1/2_ ~14 h) in mouse plasma, favorable oral bioavailability (%F ~50%) and significant plasma exposure (AUC ~100 μΜ h) (Supplementary Fig. 2a–c). Comparison of BTSA1.2 and BTSA1 in vivo by oral administration of 200 mg/Kg daily dose also confirmed that BTSA1.2 is better tolerated by oral administration as mice survived 5 daily doses without obvious toxic effects. In contrast, mice on BTSA1 died after three daily doses by kidney failure and on the second day mice showed increased white blood cells and neutrophils levels (Supplementary Fig. 2d, e). Thus, BTSA1.2, a rationalized BTSA1 analog, has improved binding to BAX, cellular cytotoxicity, and is better-tolerated in vivo.

### Direct and indirect BAX activation is regulated by BCL-XL and apoptotic priming in apoptosis resistant solid tumors

**Fig. 1:**
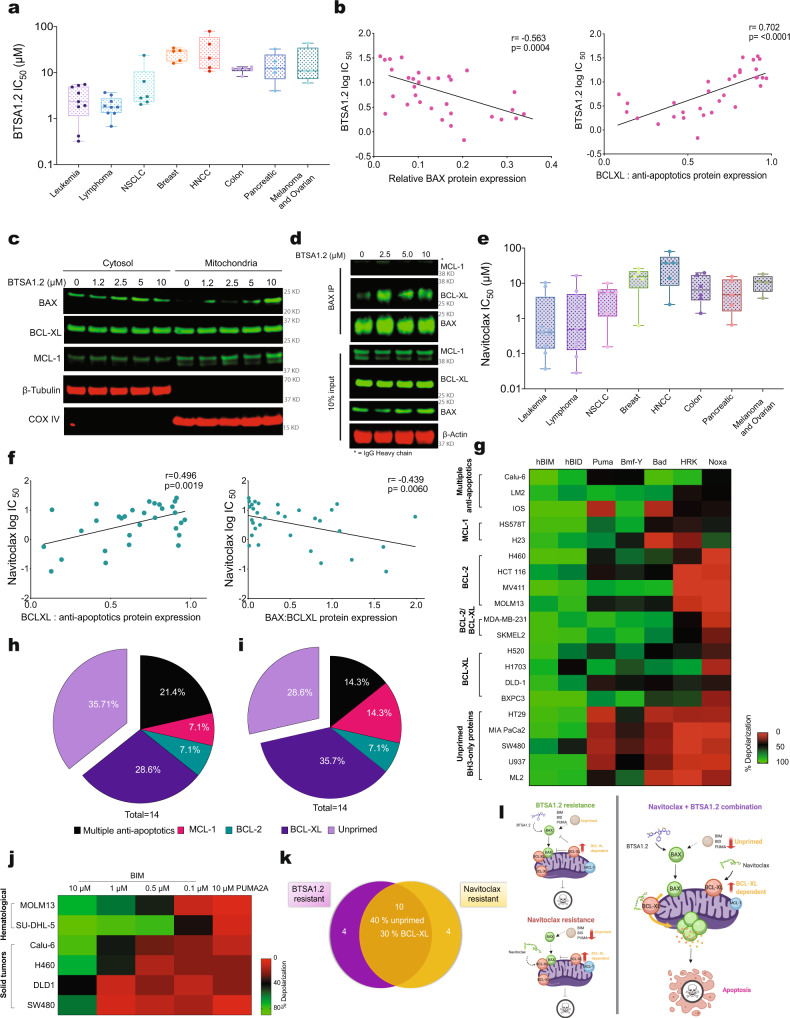
Resistance to direct BAX activation and BCL-XL inhibition is regulated by BCL-XL upregulation and an unprimed state. **a** A diverse collection of cancer cell lines (*n* = 46) treated for 72 hrs with BTSA1.2. Box plot corresponds to the tissue type mean cell viability IC_50_ (μM), cell lines were categorized as sensitive (IC_50_ < 3 μM) or resistant (IC_50_ > 3 μM). The lines within the boxes show the median IC_50_ values, the box denotes the interquartile range (IQR), while the whiskers indicate maxima and minima values. **b** Correlation of sensitivity to BTSA1.2 with BAX and BCL-XL relative protein levels using Pearson-Correlation. Relative protein levels were normalized to β-Actin loading control, *p* value was calculated using two-tailed student *t*-test. **c** BAX translocation upon 4 h treatment with BTSA1.2 in BxPC-3 cells. **d** BAX co-IP upon 4 h treatment with BTSA1.2 in BxPC-3 cells. Data are representative of *n* = 3 independent experiments **e**, A diverse collection of cancer cells (*n* = 46) treated for 72 h with Navitoclax. Box plot corresponds to the tissue type mean cell viability IC_50_ (μM), cell lines were categorized as sensitive (IC_50_ < 1.5 μM) or resistant (IC_50_ > 1.5 μM). The lines within the boxes show the median IC_50_ values, the box denotes the IQR, while the whiskers indicate maxima and minima values **f**, Correlation of sensitivity to Navitoclax with BCL-XL and BAX:BCL-XL relative protein levels using Pearson-Correlation. Relative protein levels were normalized to β-Actin loading control, p value was calculated using two-tailed student *t*-test. **g** Heatmap representation of % mitochondria depolarization of 20 cancer cell lines were classified on different apoptotic blocks based on the BH3 profiling approach. **h**, **i** BH3 profiling predicts apoptotic blocks correlated with resistance to **h**, BTSA1.2 (IC_50_ > 3 μM) and **i**, Navitoclax (IC_50_ > 1.5 μM). **j** Heatmap representation of mitochondria depolarization upon BIM peptide treatment in hematological and solid tumor malignancies. Puma 2 A peptide was used as negative control. **k** Venn diagram comparing cell lines resistant to BTSA1.2 and Navitoclax as single agents. **l** Diagram illustrating the therapeutic strategy of combination treatment with BTSA1.2 and Navitoclax to enhance apoptotic cell death. Data in **g** an **j** are mean of three replicates from *n* = 2 independent experiments. Source data are provided as a Source Data file.

We evaluated the capacity of BTSA1.2 to promote cytotoxicity in a diverse panel of human solid and hematological tumor cell lines (*n* = 46). This panel includes non-small cell lung cancer (NSCLC), breast, head and neck, colorectal, pancreatic, melanoma, ovarian, leukemia, and lymphoma cancer cell lines which contain common genomic alterations in cancer (including mutations on TP53, RAS, BRAF, and/or PIK3CA) (Supplementary Table 3). Similar to other BH3-mimetics as Venetoclax and S63845^17,19,27^, BTSA1.2 treatment showed significantly better cytotoxicity in leukemia and lymphoma cell lines (mean IC_50_ < 3 μM) than in most solid tumor cell lines (mean IC_50_ > 10 μM) (Fig. 1a, and Supplementary Fig. 3a, b).

We hypothesized that anti-apoptotic BCL-2 proteins could promote resistance to the BAX activator treatment. To address this, quantitative protein expression profiling of key BCL-2 family proteins was conducted in the diverse panel of cancer cell lines. We found a strong correlation of BTSA1.2 cytotoxicity with BAX expression levels (Fig. 1b). Interestingly, among key BCL-2 family proteins only anti-apoptotic BCL-XL levels correlated with the less potency of BTSA1.2; suggesting that BCL-XL is a key protein promoting resistance to the BAX activator treatment (Fig. 1b and Supplementary Fig. 4a–d). To assess the role of BCL-XL to the BTSA1.2 treatment, we examined whether BAX is activated in solid tumors cell lines BxPC-3 and SW480, which were less sensitive to BTSA1.2 in our panel of cell lines. Cytosolic BAX translocation to the mitochondria was achieved at 10 μΜ BTSA1.2 in BxPC-3 cells and in SW480 cells at 4hrs (Fig. 1c and Supplementary Fig. 5a, b). However, a lower concentration of BTSA1.2 (2.5-5 μΜ) also promoted cytosolic BAX translocation to the mitochondria in BxPC-3 cells at later time point of 18 hrs (Supplementary Fig. 5c). Interestingly, co-immunoprecipitation of BAX in SW480 and BxPC-3 cells showed BAX:BCL-XL complexes were formed without BTSA1.2 treatment and BAX:BCL-XL complexes but not BAX:MCL-1 complexes increased upon BTSA1.2 treatment (Fig. 1d and Supplementary Fig. 5d, e). Moreover, complexes of BAX:BCL-XL were detected in several solid tumor cell lines without BTSA1.2 treatment (Supplementary Fig. 5f, g). Taken together this data suggested that BTSA1.2 is capable to induce BAX activation and translocation but the onset of BAX-mediated apoptosis may be hindered by the interaction of BCL-XL with BAX.

Since BCL-XL was suggested as a factor of resistance to direct BAX activation in our panel of cancer cell lines, we examined whether Navitoclax, a clinical BCL-XL/BCL-2 inhibitor, would be able to promote cytotoxicity against the same panel of cancer cell lines. Of note, BCL-2 inhibition by Navitoclax should not account for the decrease of cell viability as only a small portion of solid tumors cell lines had detectable levels of BCL-2 protein (Supplementary Fig. 4a). Interestingly, several solid tumor cancer cell lines that were found to be resistant to BTSA1.2 treatment, were also less responsive to BCL-XL inhibition by Navitoclax, which was not sufficient to decrease cell viability in several of these cell lines (mean IC_50_ > 10 μM) (Fig. 1e and Supplementary Fig. 6a, b). Similar to previous reports, analysis of key BCL-2 family proteins expression showed that higher MCL-1 and BCL-XL levels correlated with resistance to Navitoclax treatment^40,41^. On the other hand, BAX:BCL-XL ratio correlated with Navitoclax sensitivity, suggesting that cells with higher expression of BAX will, in general, be more sensitive to BCL-XL inhibition (Fig. 1f and Supplementary Fig. 6c). Collectively these data suggested that a BCL-XL inhibitor as a single agent is not adequate to promote apoptosis and highlight the close relationship of BCL-XL with BAX activation in these solid tumor cell lines.

Next, we conducted the BH3-profiling methodology^42,43^, an alternative approach to identify survival mechanisms adopted by cancer cell lines to avoid apoptosis (Fig. 1g and Supplementary Fig. 7). BH3-profiling analysis demonstrated that cell lines rely on (1) being unprimed to apoptosis; depolarization did not occur upon treatment with sensitizer BH3 peptides e.g., BAD, HRK, NOXA, but only occurred upon addition of activator BH3 peptides e.g., BIM, BID, PUMA consistent with BAX/BAK not being activated at basal conditions, or (2) dependent on one or more anti-apoptotic BCL-2 proteins for survival; depolarization occurred upon adding a specific sensitizer BH3-only peptide and not only upon addition of an activator BH3 peptide. Interestingly, most BTSA1.2 resistant and Navitoclax resistant cell lines were categorized into two major anti-apoptotic survival mechanisms: anti-apoptotic BCL-XL dependent or unprimed to apoptosis (Fig. 1h, i). Indeed, the BH3-profiling data do not support that the majority of solid tumor cell lines are BCL-XL dependent for their survival since similar depolarization from HRK and BAD peptides is observed only with a few cell lines. The BH3-profiling data do not exclude the case that activated BAX by BTSA1.2 or BIM BH3 peptide can be still controlled by the availability of BCL-XL to neutralize activated BAX. Therefore, since some anti-apoptotic BCL-XL-dependent cell lines were resistant to inhibition of BCL-XL by Navitoclax (Supplementary Fig. 6b and Fig. 1g) this suggested to us that another pro-survival mechanism, as unprimed to apoptosis, could also play a role in apoptosis resistance in these cell lines. Indeed, by examining the apoptotic priming status of solid tumor and hematological malignancies with activator BIM BH3 peptide, we determined that solid tumors classified as BCL-XL dependent were less primed than hematological malignancies (Fig. 1j). Furthermore, taking into account cell lines that were resistant to both single treatments of BTSA1.2 and Navitoclax, we found that most of these cell lines where unprimed for apoptosis (Fig. 1k).

Collectively, these data indicated that targeting one survival mechanism is not sufficient to induce potent apoptosis in resistant solid tumor cell lines. Therefore, we rationalized that a dual treatment of BTSA1.2 and Navitoclax could overcome both survival mechanisms by enhancing apoptotic priming with direct BAX activation and inhibiting anti-apoptotic blockade with BCL-XL inhibition to promote apoptosis (Fig. 1l).

### BTSA1.2 and Navitoclax synergize to induce apoptosis in resistant tumor cell lines

**Fig. 2:**
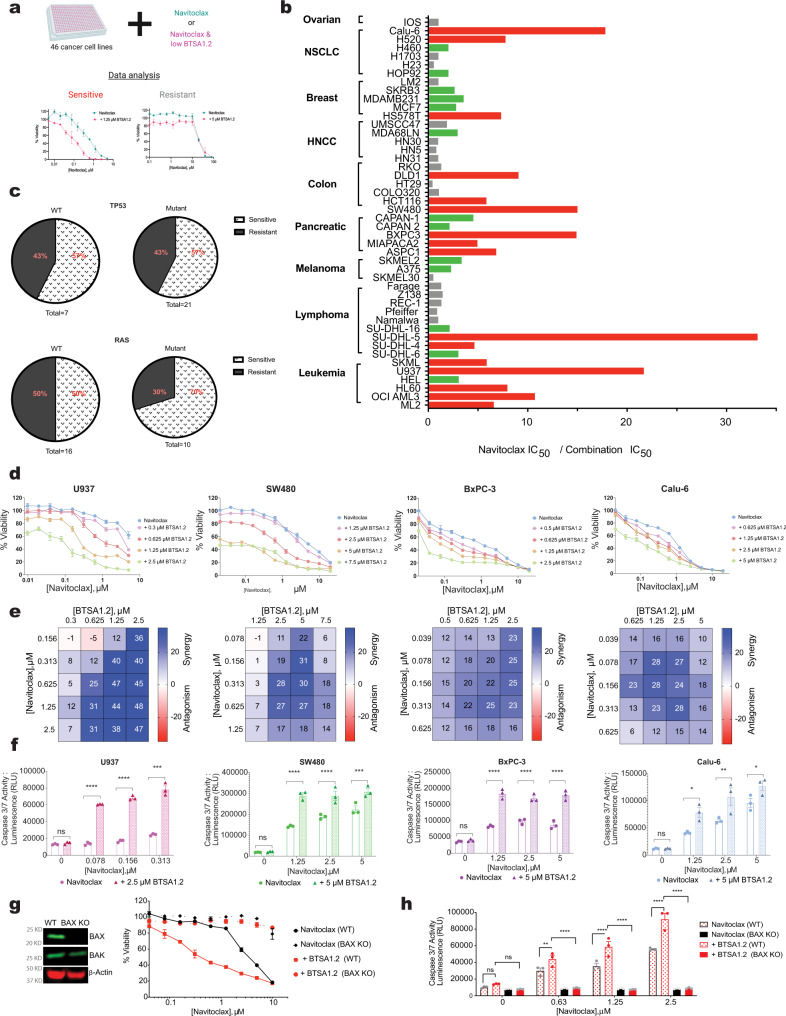
BTSA1.2 and Navitoclax synergize to inhibit cell viability and induce apoptosis in various tumor cell lines. **a** Schematic of Navitoclax and BTSA1.2 combination screening in a diverse cancer cell lines panel (*n* = 46). **b** Bar graph plot of the cell viability IC_50_ (μM) fold change of cancer cells treated for 72 hrs with Navitoclax in combination with a constant sensitizing concentration of BTSA1.2 (loss of cell viability 0–20%). Red bar graphs correspond to IC_50_ fold change > 5x; green bar graphs correspond to IC_50_ fold change 2-4x; and gray bar graphs correspond to IC_50_ fold change <2x. **c** Mutation status of TP53 and RAS in cancer cell lines classified as sensitive or resistant to the combination. **d** Dose-response curves of Navitoclax in the presence of various doses of BTSA1.2 in a panel of cancer cell lines resistant to single agents (Leukemia = U937, Colon=SW480, Pancreatic=BxPC-3, NSCLC = Calu-6). Data are mean ± SD of three technical replicates from n = 3 independent experiments. **e** Bliss synergy score heat map from combinatorial treatment of BTSA1.2 and Navitoclax in different cancer tissue types in **b**. Data represent mean from n = 3 independent experiments. **f** Caspase 3/7 activity assay in diverse cancer cell lines treated with BTSA1.2 and Navitoclax alone or in combination measured at 8 hrs. Data are mean ± SD of three technical replicates from *n* = 3 independent experiments. Statistics were obtained using two-way ANOVA: *p < 0.05; **p < 0.01; ***p < 0.001; ****p < 0.0001. **g** Cell viability at 24hrs in WT and CRISPR/Cas9 BAX KO Calu-6 cell lines treated with Navitoclax alone in the presence of a fixed sensitizing concentration of BTSA1.2 (loss of viability <10%). Comparison of BAX and BAK protein expression levels in indicated cell lines. Data are mean ± SD of three technical replicates from *n* = 3 independent experiments. **h** Caspase 3/7 activity in WT and CRISPR/Cas9 BAX KO Calu-6 cell lines after 8 hrs treatment with Navitoclax alone and in combination with a fixed sensitizing concentration of BTSA1.2 (loss of viability <10%). Data are mean ± SD of three technical replicates from *n* = 3 independent experiments. Statistics were obtained using two-way ANOVA: *p < 0.05; **p < 0.01; ***p < 0.001; ****p < 0.0001. Source data are provided as a Source Data file.

We conducted a screen in our panel of cancer cell lines to compare the cytotoxic activity of Navitoclax with the cytotoxic activity of Navitoclax in combination with a fixed sensitizing concentration of BTSA1.2. The combination treatment of Navitoclax with a fixed sublethal dose of BTSA1.2 increased cytotoxicity in many cancer cell lines including solid tumors such as pancreatic and colorectal carcinomas regardless of common genetic alterations (e.g., TP53, RAS) (Fig. 2a–c and Supplementary Fig. 8a). Depending on the fold change of the IC_50_, cell lines were categorized as sensitive or resistant to the combination. Cell lines sensitive to the combination were predicted to have a synergistic effect upon the dual treatment. Indeed, in cell lines from different tumor types, upon dual treatment, cell viability was synergistically decreased across different concentrations (Fig. 2d, e and Supplementary Fig. 9a, b). Consistent with a synergistic effect on apoptosis induction, a significant increase of caspase 3/7 activation was observed upon dual treatment compared to the activity of single agents (Fig. 2f). Importantly, the synergistic effect in loss of viability and induction of apoptosis was determined to be BAX-dependent, as Calu-6 cells that are sensitive to the BTSA1.2 and Navitoclax combination (BN_C_), become resistant to the combination when these cells lack BAX expression (Fig. 2g, h). However, Calu-6 BAX KO cells are still sensitive to a generic apoptosis inducer Staurosporine presumably through BAK-mediated apoptosis (Supplementary Fig. 9c, d). Thus, the BN_C_ suggests a promising therapeutic strategy as these compounds synergize to promote apoptosis in solid tumors and hematological malignancies regardless of the mutational background.

To further dissect the contribution of BCL-XL or BCL-2 inhibition, since Navitoclax is able to inhibit both proteins in cells, we investigated both selective BCL-XL inhibitor A-1331852 and selective BCL-2 inhibitor Venetoclax in combination with the BAX activator BTSA1.2. We evaluated a set of cell lines from solid tumors e.g., SW480, COLO230 and hematological malignancies OCI-AML3, U937 in which expression for either BCL-XL or BCL-2 or both proteins can be detected (Supplemental Fig. 4a). In SW480 and COLO230 cell lines that BCL-2 protein expression is not detected by western blot and BCL-XL is well expressed, Venetoclax is not effective at submicromolar concentrations and synergy is not observed with Venetoclax and BTSA1.2 combination (Supplementary Fig. 10a, b). In contrast, BCL-XL specific inhibitor A-1331852 is potent in SW480 cell line and shows strong synergy with BTSA1.2 in SW480 (Supplementary Fig. 10a). A-1331852 is not effective in COLO230 presumably due to MCL-1 (Supplemental Fig. 4a) but it demonstrated synergy with BTSA1.2 (Supplementary Fig. 10b). Furthermore, in OCI-AML3 and U937 cells where BCL-2 is expressed, Venetoclax is effective as single agent and demonstrated synergy when combined with BTSA1.2 (Supplementary Fig. 10c, d). More specifically, Venetoclax is more effective and synergistic with BTSA1.2 in OCI-AML3 cells than in U937 cells, most likely because OCI-AML3 is more dependent on BCL-2 protein and has higher BCL-2 protein levels (Supplemental Fig. 4a). A-1331852 is moderately potent as single agent in OCI-AML3 and U937 cells but it demonstrates also synergy with the BTSA1.2 combination (Supplementary Fig. 10c, d). These data support that BCL-XL is the anti-apoptotic protein that controls BAX in majority of resistant solid tumor cell lines as BCL-2 is not detected or BCL-2 inhibition has limited effect in these cell lines.

### BAX interaction with BCL-XL dictates sensitivity to BTSA1.2 and Navitoclax combination

**Fig. 3:**
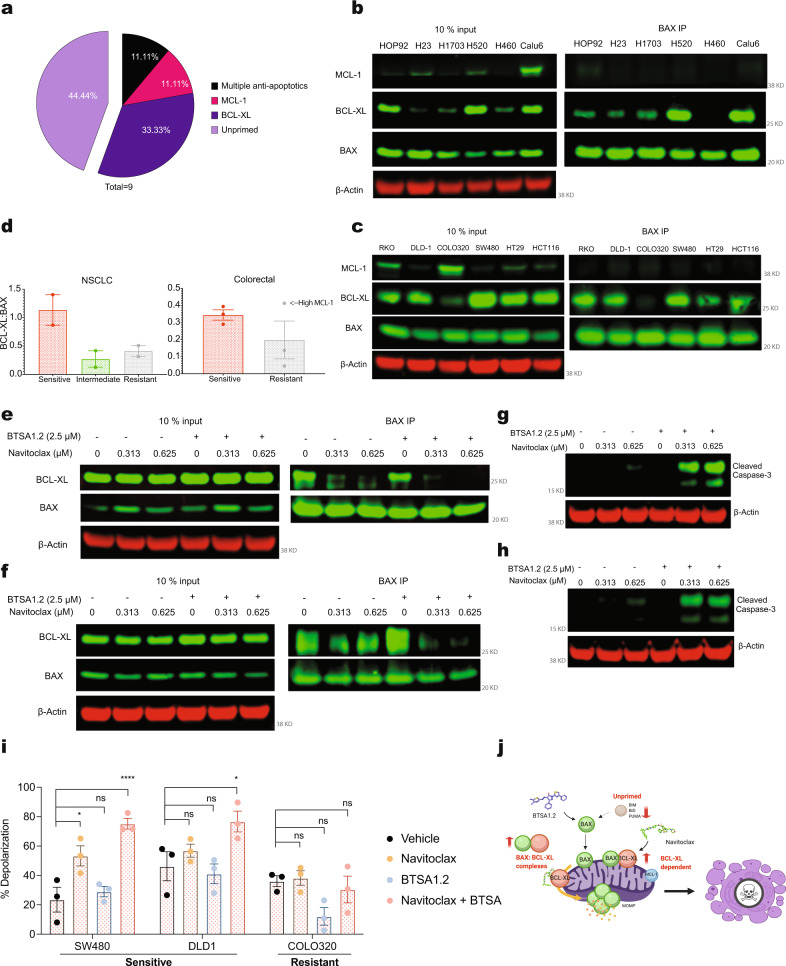
BAX interaction with BCL-XL dictates sensitivity to BTSA1.2 and Navitoclax combination. **a** BH3-profiling predicts apoptotic blocks correlated with sensitivity to the BTSA1.2 and Navitoclax combination. **b**–**c** Western blot analysis of BAX Co-IP in **b**, NSCLC and **c** colorectal cell lines. Representative blot from *n* = 2 independent experiments. **d** Quantification of co-immunoprecipitated BAX with BCL-XL in cell lines according to the BTSA1.2 and Navitoclax combination activity (corresponding to Fig. 2a–c). Data is the the normalized values obtained from **b**, **c** ± SD from *n* = 2–3 independent experiments **e**, **f**, Western blot analysis of BAX IP in **e**, NSCLC cell line Calu-6 and **f**, colorectal cell line SW480 after 4 h treatment with BTSA1.2 and Navitoclax. **g**, **h** Detection of cleaved Caspase-3 apoptotic marker by western blot analysis in **g**, NSCLC cell line Calu6 and **h**, colorectal cell line SW480 after 4 h treatment with BTSA1.2 and Navitoclax. **i** Dynamic BH3-profiling of solid tumor cell lines treated with vehicle, Navitoclax, BTSA1.2, or the combination. Bar graph represent % of mitochondria depolarization of cancer cells detected by JC-1 upon treatment with BIM-BH3 derived peptide. Data are mean ± SD of three technical replicates from n = 2 independent experiments. **j** Schematic of sensitive cells to the BTSA1.2 and Navitoclax combination. Western blot data are a representative of at least three independent experiments. Source data are provided as a Source Data file.

To identify determinants of sensitivity for the BN_C_, we looked at the BCL-2 family protein expression and interactions. BH3-profiling indicated that cell lines sensitive to the combination were categorized as anti-apoptotic BCL-XL dependent or unprimed to apoptosis (Fig. 3a). Interestingly, these survival mechanisms were adopted by resistant cell lines to either single agent treatment of Navitoclax or BTSA1.2 (Fig. 1h, i), indicating that the dual targeting of BAX and BCL-XL was able to overcome these two-survival mechanisms in previously categorized resistant cells to single agent treatment.

We next evaluated how the BCL-2 family is modulated in sensitive cells to the BN_C_. When we examined protein expression levels of BCL-2 family members, only BAX:BCL-XL levels marginally correlated with the sensitivity towards the combination (Supplementary Fig. 10e). This finding suggested that interactions among BCL-2 family members, and not the protein levels, could be regulating sensitivity towards the combination. To dissect this, we co-immunoprecipitated BAX and interrogated its binding with BCL-XL and MCL-1 anti-apoptotic proteins in several colorectal and NSCLC (Fig. 3b, c and Supplementary Fig. 5e, f). Sensitive cell lines, in general, formed higher levels of BAX:BCL-XL complexes than cell lines resistant to the combination (Fig. 3d). The BAX complexes formed are consistent with the finding that BCL-XL is a key player in the apoptotic resistance of these cancer cell lines as immunoprecipitated BAX had more interactions with BCL-XL (Figs. 1b, 3b, c). In colorectal and NSCLC cell lines, upon treatment with Navitoclax the BAX:BCL-XL complexes were disrupted hence relieving the suppression of active BAX by BCL-XL (Fig. 3e, f). However, only upon the combination treatment with BTSA1.2, additional complexes were disrupted, and apoptosis induction was observed by caspase-3 activation (Fig. 3g, h). Consistently, apoptotic priming with activator BIM BH3 peptide was increased upon the combination treatment in sensitive cell lines but not on resistant cells (Fig. 3i). Thus, our data is consistent with the interaction of BCL-XL with BAX as determinant of the synergistic apoptotic efficacy of the BN_C_ (Fig. 3j).

### Combination of BTSA1.2 and Navitoclax is well tolerated in vivo

To investigate the therapeutic potential of BN_C_ in vivo, firstly we determined a maximum tolerated dose (MTD) of BTSA1.2 in mice. A MTD study was performed following a standard MTD protocol that included a daily dose of BTSA1.2 with concentrations ranging from 50 mg/kg/po to 300 mg/kg/po for 5 days and monitoring for 14 days (Supplementary Fig. 11a). The MTD study indicated that oral administration of BTSA1.2 is well tolerated up to 200 mg/kg without dose limiting toxicity (DLT at 300 mg/kg), where BTSA1.2 treated mice showed constant body weights and organs examined were between normal histologic limits (Supplementary Fig. 11b, c). Thus, BTSA1.2 is a BAX activator that can be safely administrated orally and has desirable pharmacokinetics to address therapeutic efficacy^38,44^.

**Fig. 4:**
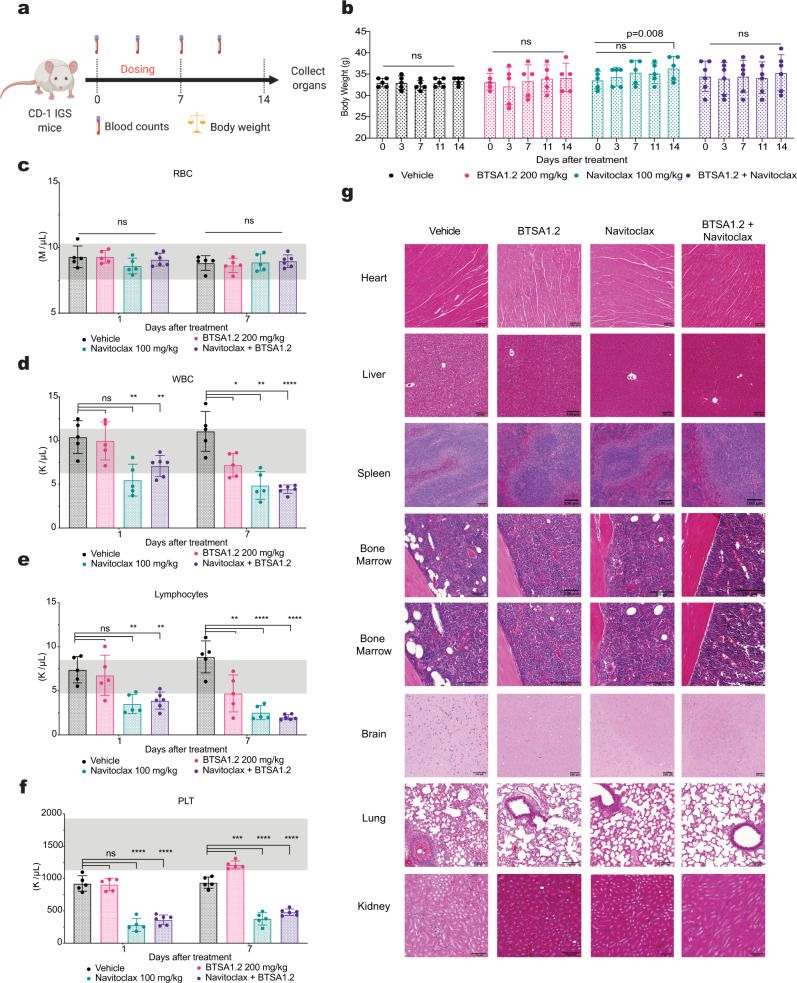
Combination of BTSA1.2 and Navitoclax is well tolerated and does not enhance Navitoclax driven toxicity in the hematopoietic system. **a** Schematic of BTSA1.2 and Navitoclax combination toxicity study. **b** Body weight measurements of CD1-IGS mice at 0, 3, 7, 11, and 14 days after the first treatment with vehicle, 100 mg/kg Navitoclax, 200 mg/kg BTSA1.2 or the combination. **c**–**f** Counts of peripheral **c**, red blood cells; **d** white blood cells; **e** lymphocytes; and **f** platelets in CD1-IGS mice treated with vehicle, 100 mg/kg Navitoclax, 200 mg/kg BTSA1.2 or the combination at 1 and 7 days after treatment. Normal blood counts range for CD-IGS male mice are indicated in gray. Data in **b**–**f**, represent mean ± SD from *n* = 5 mice (Vehicle, BTSA1.2 and Navitoclax) or *n* = 6 mice (Combination). Statistics were obtained using one-way ANOVA: *p < 0.05; **p < 0.01; ***p < 0.001; ****p < 0.0001. **g** Representative tissue sections heart, liver, spleen, bone marrow, brain, lung, and kidney using Hematoxylin and Eosin (H&E) staining from mice after treatment of vehicle, 100 mg/kg Navitoclax, 200 mg/kg BTSA1.2 or the combination. Scale bars, 100 μm. Data is representative of *n* = 3 independent samples per group. Source data are provided as a Source Data file.

We then conducted a toxicity study for the BN_C_ using their respective MTDs, 200 mg/kg/po for BTSA1.2 and 100 mg/kg/po for Navitoclax as previously determined^18^ (Fig. 4a). While body weight, red blood cells counts and organs examined were between the normal parameters, lymphocytes, white blood cells and platelets counts reached levels below normal counts upon a single treatment with Navitoclax (Fig. 4b–g) as previously described^18,45,46^, and blood counts recovered to normal levels after treatment completion (Supplementary Fig. 12a–c). Upon BTSA1.2 treatment body weight and blood counts were measured in normal levels but reduction in white blood cells and lymphocytes counts was observed after repeated dosing. Co-administration of BTSA1.2 and Navitoclax was well tolerated and no additional toxicity was observed in body weights, organs and blood counts compared to single-agent treatment (Fig. 4b–g). Interestingly, BTSA1.2 did not further potentiate Navitoclax-driven toxicity on platelets, lymphocytes, and white blood cell counts (Fig. 4d–f). Furthermore, blood counts recovered to normal levels after concluding the treatments (Supplementary Fig. 12a–c). Thus, our data suggest that the BN_C_ is well tolerated in vivo.

### BTSA1.2 and Navitoclax combination is efficacious in resistant colorectal xenografts

BCL-XL plays a key role in colorectal tumors formation and therapy resistance^47–50^. However, there is no clinical testing of BH3-mimetics for colorectal tumors as preclinical studies suggest that only BCL-XL inhibition is not sufficient to effectively induce apoptosis^47–50^. Here, we found that BN_C_ was able to promote apoptosis in colorectal tumors in vitro. Thus, to evaluate the therapeutic efficacy of the combination in vivo, we selected to evaluate the colorectal SW480 cells in xenograft mouse models, since in vitro SW480 cells were not sensitive to either BTSA1.2 or Navitoclax single agent treatments but sensitive to their combination (Fig. 2d, e).

**Fig. 5:**
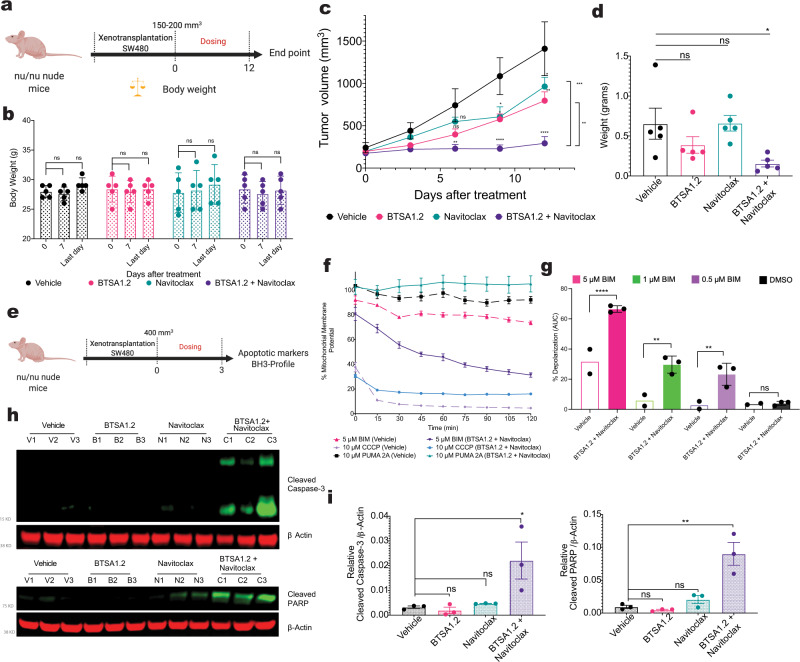
Combination therapy of BTSA1.2 and Navitoclax shows potent efficacy in resistant colorectal tumor xenografts. **a** Schematic of SW480 xenograft efficacy study. **b** Body weight measurements of *Nu/Nu* mice at 0, 7, and last day of treatment with vehicle, 100 mg/kg Navitoclax, 200 mg/kg BTSA1.2 or the combination. **c** Tumor volume curves of vehicle, Navitoclax, BTSA1.2 or the combination cohorts. **d** Tumor weight after completing study. Data in **b**–**d**, represent from *n* = 5 mice (Vehicle, BTSA1.2 and Navitoclax) or *n* = 6 mice (Combination). Statistics were obtained using two-way Anova: *p < 0.05; **p < 0.01; ***p < 0.001; ****p < 0.0001. **e** Schematic of SW480 pharmacodynamic xenograft study. **f** Example of kinetic curve of mitochondria potential in tumors treated with vehicle or combination upon stimuli of BH3-BIM peptide, Puma2A, CCCP or Alamethicin. Data are mean ± SD from *n* = 3 **g**, Dynamic BH3-profiling of tumors from mice treated with vehicle or BTSA1.2 and Navitoclax combination. Bar graph represent % of mitochondria depolarization of tumor cells detected by JC-1 upon treatment with BH3-BIM derived peptide or DMSO. Each point corresponds to the mean of *n* = 3 technical replicates; ± SD from *n* = 2 independent vehicle mice or *n* = 3 independent combination mice. Statistics were obtained using two-way Anova: *p < 0.05; **p < 0.01; ***p < 0.001; ****p < 0.0001. **h**, **i** Detection of cleaved Caspase-3 and cleaved PARP apoptotic markers by western blot analysis from SW480 tumors. Relative protein levels were normalized to β-Actin loading control. Data are mean ± SD from *n* = 3 mice. Statistics were obtained using two-way Anova: *p < 0.05; **p < 0.01; ***p < 0.001; ****p < 0.0001. Source data are provided as a Source Data file.

Once xenografts were established, mice were randomly divided into four groups for treatment with vehicle, BTSA1.2, Navitoclax and the combination. Treatments started when tumors reached a volume of ~200 mm^3^ using a daily oral administration with the MTD dose (Fig. 5a). While BTSA1.2 or Navitoclax as single agents had limited efficacy in reducing tumor growth, oral co-administration of BTSA1.2 and Navitoclax was able to significantly suppress tumor growth compared to vehicle or single agent treatments, accounting for the synergistic activity of the two drugs (Fig. 5b–d). Importantly, body weights remained constant during the in vivo study period and mice appeared healthy after treatment with the compounds (Fig. 5b).

To further confirm the synergistic efficacy of the two pro-apoptotic drugs in vivo, we conducted a pharmacodynamic analysis in which we assessed several apoptotic markers such as caspase-3 cleavage, PARP cleavage and mitochondrial depolarization in isolated tumors (Fig. 5e). Consistent with the efficacy study, we determined that only tumors treated with the BN_C_ exhibited significantly elevated apoptotic markers compared to vehicle or single agent treatments (Fig. 5f–i, Supplementary Fig. 12d, e). Thus, our data indicated that the BN_C_ can be synergistically efficacious in resistant colorectal xenografts to single agents.

### Functional markers identify sensitivity to the BTSA1.2 and Navitoclax combination in patient colorectal tumors

**Fig. 6:**
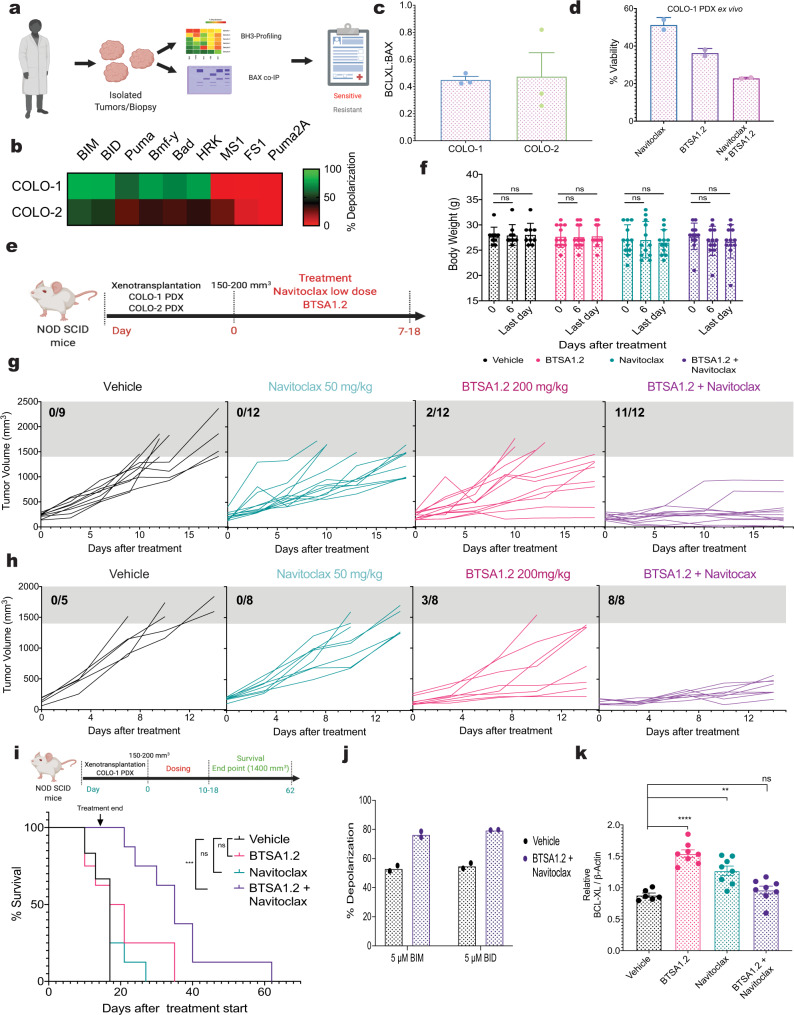
Functional markers identify tumors sensitive to the BTSA1.2 and Navitoclax combination. **a** Schematic of tumors characterization by BH3-profiling and BAX co-IP to predict efficacy of the BTSA1.2 and Navitoclax combination. **b** BH3-profiling of colorectal PDXs. Heatmap represent % of mitochondria depolarization of isolated tumor cells detected by JC-1 upon treatment with BH3-derived peptides. Data are mean ± SD from *n* = 3 independent experiments. **c** Quantification of co-immunoprecipitated BAX with BCL-XL in colorectal PDX (Supplementary Fig. 13b). Data are mean ± SD from *n* = 3 independent experiments. **d** Cell viability of COLO-1 PDX isolated cells after 24 h treatment with 1.25 µM Navitoclax, 10 µM BTSA1.2 or combination. Data are mean ± SD from *n* = 3 independent experiments. **e** Schematic of COLO-1 and COLO-2 PDX efficacy study. **f** Body weight measurements of *NOD SCID* mice at 0, 6, and last day of treatment with vehicle, 50 mg/kg Navitoclax, 200 mg/kg BTSA1.2 or the combination. Data is the mean ± SD from vehicle *n* = 9, BTSA1.2, Navitoclax, and combination *n* = 12 mice body weight. **g** Tumor volume curves of COLO-1 PDX vehicle, Navitoclax, BTSA1.2 or the combination cohorts. Data in **f**, **g**, represents individual measurements (vehicle *n* = 9, BTSA1.2, Navitoclax, and combination *n* = 12). **h** Tumor volume curves of COLO-2 PDX vehicle, Navitoclax, BTSA1.2 or the combination cohorts. Data represents individual measurements (vehicle *n* = 5, BTSA1.2, Navitoclax, and combination *n* = 8). **i** Survival of COLO-1 PDX after 18 days of treatment with vehicle, 50 mg/kg Navitoclax, 200 mg/kg BTSA1.2 or the combination, *n* = 8. **j** Dynamic BH3-profiling of COLO-1 tumors from mice treated with vehicle or BTSA1.2 and Navitoclax combination. Bar graph represent % of mitochondria depolarization of tumor cells detected by JC-1 upon treatment with BH3-BIM, BH3-BID or Puma2A derived peptide. Each data point is the mean of *n* = 3 technical replicates per samples; ± SD from *n* = 2 independent mice tumor samples. **k** Relative BCL-XL levels of COLO-1 tumors. Relative protein levels were normalized to β-Actin loading control. Data are mean ± SD from *n* = 8 mice. Statistics were obtained using one-way Anova: *p < 0.05; **p < 0.01; ***p < 0.001; ****p < 0.0001. Source data are provided as a Source Data file.

Our data indicated that cell lines categorized as sensitive to the BN_C_ were characterized by BH3-profiling as anti-apoptotic BCL-XL dependent or unprimed to apoptosis (Fig. 3a). Furthermore, BAX co-immunoprecipitation indicated that cell lines sensitive to the combination formed increased levels of BAX:BCL-XL complexes (Fig. 3b–d). Since these functional assays distinguished cancer cell lines sensitive and resistant to the BN_C_, we evaluated if these functional assays could be also used to predict efficacy of the BN_C_ in patient-derived xenografts (PDX) samples (Fig. 6a). Two colorectal PDX samples, COLO-1 and COLO-2, were analyzed. BH3-profiling designated COLO-1 as BCL-XL dependent and COLO-2 as unprimed for apoptosis while quantitative BAX co-immunoprecipitation indicated that both PDX samples had BAX:BCL-XL complexes (Fig. 6b, c and Supplementary Fig. 13a, b). As BN_C_ was effective in BCL-XL dependent and unprimed to apoptosis cancer cells, we predicted that COLO-1 and COLO-2 PDX samples should be sensitive to the combination treatment. Indeed, treatment of the PDX samples ex vivo showed that the BN_C_ induced more loss of viability when compared to single agents in both PDX samples (Fig. 6d and Supplementary Fig. 13c, d).

To further investigate these ex vivo results, we evaluated therapeutic efficacy of the BTSA1.2 and Navitoclax combination in vivo using mouse PDX models from COLO-1 and COLO-2 tumors. After PDX models were established, mice were randomly divided into four groups for treatment with vehicle, BTSA1.2, Navitoclax and the combination, and treatments started when tumors reached a volume of ~200 mm^3^. Compounds were administered orally, using for BTSA1.2 the MTD, while this time a lower dose (half the MTD) of Navitoclax was tested as single agent and in combination treatment. Treatments continued for up to 18 days or until tumor size reach an ethically unacceptable levels (Fig. 6e). The combination of BTSA1.2 with a low dose of Navitoclax was able to significantly suppress tumor growth and achieve tumor regression compared to the vehicle, BTSA1.2 or Navitoclax treatment while it was well tolerated (Fig. 6f–h). Notably, several PDX showed to respond to BTSA1.2 single agent treatment but none were sensitive to the Navitoclax single agent treatment.

Consistent with the tumor growth data, the combination of BTSA1.2 and Navitoclax was also able to significantly increase survival compared to vehicle or single agent treatments after conclusion of treatment (Fig. 6i). Moreover, PDX tumors treated with the BN_C_ had increased mitochondrial depolarization, compared to vehicle treatment PDX, which confirmed the pro-apoptotic efficacy of the combination (Fig. 6j). Interestingly, tumors of mice treated with single agent BTSA1.2 or Navitoclax had a significant increase of BCL-XL protein levels while MCL-1 levels remained constant (Fig. 6k, Supplementary Fig. 13e). This analysis further supports the in vitro data which indicated that BCL-XL upregulation confers resistance to single agent BTSA1.2 or Navitoclax treatment (Figs. 1, 3c, d).

Taken together, BH3-profiling and BAX co-immunoprecipitation functional assays were able to correctly predict the sensitivity to the BN_C_. The data suggest that BAX:BCL-XL complexes as well as BCL-XL dependent and unprimed for apoptosis criteria based on BH3-profiling method could be useful as sensitivity markers for this combination therapy. Furthermore, these studies demonstrated the therapeutic efficacy of the BN_C_ in colorectal PDX models using even a lower dose for Navitoclax that has less toxicity on platelet counts^46,51^.

### Genomic markers predict sensitivity or resistance to the BTSA1.2 and Navitoclax combination

**Fig. 7:**
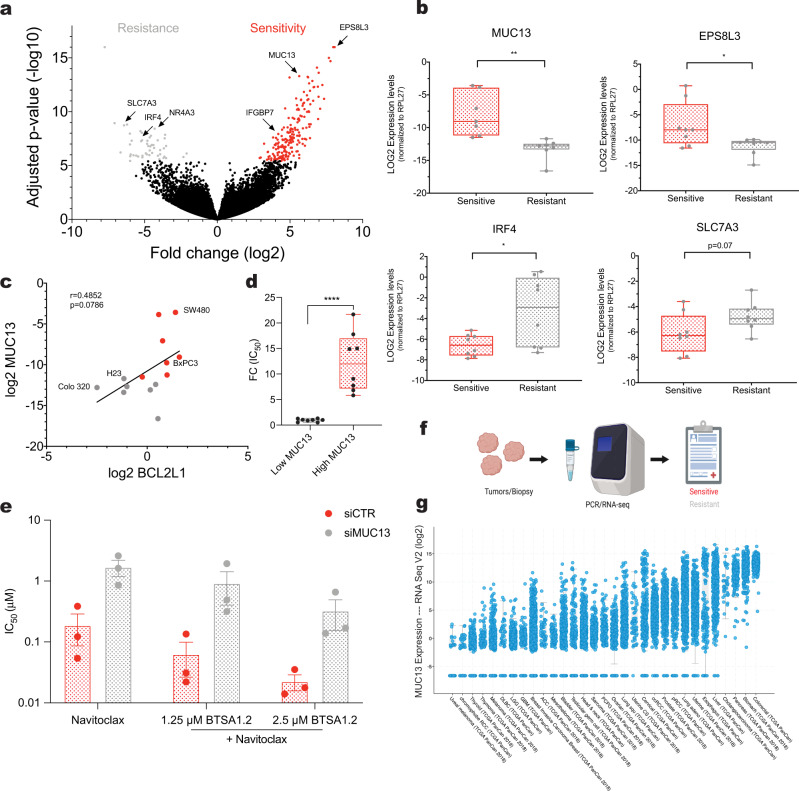
Bioinformatic analysis predicts markers of sensitivity and resistance to the BTSA1.2 and Navitoclax combination. **a** Volcano plot showing the expression change and significance level of genes between sensitive and resistant cell lines as defined based on the IC_50_ change from Navitoclax alone to BTSA1.2 and Navitoclax combination (corresponding to Fig. 2b). Top 250 predicted markers of sensitivity (red) and resistance (gray) are highlighted. **b** Box plot of validation of top hits associated with sensitivity and resistance to the BTSA1.2 and Navitoclax combination (corresponding to Fig. 2b) by RT-qPCR. Relative gene expression was normalized using RPL27. Analysis done in 7 sensitive cell lines and 6 resistant cell lines. The lines within the boxes show the median expression values, the box denotes the IQR, while the whiskers indicate maxima and minima values. Data are mean ± SD from *n* = 3 independent experiments. Statistics were obtained using two-tailed student t-test: *p < 0.05; **p < 0.01; ***p < 0.001; ****p < 0.0001. **c** Correlation of *BCL2L1 (*refers to BCL-XL protein*)* relative gene expression levels and *MUC13* gene expression levels in cell lines categorized as sensitive or resistant to the BTSA1.2 and Navitoclax combination (corresponding to Fig. 2b) using Pearson-Correlation. **d** Box plot of MUC13 expression grouped by the sensitivity to the BTSA1.2 and Navitoclax combination (corresponding to Fig. 2b). The lines within the boxes show the median MUC13 expression values, the box denotes the IQR, while the whiskers indicate maxima and minima values. Data are mean ± SD from n = 8 independent experiments. Statistics were obtained using two-tailed student t-test: *p < 0.05; **p < 0.01; ***p < 0.001; ****p < 0.0001. **e** Bar graph plot of the cell viability IC_50_ (μM) of SW480 after transfection with siRNA CTR or siRNA MUC13 and treated for 24 h with Navitoclax in combination with a constant sensitizing concentration of BTSA1.2 (loss of cell viability <20%). Data are mean ± SD from *n* = 3 independent experiments. **f** Schematic of various tumors analyzed for gene expression to predict sensitivity to the BTSA1.2 and Navitoclax combination. **g** MUC13 cancer patient’s expression data using TCGA and other non-redundant data from cbioportal.org. Statistics were obtained using student *t*-test: *p < 0.05; **p < 0.01; ***p < 0.001; ****p < 0.0001. Source data are provided as a Source Data file.

Identifying genomic biomarkers for sensitivity or resistance to the drug combination may provide information that could be useful for patient selection and further biological investigation. Having evaluated the BN_C_ in a diverse panel of solid tumors and hematologic malignancies with different mutational background (Fig. 2a, b) and realized the significant therapeutic efficacy of the combination in vivo in solid tumors (Figs. 5 and 6), we were interested in identifying genomic markers that can predict tumors' sensitivity to the drug combination treatment. For this, we conducted a bioinformatics analysis using gene expression analysis that is publicly available for several sensitive and resistant cell lines to the drug combination (Fig. 2b). This analysis identified significant differences in the gene expression between sensitive and resistant groups and ~250 hits were identified with high fold change and statistical significance (Fig. 7a, Supplementary Fig. 14a, Supplementary Table 4). We examined the top differentially expressed genes for their potential association with apoptosis, resistance to current treatments and/or poor cancer prognosis using literature and database searches and selected several genes for further validation. Genes that were highly expressed in sensitive cell lines *MUC13*, *EPS8L3* and *IGFBP7* were predicted as potentially markers of sensitivity to the BN_C_. While, genes such as *NR4A3*, *IRF4,* and *SLC7A3* which were highly expressed in resistant cell lines, were predicted as potential markers of resistance to the combination (Fig. 7a).

To confirm the correlation for these specific genes we selected sensitive and resistant cell lines to the BN_C_, and confirmed the higher expression either in sensitive or resistant cell lines by RT-qPCR (Fig. 7b and Supplementary Fig. 14b). Further analysis showed that the expression levels of the cell surface receptor gene *MUC13* showed significant correlation with BCL-XL gene expression levels (Fig. 7c). Therefore, higher expression of the *MUC13* marker correlates with the upregulation of BCL-XL which can also determine sensitivity to the BN_C_. Moreover, the levels of *MUC13* significantly correlated with the sensitivity to the BN_C_, as sensitive cells had significant higher expression of *MUC13* compared to resistant cells (Fig. 7d). To further evaluate MUC13 as a predictive marker of sensitivity to the combination, we knockdown (KD) the expression of MUC13 in a sensitive cell line to the combination. Data indicated that KD of MUC13 significantly increased the resistance to Navitoclax and the BN_C_ (Fig. 7e). These data suggest that higher MUC13 expression leads to high sensitivity to the BN_C_ and *MUC13* could be used as a prognosis marker for the BN_C_ (Fig. 7f). Notably, analysis of patient tumor data indicates that colorectal cancers and other solid tumors, such as pancreatic and stomach cancers, have higher levels of *MUC13*. Thus, patients with tumors having high MUC13 levels could be the ones to benefit most from treatment with the BN_C_ (Fig. 7g). Taken together, we were able to identify genomic markers to the BN_C_, which could be further examined as sensitivity or resistance biomarkers for this combination therapy.

## Discussion

Numerous studies have established the critical role of the BCL-2 family proteins in regulating apoptosis in tumor development, maintenance, and resistance to targeted therapies and chemotherapy^52^. Frequently, upregulation of the major anti-apoptotic members BCL-2, BCL-XL and MCl-1 has been established to suppress pro-apoptotic members and apoptosis^7–9^. To address this, potent and selective inhibitors of these proteins, termed BH3-mimetics, have been developed. These drugs have demonstrated activity against various hematological malignancies^16–19^. Indeed, Venetoclax, a selective BCL-2 inhibitor, has been the first BH3-mimetic approved for a subset of patients with Chronic Lymphocytic Leukemia or Acute Myeloid Leukemia^21,53,54^. Despite this success, several studies have shown that solid tumors are largely resistant to these drugs when used as single agents^24,26,27^. For these more resistant tumors either multiple anti-apoptotic BCL-2 proteins are upregulated and/or pro-apoptotic BH3-only proteins that are required to activate BAX and BAK for induction of apoptosis are kept suppressed^4,10–15^.

The development of small molecules that directly activate BAX represents a major advance in our ability to selectively promote apoptosis in cancer cells. BAX activators can induce apoptosis or potentiate apoptotic priming as they are not dependent on the availability of BH3-only protein activators. Here, we described BTSA1.2, an improved small molecule BAX activator from the previously described BTSA1, which has increased potency, favorable oral bioavailability and is well tolerated in vivo by oral administration^38^. BTSA1.2 demonstrated significant activity in leukemia and lymphoma cell lines, which is consistent with the significant efficacy as single agent therapy demonstrated for BTSA1 in human AML models^38^. However, similar to other BH3 mimetics including Navitoclax, the efficacy of BTSA1.2 in the tested solid tumor cell lines was reduced.

Our studies suggest that higher protein levels of BAX correlate with increased pro-apoptotic activity of BTSA1.2. This is consistent with the mechanism of BAX activation as increased BAX protein levels, can lead to more activated BAX by the small molecule BAX activator, and therefore to increased MOMP and apoptosis induction. On the other hand, BCL-XL was identified as a primary regulator of the BAX activation response particularly in solid tumor cell lines. BCL-XL and BCL-2 have higher affinity for BAX compared to MCL-1^55^. Therefore, in the absence of BCL-2 expression as evidenced in the majority of solid tumor cell lines, BCL-XL is the primary anti-apoptotic protein to sequester activated BAX. When both BCL-XL and BCL-2 proteins are expressed at similar levels, as evidenced mainly in hematological malignancies, then both proteins can regulate BAX activation. Testing of Navitoclax against the same diverse cell lines suggested that Navitoclax activity is primarily dependent on the levels of BAX and that targeting only BCL-XL in several cell lines is not enough to promote apoptosis. Furthermore, BH3-profiling highlighted that the majority of cells resistant to either BTSA1.2 or Navitoclax show dependency to BCL-XL and/or they are unprimed for apoptosis. Thus, our investigation of the mechanisms of apoptotic resistance in a range of malignancies, uncovered two survival mechanisms that limit direct and indirect BAX activation and apoptosis induction.

The combination of the BTSA1.2 and Navitoclax demonstrated synergistic activity in diverse solid tumor and hematologic cell lines that have dependency on BCL-XL and/or are unprimed to apoptosis. Of note, this synergistic activity was not affected by common oncogenic mutations such as TP53 or KRAS which typically limit the efficacy of chemotherapeutics and targeted therapies in cancer. Therefore, the combination of BTSA1.2 and Navitoclax could be applied more broadly to a variety of tumors.

By dissecting the mechanism of combined BAX activation and BCL-XL inhibition, we found that BAX:BCL-XL complexes are formed without treatment in the cell lines sensitive to the combination. Activation of cytosolic BAX by BTSA1.2 can promote additional BAX:BCL-XL complexes making these cells more primed to anti-apoptotic inhibition by Navitoclax or a BCL-XL selective inhibitor. On the other hand, Navitoclax or a BCL-XL selective inhibitor is capable to break BAX:BCL-XL complexes directly or indirectly using derepressed BH3-only proteins. In these cases, apoptotic activity from BCL-XL inhibition will depend on the levels of activated BAX bound to BCL-XL and the levels of BH3-only proteins bound to BCL-XL that can be derepressed to activate BAX. Therefore, the combined activity of a BAX activator and a BCL-XL inhibitor offers an effective strategy to induce apoptosis, by concurrently increasing the levels of activated BAX and inhibiting sequestration of activated BAX by BCL-XL, to enable increased MOMP and apoptosis induction.

The combination of the BTSA1.2 and Navitoclax demonstrated synergistic therapeutic efficacy in colorectal tumors while being remarkably tolerated in vivo, indicating that this therapeutic strategy may be highly promising for solid tumors. Previous evidence has shown that high expression levels of BCL-XL plays a key role in colorectal tumors formation and therapy resistance^47–50^. Despite such evidence, the application of Navitoclax in colorectal tumors including our work here suggests that BCL-XL inhibition is not efficient as single agent treatment and is not able to promote apoptosis. Previous studies have shown that Navitoclax synergize effectively with targeted therapies such as EGFR inhibitors in NSCLC and MEK inhibitors in KRAS mutant cancers and BRAF mutant melanoma^56–58^. These studies showed that targeting oncogenic driver pathways lead to increased BH3-only proteins, e.g., upregulation of BIM by MEK inhibitors, that enhance priming and efficacy of Navitoclax-mediated BCL-XL inhibition. Currently active clinicals trials are testing the efficacy of these combinations in patients (NCT03222609, NCT02079740, NCT01989585). However, these combination strategies rely on mutation of specific kinases to be effective. As in our studies we found that the mutational background of cancer cells did not affected the synergy between Navitoclax and BTSA1.2, this supports that this combination strategy could be effective in a variety of tumors. Furthermore, Navitoclax is hindered by its thrombocytopenia effect and a combination therapy in clinical trials will require an effective therapeutic window^22^. Therefore, the fact that our combination studies in vivo demonstrate a synergistic therapeutic efficacy with a reduced Navitoclax dose and overall a safe profile in tissues and blood counts, is noteworthy. In addition, efforts to develop clinical compounds that target BCL-XL with minimal toxicity on platelets are underway and these could be used alternatively to potentiate the pro-apoptotic activity of BTSA1.2^59^.

Our work identified functional assays and markers to predict sensitivity on concurrent BAX activation and BCL-XL inhibition, based on the BAX:BCL-XL complexes and BH3-profiling of cancer cells. Development of diagnostic assays for identification of BAX-containing protein complexes and BH3-profiling from solid tumor biopsies should be established next. Although this remains to be determined, our data on genomic analysis and identification of genes for sensitivity or resistance to the drug combination provide information that may be useful for biomarker selection. Our analysis identified high levels of *MUC13* to be a marker of sensitivity to the BTSA1.2 and Navitoclax combination, suggesting that this combination therapy could be beneficial for cancer patients with high levels of *MUC13*. Interestingly, *MUC13* has been proposed as a marker of poor prognosis in colorectal tumors^60^, supporting our findings for combined targeting of BAX and BCL-XL in resistant colorectal tumors. Furthermore, from a mechanistic stand-point our bioinformatic analysis informs future studies to investigate if and how markers such as *MUC13* may regulate the expression and interactions among the BCL-2 protein family and apoptosis induction.

In summary, our study advances our understanding of cell death mechanisms in cancer cells and demonstrates a novel therapeutic strategy, which rationally targets pro-apoptotic BAX and anti-apoptotic BCL-XL to overcome apoptosis resistance mechanisms in a range of tumors. Our findings provide preclinical proof-of-concept for the combination treatment of a new orally bioavailable BAX activator, BTSA1.2, and Navitoclax, which may provide a broad therapeutic effect in solid tumors and hematological malignancies.

## Methods

### Cell lines

Cell lines were purchased from ATCC and DSMZ. Head and neck cancer cell lines HN30, HN31, UMSCC6, MDA686LN, and HN5, were provided by Dr. Thomas Ow, and were originally obtained from a repository maintained by Dr. Jeffrey N. Myers, MD, PhD at the University of Texas, MD Anderson Cancer Center, with some cell lines requiring the following permissions (HN30, HN31—John Ensley, MD, Wayne State University; UMSCC6—Thomas Carey, University of Michigan; MDA686LN – Peter Sacks, MD, New York University/University of Texas MD Anderson Cancer Center). Ovarian, NSCLC, Colon, Leukemia, Lymphoma, BxPC-3 and ASPC1 cells lines were maintained in RPMI 1640 media Gibco) supplemented with 10% FBS, 100 U ml^–1^ penicillin/streptomycin, 2 mM l-glutamine, and 50 μM β-mercaptoethanol. Breast, Melanoma, HCT116, MIA PaCa-2 and HEY cell lines were maintained in DMEM (Gibco) supplemented with 10% FBS, 100 U ml^–1^ penicillin/streptomycin and 2 mM l-glutamine. Head and Neck cancer cell lines were maintained in DMEM (Gibco) supplemented with 10% FBS, 1X Vitamines, 1X sodium pyruvate, 1X nonessential amino acids, 100 U ml^–1^ penicillin/streptomycin and 2 mM l-glutamine. Capan-1 was maintained in Iscove’s Modified Dulbecco’s Medium (Gibco) supplemented with 10% FBS, 100 U ml^–1^ penicillin/streptomycin and 2 mM l-glutamine. Capan-2 was maintained McCoy’s 5a Medium Modified (Gibco) supplemented with 10% FBS, 100 U ml^–1^ penicillin/streptomycin and 2 mM l-glutamine. OCI-AML3 was maintained in MEM α Gibco) supplemented with 10% FBS, 100 U ml^–1^ penicillin/streptomycin, 2 mM l-glutamine and 50 μM β-mercaptoethanol.

### Mice

All animal experiments were approved by and performed in compliance with the guidelines and regulations approved by the Institutional Animal Care and Use Committee of the Albert Einstein College of Medicine (protocols 20161008, 20180707). Animals were housed in a controlled environment, target conditions: temperature 19–25 °C, relative humidity 30 to 60%. An electronic time-controlled lighting system was used to provide a 12 h light/12 h dark cycle. For toxicity studies 6–8 weeks old *CD1-IGS* male and female mice were purchased from Charles River. For xenograft studies and pharmacodynamics analysis experiments, 6–8 weeks old Nu/Nu nude mice and *NOD SCID* male mice were purchased from Charles River. All mice were kept under standard conditions and diet and had a weight > 20 grs.

### Patient-derived xenografts samples

Human colorectal tumor xenografts were obtained from Eduardo Vilar at The University of Texas, MD Anderson Cancer Center. Samples were obtained from two patients with metastatic colorectal cancer (Supplementary Table 5). Patients provided written informed consent for patient derived xenografts (PDX) under an IRB-approved protocol. Animal experiments using PDXs were performed according to the IACUC-approved protocols.

### Compounds

Hydrocarbon-stapled peptide corresponding to the BH3 domain of BIM, FITC-BIM SAHB_A2_: FITC-βAla-EIWIAQELRS5IGDS5F’NAYYA-CONH2, where S5 represents the non-natural amino acid inserted for olefin metathesis, was synthesized, purified at >95% purity by CPC Scientific Inc. BTSA1.2, BTSA1, BAM7, and Compound 6 were synthesized at the Albert Einstein College of Medicine >98% purity. The synthesis and analytical characterization of BTSA1.2 is provided in Supplementary Data. BTSA1 and Compound 6 was synthesized and characterized, as previously described^39^. BAM7 was synthesized and characterized as previously described^37^. Compound 4 and Compound 5 were provided by Chembridge (Cat # 5705811) and Molport (MolPort-004-904-917) at > 95% purity. Navitoclax was purchased from MedCheM Express (99.97% purity) for in vivo studies and SelleckChem (99.53% purity) for in vitro studies. A-1331852 was purchased from SelleckChem (99.8% purity), Venetoclax was purchased from SelleckChem (99.7%) and Staurosporine (99.61% purity). The following BH3 peptides were purchased from Genscript at >95% purity. Peptides had an acetylation as a N-terminal modification and an amidation as a C-terminal modification. Compounds were reconstituted in 100% DMSO and diluted in aqueous buffers or cell culture medium for assays.

hBIM: Ac-MRPEIWIAQELRRIGDEFNA-NH2,

hBID-Y:Ac-EDIIRNIARHLAQVGDSMDRY-NH2,

mBAD: Ac-LWAAQRYGRELRRMSDEFEGSFKGL-NH2,

HRK-y: Ac -SSAAQLTAARLKALGDELHQY- NH2,

mNoxaA: Ac-AELPPEFAAQLRKIGDKVYC-NH2,

Puma: Ac-EQWAREIGAQLRRMADDLNA-NH2,

Bmf-Y: Ac-HQAEVQIARKLQLIADQFHRY-NH2,

Puma2A: Ac-EQWAREIGAQARRMAADLNA-NH2,

MS1: Ac-RPEIWMTQGLRRLGDEINAYYAR-NH2,

FS1: Ac-QWVREIAAGLRLAADNVNAQLER-NH2.

### Cell viability assay

Cancer cells (1–2 × 10^3^ cells/well) were seeded in 384-well white plates and incubated with serial dilutions of BAX activator compounds including BTSA1.2, Navitoclax, A-1331852, Venetoclax, Staurosporine or vehicle (1% DMSO) in no FBS media for 2 h (except Staurosporine), followed by 10% FBS replacement to a final volume of 25 μL. Cell viability was assayed at 72 h by addition of CellTiter-Glo Assay reagents according to the manufacturer’s protocol (Promega), and luminescence measured using a F200 PRO microplate reader (TECAN). For Navitoclax and BTSA1.2 combination, A-1331852 and BTSA1.2 combination and Venetoclax and BTSA1.2 combination experiments, cells were seeded as described above and co-treated with Navitoclax or A-1331852 or Venetoclax and BTSA1.2 at the indicated doses. Excluding high-throughput drug screenings, viability assays were performed in at least triplicate and the data normalized to 1% vehicle-treated control wells. IC_50_ values were determined by nonlinear regression analysis using Prism software (Graphpad). Dilutions of compounds was performed using a TECAN D300e Digital Dispenser from 10 mM stocks. The BLISS calculation was determined using the Combenefit program as previously described^61^.

### Production of recombinant BAX protein

Human, recombinant and tagless BAX was expressed in Escherichia coli and purified as previously reported^62^. BAX wild type was purified by size-exclusion chromatography in a buffer containing 20 mM HEPES pH 7.2, 150 mM KCl, 1 mM DTT. Superdex 75 10/300 GL and 200 10/300 GL (GE Healthcare) columns were used.

### Fluorescence polarization binding assays

Fluorescence polarization assays (FPA) were performed as previously described^37^. Firstly, direct binding isotherms were generated by incubating FITC-BIM SAHBA_2_ (50 nM) with serial dilutions of full-length BAX and fluorescence polarization was measured at 20 min on a F200 PRO microplate reader (TECAN). Subsequently, in competition assays, a serial dilution of small-molecule or acetylated BIM SAHBA_2_ (Ac-BIM SAHB) was combined with FITC-BIM SAHBA_2_ (50 nM), followed by the addition of recombinant protein at EC_75_ concentration, as determined by the direct binding assay (BAX: 500 nM). EC_50_ and IC_50_ values were calculated by nonlinear regression analysis of competitive binding curves using Graphpad Prism software.

### Stability in liver microsomes assay

Male CD-1 mouse liver microsomes (Lot# 1510043) were purchased from XenoTech. The reaction mixture, minus NADPH, was prepared as follows: Liver Microsomes 0.5 mg/mL, NADPH (cofactor) 1 mM, Potassium Phosphate, pH 7.4 100 mM, Magnesium Chloride 5 mM, Compound 1 μM. Each compound was added into the reaction mixture at a final concentration of 1 μM. The control compound, testosterone, was run simultaneously with each compound in a separate reaction. An aliquot of the reaction mixture (without cofactor) was equilibrated in a shaking water bath at 37 °C for 3 min. The reaction was initiated by the addition of cofactor, and the mixture was incubated in a shaking water bath at 37 °C. Aliquots (100 μL) were withdrawn at 0, 10, 20, 30, and 60 min of each compound and 0, 10, 30, and 60 min for testosterone. Compound and testosterone samples were immediately combined with 400 μL of ice-cold 50/50 acetonitrile/H2O containing 0.1% formic acid and internal standard to terminate the reaction. The samples were then mixed and centrifuged to precipitate proteins. All samples were assayed by LC-MS/MS using electrospray ionization. Analytical conditions are outlined in Appendix 1. The peak area response ratio (PARR) to internal standard was compared to the PARR at time 0 to determine the percent remaining at each time point. Experiments were performed at Absorption Systems laboratory (Exton, PA).

### Western blotting

Protein lysates were obtained by cell lysis in 1% NP-40 buffer (50 mM Tris-HCL, 150 mM NaCl, 1 mM EDTA, 10% Glycerol, 1% NP-40, pH 7.50). Protein samples were electrophoretically separated on 4–12% NuPage (Life Technologies) gels, transferred to mobilon-FL PVDF membranes (Millipore), and subjected to immunoblotting. For visualization of proteins with Odyssey Infrared Imaging System (LI-COR Biosciences) membranes were blocked in Odyssey Blocking Buffer (LI-COR Biosciences). Primary antibodies were incubated overnight at 4 °C in a 1:1,000 dilution. After washing, membranes were incubated with an IRDye800-conjugated goat anti-rabbit IgG (1:10,000 dilution) or IRDye800-conjugated goat anti-mouse IgG (1:20,000 dilution) or IRDye680RD-conjugated goat anti-Rabbit IgG (1:20,000 dilution) secondary antibodies (LI-COR Biosciences). Proteins were detected with Odyssey Infrared Imaging System. Antibodies were used to detect the following proteins on membrane: BCL-XL (Cell Signaling Cat. 2762), MCL-1 (Cell Signaling Cat. 4572), BAX (Cell Signaling Cat. 2772), BCL-2 (BD. Cat. 610539), BAK (Millipore Cat. 06-536), BIM (Cell Signaling Cat. 2933 S), Cleaved Caspase-3 (Cell Signaling Cat. 9664 S), Cleaved PARP (Cell Signaling Cat. 5625 S), COX-IV (Cell Signaling Cat. 4850 S), β-Actin (Sigma Cat. A1978), β-Tubulin (Cell Signaling Cat. 2146 S).

### Whole cell immunoprecipitation and immunoblotting

Protein lysates were obtained by cell lysis in 0.2% NP-40 buffer (50 mM Tris-HCL, 150 mM NaCl, 1 mM EDTA, 10% Glycerol, 0.2% NP-40, pH 7.50). Immunoprecipitation was performed in 600 mL of 400 μg of proteins, which was precleared by centrifugation followed by exposure to 12 μL (50% slurry) protein A/G beads (Santa Cruz) at 4 °C for 30 min. Cleared extracts were incubated overnight with 2 μL of anti-BAX antibody (Cell Signaling Cat. 2772). Samples were then exposed to 20 μL (50% slurry) protein A/G beads (Santa Cruz) at 4 °C for 2 h and later centrifuged and washed three times with 0. 2% NP-40 buffer and boiled in loading buffer (Life Technologies). Protein samples were electrophoretically separated on 4–12% NuPage (Life Technologies) gels, transferred to mobilon-FL PVDF membranes (Millipore) and subjected to immunoblotting. For visualization of proteins with Odyssey Infrared Imaging System (LI-COR Biosciences) membranes were blocked in Odyssey Blocking Buffer (LI-COR Biosciences). Primary antibodies were incubated overnight at 4 °C in a 1:1,000 dilution. After washes, membranes were incubated with an IRdye800-conjugated goat anti-rabbit IgG or IRdye800-conjugated goat anti-mouse IgG secondary antibodies (LI-COR Biosciences) in a 1:10,000 and 1:20,000 dilution, respectively. Antibodies were used to detect the following proteins on membrane: BCL-XL (Cell Signaling Cat. 2762), MCL-1 (Cell Signaling Cat. 4572), BAX (Cell Signaling Cat. 2772), BCL-2 (BD. Cat. 610539), β-Actin (Sigma Cat. A1978).

### Cellular BAX translocation assay

Cells were seeded and incubated with serial dilutions of BTSA1.2 or vehicle (1% DMSO) in media with no FBS. After 2 h, FBS was supplemented to a final concentration of 10%. Following 4 h treatment, cells were lysed in 100 μL of digitonin buffer [20 mM Hepes, pH 7.2, 10 mM KCl, 5 mM MgCl2, 1 mM EDTA, 1 mM EGTA, 250 mM sucrose, 0.025% Digitonin (from 5% w/v stock) and complete protease inhibitors cocktail (Thermo-Fisher)] and incubated on ice for 10 min. The supernatants were isolated by centrifugation at 15,000 × *g* for 10 min and the mitochondrial pellets solubilized in 1% Triton X-100/PBS for 1 h at 4 °C. Pellets were solubilized, subjected to a 15,000 x rpm spin for 10 min, and 50 ng of protein was mixed with 25 μL LDS/DTT loading buffer. The equivalent fractional volume of the corresponding supernatant samples was mixed with 25 μL LDS/DTT loading buffer. The mitochondrial supernatant and pellet fractions were then separated by 4–12% NuPage (Life Technologies) gels, follow by analysis by immunoblotting with anti-BAX antibody (2772 S, Cell Signaling), BCL-XL (Cell Signaling Cat. 2762), MCL-1 (Cell Signaling Cat. 4572). COX-IV (Cell Signaling Cat. 4850 S) and β-Tubulin (Cell Signaling Cat. 2146 S) are used for loading control of mitochondrial and supernatant fractions, respectively.

### Cellular thermal shift assays (CETSA)

BxPC3 and SW480 cells were seeded in a 10-cm dish until ~85% confluent. The media was removed and replaced with media with no FBS, and cells were treated with 40 µM BTSA1.2 for 15 min at 37 °C. The media was removed, and cells were washed once with PBS and harvested using a cell scraper. BXPC3 or SW480 cells were resuspended in PBS to 10 × 10^6^ cells/mL and 50 µL was transferred to PCR tubes. Cells were then heated in a Biorad C1000 Touch Thermal Cycler for 3 min using a temperature gradient (50, 52.1, 55.4, 59.4, 64.9, 69.2, 72.1, and 74 °C). All cells were lysed by three cycles of freeze-thawing using liquid nitrogen. Samples were then centrifuged at 2 × 10^4^ × *g* for 15 min at 4 °C. The supernatants were collected, resolved by SDS-PAGE and analyzed by western blot with an N-terminal BAX antibody (Cell Signaling, 2772 S or Santa Cruz, 2D2/sc-20067) and a BAK antibody (Millipore, NT/06-536). Results were quantified by densitometric analysis using the Image Studio software and normalized to 25 °C (100%) and blot background (0%).

### Immunoprecipitation of digitonin-fractionated supernatant and mitochondrial extracts

Cells (10 × 10^6^ cells/well) were seeded in 100 mm dishes and incubated with serial dilution concentrations of BTSA1.2 or vehicle (0.2% DMSO) in media with no FBS in a final volume of 5 mL. After 2 h, FBS was supplemented to a final concentration of 10%. Following 4 hrs treatment, cells were lysed in 100 μL of digitonin buffer [20 mM Hepes, pH 7.2, 10 mM KCl, 5 mM MgCl_2_, 1 mM EDTA, 1 mM EGTA, 250 mM sucrose, 0.025% Digitonin (from 5% w/v stock) and complete protease inhibitors (Roche Applied Science)] and incubated on ice for 10 min. The supernatants were isolated by centrifugation at 15,000 × *g* for 10 min and the mitochondrial pellets solubilized in NP-40 lysis buffer (50 mM Tris-HCL pH 7.4, 150 mM NaCl, 5 mM MgCL_2_, 1  Mm EGTA, 10% Glycerol, 0.2% NP-40). Immunoprecipitation was performed in 600 μL of 500 μg of proteins from supernatant and mitochondrial pellet fractions. Briefly, fractions were pre-cleared by centrifugation after expose with 12 μL (50% slurry) protein A/G beads (Santa Cruz) at 4 °C for 1 h. Cleared extracts were incubated overnight with 1 μL of anti-BAX antibody (Cell Signaling Cat. 2772). Samples were then exposed to 20 μL (50% slurry) protein A/G beads (Santa Cruz) at 4 °C for 3 h and later centrifuged and washed three times (3,000 g for 1 minute) with NP-40 lysis buffer and boiled in loading buffer (Life Technologies) for 15 min. Protein samples were electrophoretically separated on 4–12% NuPage (Life Technologies) gels, transferred to mobilon-FL PVDF membranes (Millipore) and subjected to immunoblotting. For visualization of proteins with Odyssey Infrared Imaging System (LI-COR Biosciences) membranes were blocked in PBS containing 5% dry milk. Primary antibodies were incubated overnight at 4 °C in a 1:1,000 dilution. After washes, membranes were incubated with an IRdye800-conjugated goat anti-rabbit IgG or IRdye800-conjugated goat anti-mouse IgG secondary antibodies (LI-COR Biosciences) in a 1:5,000 dilution for 1 h. Antibodies were used to detect the following proteins on membrane: BCL-XL (Cell Signaling Cat. 2764 S), MCL-1 (Cell Signaling Cat. 4572), BAX (Cell Signaling Cat. 2772), β-Tubulin (Cell Signaling Cat. 86298 S), and COX IV (Cell Signaling Cat. 11967 S).

### Western blot protein quantification and pearson correlation

Densitometry of protein bands were acquired using a LI-COR Odyssey scanner. Quantification and analysis were performed using the Western Analysis tool from the Image Studio software. Relative expression levels were quantified based on protein expression of respective loading control: COX-IV, β-Actin, or β-Tubulin. Pearson correlation was determined using Prism software (Graphpad) comparing the cell viability IC_50_ for single agents and the combination of Navitoclax and BTSA1.2 values with the protein quantification for different members of the BCL-2 family of proteins.

### BH3-profiling

Cancer cell lines were compared by BH3 profiling under basal conditions. BIM BH3 (final concentrations of 10–0.1 μM), BID BH3, BMF-y, PUMA, BAD, HRK-y, and NOXA peptides (final concentrations of 10 μM); Puma2A peptide (final concentration of 20 μM); alamethicin (final concentration of 25 μM); CCCP (final concentration of 10 μM) were added to JC1-MEB staining solution (150 mM mannitol, 10 mM HEPES-KOH, 50 mM KCl, 0.02 mM EGTA, 0.02 mM EDTA, 0.1% BSA, 5 mM succinate, pH 7.5) in a black 384-well plate. Single cell suspensions were prepared in JC-1-MEB buffer, as previously described^63^. Cells were kept at room temperature for 10 min to allow for cell permeabilization and dye equilibration. After adding the cells to the 384-well plate, 1.0 × 10^4^ cells/well to 2.0 × 10^4^ cells/well, fluorescence was measured at 590 nm emission 545 nM excitation using the M1000 microplate reader (TECAN) at 30 °C every 15 min for a total of 3 h. Percentage of depolarization was calculated by normalization to the AUC of solvent-only control DMSO (0% depolarization) and the positive control CCCP (100% depolarization), as previously described^42^. For dynamic BH3-profiling cells were treated with the compounds at the indicated doses for 20 h. Mitochondria depolarization was compared at the same concentration of BID peptide for each treatment group.

### Caspase 3/7 activation assay

Cancer cells were treated with BAX activator compounds or Navitoclax or Staurosporine, at the indicated concentrations, as single agents or in combination as previously described in the cell viability assays. Caspase-3/7 activation was measured at 3 h in SU-DHL-5 cells and at 8 h in other cell lines for BTSA1.2 and Navitoclax and at 24 h for staurosporine by addition of the Caspase-Glo 3/7 chemiluminescence reagent in accordance with the manufacturer’s protocol (Promega). Luminescence was detected by a F200 PRO microplate reader (TECAN). Assays were performed in at least in triplicate.

### Pharmacokinetic analysis

*ICR* (*CD-1*) male mice fasted for at least 3 h and water was available ad libitum before the study. Animals were housed in a controlled environment, target conditions: temperature 18–29 °C, relative humidity 30 to 70%. Temperature and relative humidity were monitored daily. An electronic time-controlled lighting system was used to provide a 12 h light/12 hr dark cycle. 3 mice for each indicated time point were administered BTSA1.2 in 1% DMSO, 30% PEG-400, 65% D5W (5% dextrose in water), 4% Tween-80 either by an oral gavage (3 mg/Kg) or intravenous injection (1 mg/Kg). Mice were sacrificed, and plasma samples were harvested at 0 h, 0.25 h, 0.5 h, 1 h, 2 h, 4 h, 8 h, 24 h, and analyzed for BTSA1.2 levels using LC-MS/MS. Pharmacokinetics parameters were calculated using Phoenix WinNonlin 6.3. Experiments performed at SIMM-SERVIER joint Biopharmacy Laboratory.

### Maximum tolerated dose (MTD) and in vivo toxicity studies

A total of 6–8 weeks old *CD1-IGS* female and male mice (Charles River) were divided into six groups (*n* = 6 per arm), and treated with vehicle, 200 mg/kg BTSA1, 50 mg/kg, 100 mg/kg, 200 mg/kg or 300 mg/kg BTSA1.2 by oral gavage daily for 5 days. Mice were monitored daily and body weight was monitored on the indicated days. After 14 days of the first treatment, mice were subject to euthanasia and necropsy (Histology and Comparative Pathology Facility, Albert Einstein College of Medicine) and tissues (e.g., spleen, liver, kidney, lung, heart) were harvested for fixation in 10% buffered formalin (Fisher Scientific) for pathology analysis. Paraffin-embedded sections (5 mm) were stained with H&E. Peripheral blood from CD1-IGS mice was obtained by facial vein puncture and collected in EDTA-coated tubes (BD cat. 365973). Blood counts were determined on a Forcyte Veterinary Hematology Analyzer (Oxford Science Inc.). A total of 200 mg/kg BTSA1 and 300 mg/kg BTSA1.2 mice were subjected to necropsy studies, which determined they died by kidney failure after 3 days of treatment. Histological evaluation of tissues and necropsy performed by the Histology and Comparative Pathology Facility board-certified veterinary pathologist.

### BTSA1.2 and navitoclax combination in vivo toxicity studies

A total of 6–8 weeks old CD1-IGS male mice were purchased from Charles River. Mice were divided into four groups (vehicle, BTSA1.2 and Navitoclax *n* = 5, combination *n* = 6), and treated with vehicle, 200 mg/kg BTSA1.2, 100 mg/kg Navitoclax or BTSA1.2 and Navitoclax combination by oral gavage daily for 7 days. Mice in the combination group were first administered with 100 mg/kg Navitoclax and after 6–8 h were administered 200 mg/kg BTSA1.2. Mice were monitored daily; body weight and peripheral blood counts were monitored at the indicated days. After 14 days of the first treatment, mice were subject to euthanasia and necropsy (Histology and Comparative Pathology Facility, Albert Einstein College of Medicine) and tissues (e.g., spleen, liver, kidney, lung, heart, bone marrow, brain) were harvested for fixation in 10% buffered formalin (Fisher Scientific) for pathology analysis. Paraffin-embedded sections (5 mm) were stained with H&E. Peripheral blood from *CD1-IGS* mice was obtained by facial vein puncture and collected in EDTA-coated tubes (BD cat. 365973). Blood counts were determined on a Forcyte Veterinary Hematology Analyzer (Oxford Science Inc.).

### Tumor xenografts studies

A total of 6–8 weeks old *Nu/Nu* nude male mice were purchased from Charles River. Approximately, 2.5 × 10^6^ SW480 cells were suspended in cold PBS and injected subcutaneously into the right flanks of mice. Mice were divided into four groups (Efficacy study: vehicle, BTSA1.2 and Navitoclax *n* = 5, combination *n* = 6; Pharmacodynamic study: *n* = 3 for all groups), and treated with vehicle, 200 mg/kg BTSA1.2, 100 mg/kg Navitoclax or BTSA1.2 and Navitoclax combination by oral gavage daily. Mice in the combination group were first administered with 100 mg/kg Navitoclax and after 6–8 h were administered 200 mg/kg BTSA1.2. For efficacy the efficacy study, treatments started once tumors reached a volume of ~200 mm^3^. Tumor volume was monitored every 3 days by caliper measurements until the cessation of the experiment when tumors reached an ethically unacceptable size for the vehicle, BTSA1.2 or Navitoclax treated mice, for the mice administered the combination mice were euthanized the day after single agents or vehicle treated mice were euthanized. Body weight of mice were monitored during treatment. For the pharmacodynamic study, treatments started once tumors reached a volume of ~400 mm^3^, after 3 days of daily treatment mice were euthanized and tumors were collected for analysis.

### Patient-derived xenografts studies

A total of 6–8 weeks old *NOD SCID* male mice were purchased from Charles River. Approximately, 1.0 × 10^6^ COLO-1 or COLO-2 cells were suspended in a 1:1 DMEM:matrigel and injected subcutaneously into the right flanks of mice. PDX characterization: mice were divided into two groups COLO-1 and COLO-2 (*n* = 3) were divided into two groups. Tumor was collected once tumor reached a volume of ~1,000 mm^3^. COLO-1 efficacy study: Mice were divided into four groups (vehicle, BTSA1.2, Navitoclax and combination *n* = 4) and treated with vehicle, 200 mg/kg BTSA1.2, 50 mg/kg Navitoclax or BTSA1.2 and Navitoclax combination by oral gavage daily. Mice in the combination group were first administered with 50 mg/kg Navitoclax and after 6–8 h were administered 200 mg/kg BTSA1.2. Treatments started once tumors reached a volume of ~200 mm^3^. Tumor volume was monitored every 3–4 days by caliper measurements until the cessation of the experiment when tumors reached an ethically unacceptable size or after 18 days of daily treatment (whichever came first). Body weight of mice were monitored during treatment.

### Ex-vivo BH3 profiling

SW480 xenograft vehicle and combination treated tumors, and COLO-1 and COLO-2 PDX tumors were analyzed by BH3 profiling under basal conditions. Single cells from tumors were isolated by mechanically pass them through a 70 μM strainer filter with cold PBS. SW480 xenograft tumors: BIM BH3 and BID BH3, peptides (final concentrations of 10–0.5 μM); Puma2A peptide (final concentration of 10 μM); alamethicin (final concentration of 25 μM); CCCP (final concentration of 10 μM) were added to JC1-MEB staining solution (150 mM mannitol, 10 mM HEPES-KOH, 50 mM KCl, 1 mM EGTA, 1 mM EDTA, 0.1% BSA, 5 mM succinate, pH 7.5) in a black 384-well plate. PDX tumors: BIM BH3 and BID BH3, peptides (final concentrations of 25-1 μM); PUMA, BMF-y, BAD and HRK (final concentrations of 100–10 μM); MS1 and FS1 (final concentrations of 25–10 μM); and PUMA2A peptide (final concentration of 100–25 μM); alamethicin (final concentration of 25 μM); CCCP (final concentration of 10 μM) were added to JC1-MEB staining solution in a 384-well plate. Single cell suspensions were prepared in 1:1 JC-1-MEB buffer, as previously described, and were kept at room temperature for 10 min to allow for cell permeabilization and dye equilibration. After adding the cells to the 384-well plate, 2.0 × 10^4^ cells/well, fluorescence was measured at 590 nm emission 545 nM excitation using the M1000 microplate reader (TECAN) at 30 °C every 15 min for a total of 2 h. Percentage of depolarization was calculated by normalization to the AUC of negative control Puma2A (0% depolarization) and the positive control CCCP (100% depolarization), as described above. Mitochondria membrane potential was calculated by normalization of the AUC values to the AUC of negative control solvent-only 1% DMSO.

### Ex-vivo cell viability

Single cells from COLO-1 and COLO-2 PDX tumors were isolated by mechanically pass them through a 70 μM strainer filter with cold PBS. Isolated cells (10–20 × 10^3^ cells/well) were seeded in 384-well white plates and incubated with vehicle (1% DMSO) or serial dilutions of BTSA1.2, Navitoclax, or co-treated with Navitoclax and BTSA1.2 (at the indicated doses) in no FBS media for 2 h, followed by 10% FBS replacement to a final volume of 25 μL. Cell viability was assayed at 24 h by addition of CellTiter-Glo Assay reagents according to the manufacturer’s protocol (Promega), and luminescence measured using a F200 PRO microplate reader (TECAN). Viability assays were performed in at least duplicate and the data normalized to 1% vehicle-treated control wells. IC_50_ values were determined by nonlinear regression analysis using Prism software (Graphpad). Dilutions of compounds was performed using a TECAN D300e Digital Dispenser from 10 mM stocks.

### Bioinformatic analysis

We tested our drug candidates BTSA1.2 and Navitoclax separately and in combination on a total of 46 cancer cell lines. Cancer cell lines were defined as synergistic or non-synergistic to combination by the fold change from IC_50_ of Navitoclax to IC_50_ of Navitoclax and BTSA1.2 combined. Two groups were defined: (A) Synergistic group, IC_50_ fold change > =4; (B) Non-synergistic group, IC_50_ fold change <2. RNA-Seq raw counts data were retrieved from the Cancer Cell Line Encyclopedia (CCLE) database at BROAD Institute (https://portals.broadinstitute.org/ccle/data). A total of 23 cell lines (8 non-synergistic [Namalwa, REC1, Pfeiffer, RKO, H1703, COLO320, SKMEL30, and H23] and 15 synergistic [H520, Calu6, SUDHL4, SUDHL5, MIAPaCa2, HCT116, SW480, DLD1, HS578T, U937, HL60, OCIAML3, BxPC3, Capan1, ASPC1]) have RNA-Seq data from CCLE. Differential expression analysis was then conducted using DESeq2 (version 1.32.0) package in R comparing non-synergistic to synergistic group based on raw RNA-Seq data. Heatmap was generated for the top 150 differentially expressed genes comparing non-synergistic to synergistic cell lines group using pheatmap package in R. A literature search was done for bioinformatic analysis top hits, based on adjusted *p*-value, for genes which have been previously associated with: apoptosis, cancer treatment resistance, BCL-2 family or poor prognosis in cancer. After literature evaluation 8 top hits where selected for further validation by RT q-PCR.

### RNA preparation and real-time PCR

RNA from cells in culture was isolated using the E.Z.N.A total RNA Kit from Omega, following the manufacturer’s instructions. The quality and quantity of the RNA was determined by spectrophotometry using the NanoDrop 8000 Spectrophotometer from Thermo Scientific. For quantitative reverse transcription PCR (RT-qPCR), the RNA was reverse transcribed using the High-Capacity cDNA Reverse Transcription kit from Applied Biosystems, following the manufacturer’s instructions. PCR was performed using the PowerUp SYBR Green Master Mix from Applied Biosystems, on a ViiA 7 Real-Time PCR system from Applied Biosystems, following the manufacturer’s instructions. The cycling conditions included uracil-DNA glycosylase (UDG) activation for 2 min at 50 °C, then activation of the Dual-Lock Taq DNA polymerase for 2 min at 95 °C, followed by 40 amplification cycles consisting of 15 s of denaturation at 95 °C, 15 s of annealing at 60 °C, and 1 min of extension at 72 °C. The specificity of the amplified DNA was confirmed by performing a melting curve at the end of each RT-qPCR run. No template controls, containing all reaction components except the cDNA sample, were used to identify PCR contamination as this samples should not return a CT value. Gene expression results were normalized to the transcript amount of the ribosomal protein RPL27. The primers used for PCR were designed using the online NCBI Primer-BLAST tool. Each RT-qPCR was performed in at least triplicate. Primers were purchased from Eurofins Genomics (Supplementary Table 6).

### MUC13 transfection

Reverse transfection of siRNA MUC13 (Thermo Fisher cat. s32232, s32233) and siRNA negative CTR (Thermo Fisher cat. AM4636) was conducted in the SW480 cancer cell line. A total 50 nM of siRNA was diluted in Opti-MEM and mix gently. Lipofectamine RNAiMAX was added, mixed and incubated for 20 min at room temperature. Cells were added and incubated for 24 h and 48 h at 37 °C. For cell viability compounds were added after 24 h of transfection and cell viability was assessed after 24 h of treatment.

### Cancer patients gene expression data

Gene expression data for genes from cancer patients was obtained from cbioportal curated set of non-redundant studies.

### Quantification and statistical analysis

Plots and statistical tests were generated in GraphPad Prism 8.0–9.0 Data are presented as means ± SD except where noted. Statistical comparisons between two groups were performed using an unpaired two-way ANOVA, except where noted. *P* values indicated on the graphs: *p < 0.05, **p < 0.01, ***p < 0.001, ****p < 0.0001. Additional statistical details are provided in figure legends and methods details.

### Reporting summary

Further information on research design is available in the Nature Research Reporting Summary linked to this article.

## Supplementary information

Supplementary Information File

Reporting Summary

## Data Availability

RNA-Seq raw counts data were retrieved from the Cancer Cell Line Encyclopedia (CCLE) database at BROAD Institute (https://portals.broadinstitute.org/ccle/data). TCGA expression data were obtained from publicly available datasets at cBioPortal (https://www.cbioportal.org). All the information generated and analyzed is included in the manuscript and all figures have associated raw data that is provided as an Excel worksheet organized by figures (Data Source file). Source data are provided with this paper.

## Source data

Source Data
