# Supplementary material for: Co-targeting of BAX and BCL-XL proteins broadly overcomes resistance to apoptosis in cancer
Source: Nat Commun. 2022 Mar 7;13:1199. doi: 10.1038/s41467-022-28741-7 (PMC8901805; doi:10.1038/s41467-022-28741-7)
Supplement: Supplementary file 1 — Supplementary Information File [file 41467_2022_28741_MOESM1_ESM.pdf]

Supplementary Information

Co-targeting of BAX and BCL-XL proteins broadly overcomes resistance to apoptosis in cancer

Andrea Lopez [1-3#], Denis E. Reyna[1-3#], Nadege Gitego[1-3], Felix Kopp[1], Hua Zhou [4-6], Miguel A. Miranda-Roman[7-8], Lars Ulrik Nordstrøm[1], Swathi-Rao Narayanagari[9], Ping Chi [7,10-11], Eduardo Vilar[12], Aristotelis Tsirigos[4-6], Evripidis Gavathiotis[1-3,13*]

Supplementary Figures and Figure Legends 1-14

Supplementary Tables 1-6

Supplementary Methods

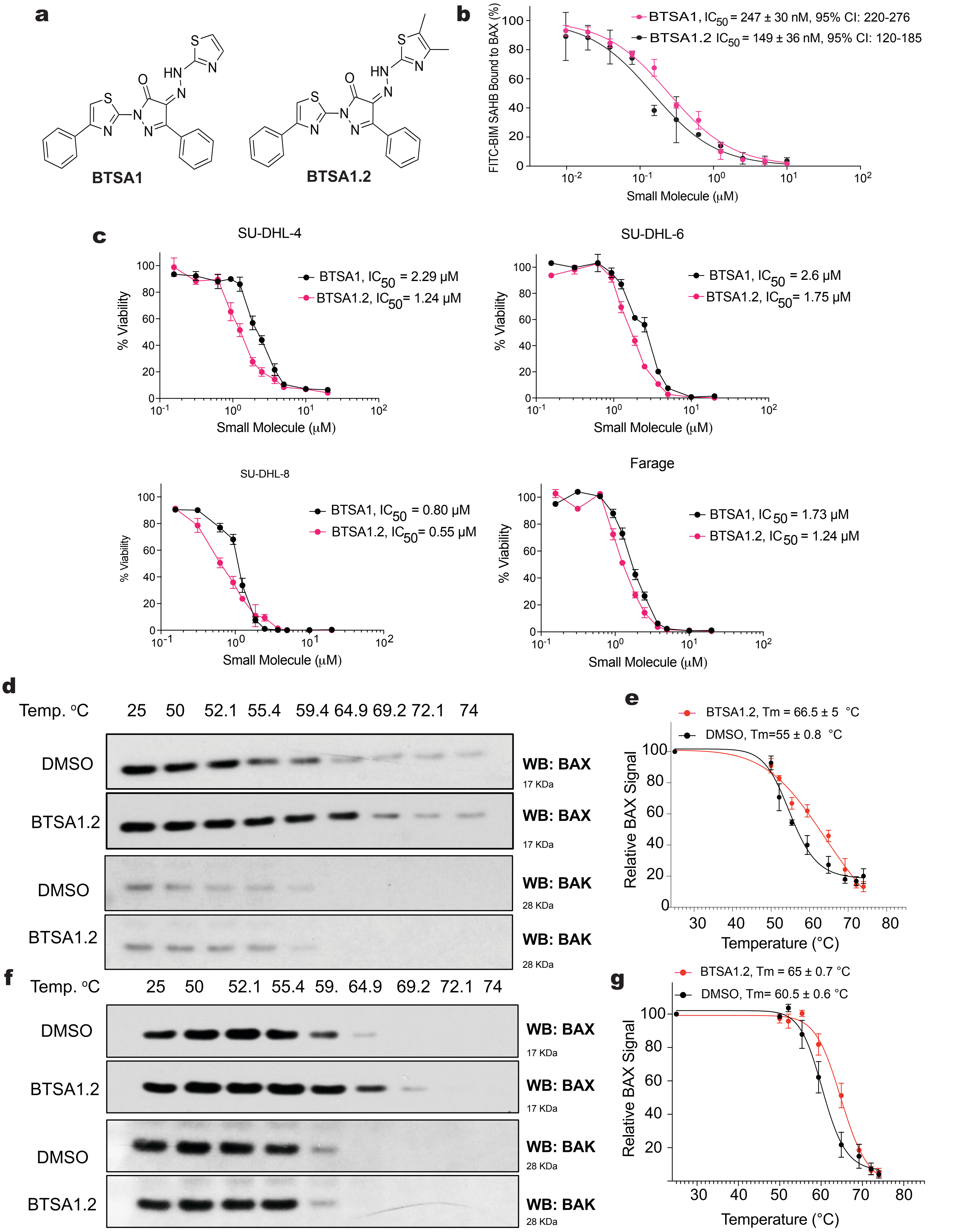
a
BTSA1
BTSA1.2
b
BTSA1, IC50 = 247 ± 30 nM, 95% CI: 220-276
BTSA1.2 IC50 = 149 ± 36 nM, 95% CI: 120-185
FITC-BIM SAHB Bound to BAX (%)
Small Molecule (μM)
c
SU-DHL-4
BTSA1, IC50 = 2.29 μM
BTSA1.2, IC50= 1.24 μM
SU-DHL-6
BTSA1, IC50 = 2.6 μM
BTSA1.2, IC50= 1.75 μM
SU-DHL-8
BTSA1, IC50 = 0.80 μM
BTSA1.2, IC50= 0.55 μM
Farage
BTSA1, IC50 = 1.73 μM
BTSA1.2, IC50 = 1.24 μM
% Viability
Small Molecule (μM)
d
Temp. °C 25 50 52.1 55.4 59.4 64.9 69.2 72.1 74
DMSO
BTSA1.2
WB: BAX
17 KDa
WB: BAK
28 KDa
e
BTSA1.2, Tm = 66.5 ± 5 °C
DMSO, Tm=55 ± 0.8 °C
Relative BAX Signal
Temperature (°C)
f
Temp. °C 25 50 52.1 55.4 59. 64.9 69.2 72.1 74
g
BTSA1.2, Tm = 65 ± 0.7 °C
DMSO, Tm= 60.5 ± 0.6 °C
Relative BAX Signal
Temperature (°C)

Supplementary Figure 1. BTSA1.2, analog of BTSA1, with improved binding to BAX, cellular activity and on target engagement activity.

a, Structures of BTSA1 and BTSA1.2. **b**, Competitive fluorescence polarization assay of BTSA1 and BTSA1.2 competing FITC-BIM SAHB binding to BAX. Data are mean ± SD from n=3 independent experiments. **c**, Viability assay of lymphoma cell lines upon treatment of BTSA1 or BTSA1.2. Data are mean ± SD of three technical replicates from n=2 independent experiments. **d**, Cellular thermal shift assay (CETSA) of BAX and BAK in BxPC-3 cells treated with vehicle (DMSO) or 40 μM BTSA1.2 for 15 min. Blots are representative of three independent experiments. **e**, Melting curves of CETSA data for BAX in BxPC-3 cells were generated by densitometric analysis. Data are mean ± SD from n=3 independent experiments. Tm ± SEM values for DMSO and BTSA1.2 are indicated. **f**, Cellular thermal shift assay (CETSA) of BAX and BAK in SW480 cells treated with vehicle (DMSO) or 40 μM BTSA1.2 for 15 min. Blots are representative of three independent experiments. **g**, Melting curves of CETSA data for BAX in SW480 cells were generated by densitometric analysis. Data are mean ± SD from n=3 independent experiments. Tm ± SEM values for DMSO and BTSA1.2 are indicated. Source data are provided as a Source Data file.

a

Time (h)	1	2	3	Mean	SD
0.25	761	233	368	454	274
0.5	1740	474	1330	1181	646
1.0	4290	1580	2010	2627	1456
2.0	5260	2510	3240	3670	1425
4.0	3700	2540	3260	3167	586
8.0	2480	2120	1950	2183	271
24	1040	1100	1170	1103	65.1

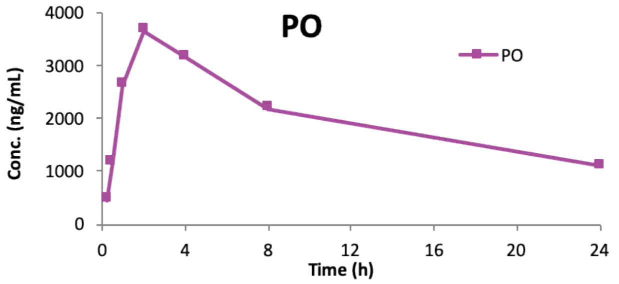

b

Time (h)	4	5	6	Mean	SD
0.083	2160	1870	3420	2483	824
0.25	1750	1440	1930	1707	248
0.5	1390	1560	2160	1703	405
1.0	1840	1650	2820	2103	628
2.0	2020	3180	2620	2607	580
4.0	1170	2520	2310	2000	726
8.0	899	1540	1050	1163	335
24	566	1090	1220	959	346

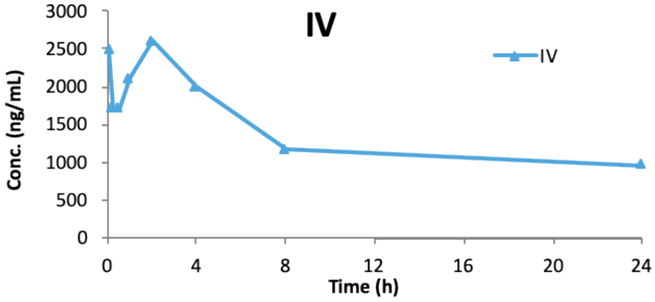

c

		$T_{1/2}$ (h)	T_{max} (h)	C_{max} (ng/mL)	AUC_{last} (h*ng/mL)	AUC_{INF_obs} (h*ng/mL)	CL_obs (mL/min/kg)	MRT_{INF_obs} (h)	Vss_obs (mL/kg)	F(%)
PO	Mean	14.405	3.333	3686.667	47003.131	70044.402	--	21.683	--	49.2
	SD	2.714	1.155	1409.302	6462.939	1551.231	--	4.433	--	
IV	Mean	24.640	2.000	2673.000	31866.471	67659.200	0.282	35.729	555.803	
	SD	8.513	0.000	593.745	8426.532	28220.741	0.131	12.900	155.903	

d

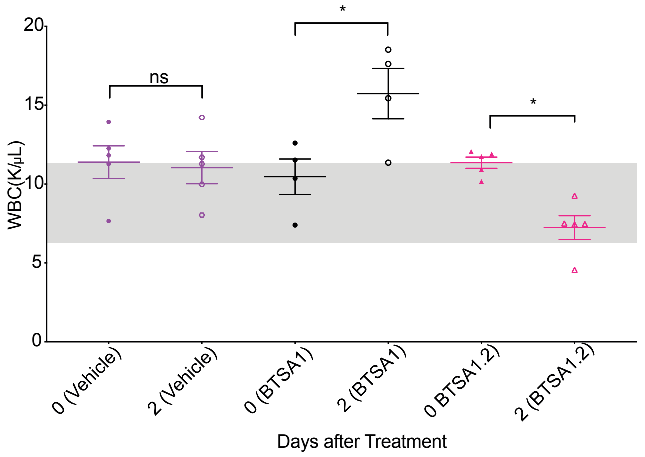

e

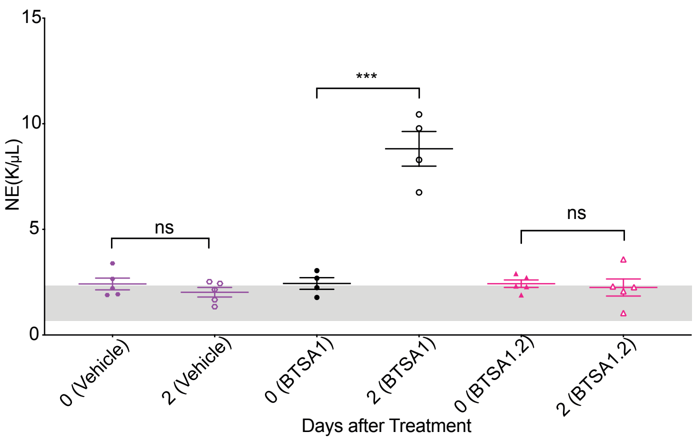

Supplementary Figure 2. Pharmacokinetic analysis of BTSA1.2 and comparison of *in vivo* safety between BTSA1.2 and BTSA1 by oral administration.

a, Concentrations (ng/mL) of BTSA1.2 in mice plasma after p.o. administration of 3 mg/kg BTSA1.2. **b**, Concentrations (ng/mL) of BTSA1.2 in mice plasma after i.v. administration of 1 mg/kg BTSA1.2. **c**, Pharmacokinetics parameters of BTSA1.2 after p.o. and i.v. administration in mice. Data are mean ± SD from n=3 mice. Counts of peripheral **d**, white blood cells and **e**, neutrophils in CD1-IGS mice treated with vehicle, 200 mg/kg BTSA1, or 200 mg/kg BTSA1.2 at 0 and 2 days after treatment. Compounds were administered orally. Normal blood counts range for CD-IGS male mice are indicated in gray. Data are mean ± SD from n=5 mice. Statistics were obtained using student t-test: *, $p<0.05$; **, $p<0.01$; ***, $p<0.001$; ****, $p<0.0001$. Source data are provided as a Source Data file.

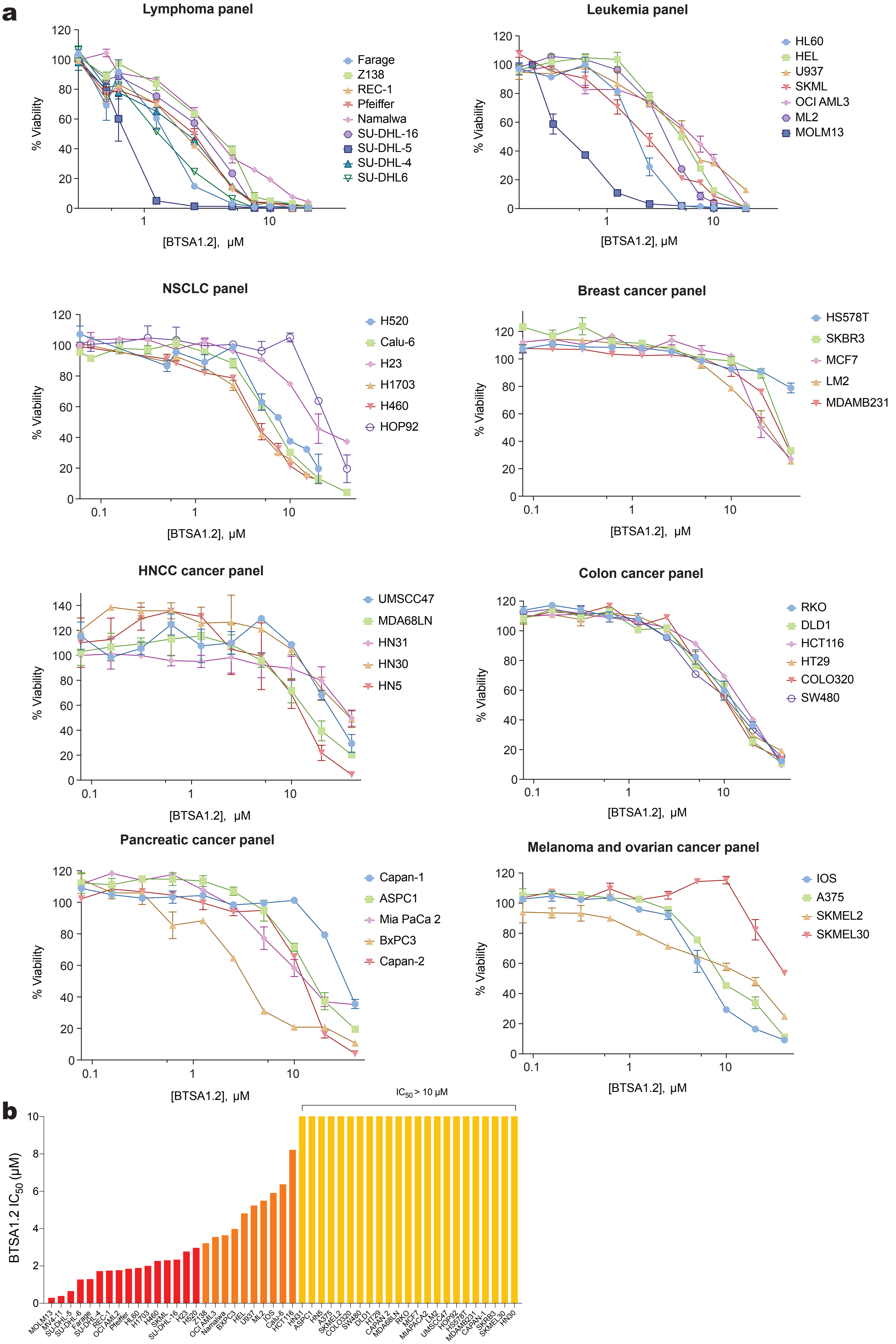
a
Lymphoma panel
% Viability
[BTSA1.2], μM
Farage
Z138
REC-1
Pfeiffer
Namalwa
SU-DHL-16
SU-DHL-5
SU-DHL-4
SU-DHL6
Leukemia panel
HL60
HEL
U937
SKML
OCI AML3
ML2
MOLM13
NSCLC panel
H520
Calu-6
H23
H1703
H460
HOP92
Breast cancer panel
HS578T
SKBR3
MCF7
LM2
MDAMB231
HNCC cancer panel
UMSCC47
MDA68LN
HN31
HN30
HN5
Colon cancer panel
RKO
DLD1
HCT116
HT29
COLO320
SW480
Pancreatic cancer panel
Capan-1
ASPC1
Mia PaCa 2
BxPC3
Capan-2
Melanoma and ovarian cancer panel
IOS
A375
SKMEL2
SKMEL30
b
BTSA1.2 IC50 (μM)
IC50 > 10 μM
MOLM13
MV4-11
SU-DHL-5
SU-DHL-6
Farage
SU-DHL-4
REC-1
OCI AML2
Pfeiffer
HL60
H1703
H460
SKML
SU-DHL-16
H23
H520
Z138
OCI AML3
Namalwa
BXPC3
HEL
U937
ML2
IOS
Calu-6
HCT116
HN31
ASPC1
HN5
A375
SKMEL2
COLO320
SW480
DLD1
HT29
CAPAN 2
MDA68LN
RKO
MCF7
MIAPACA2
LM2
UMSCC47
HOP92
HS578T
MDAMB231
CAPAN-1
SKRB3
SKMEL30
HN30

Supplementary Figure 3. BTSA1.2 activity in a diverse collection of human cancer cell lines.

a, Cell viability curves of diverse cell lines upon treatment with BTSA1.2 for 72hrs.

b, Bar graph plot of the cell viability IC_{50} (μM) arranged by sensitivity, red IC_{50}<3 μM; orange 3<IC_{50}<10 μM; yellow IC_{50}>10 μM. Data are mean ± SD of three technical replicates from n=2 independent experiments. Source data are provided as a Source Data file.

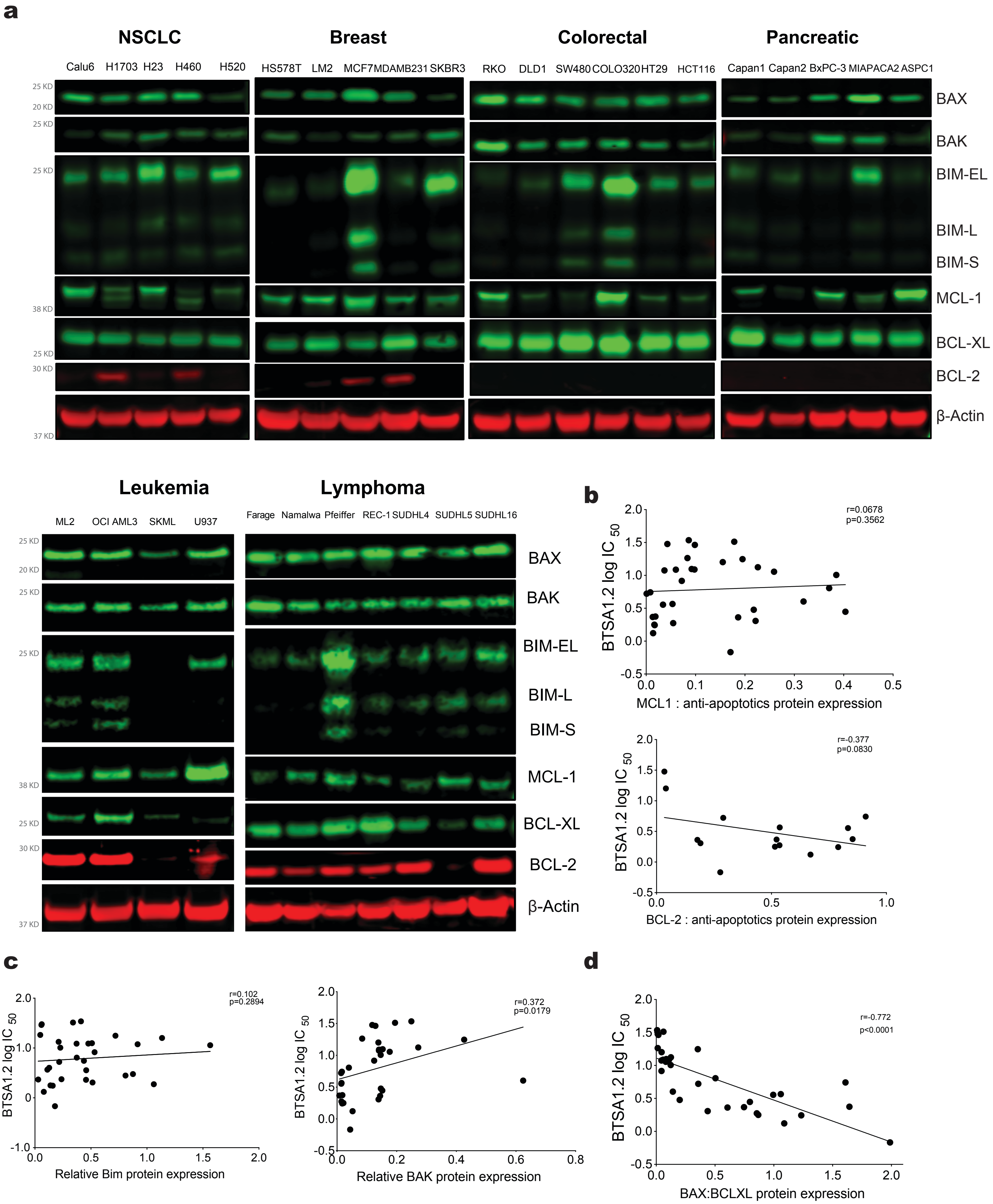
a
NSCLC
Calu6
H1703
H23
H460
H520
Breast
HS578T
LM2
MCF7
MDAMB231
SKBR3
Colorectal
RKO
DLD1
SW480
COLO320
HT29
HCT116
Pancreatic
Capan1
Capan2
BxPC-3
MIAPACA2
ASPC1
25 KD
20 KD
38 KD
30 KD
37 KD
BAX
BAK
BIM-EL
BIM-L
BIM-S
MCL-1
BCL-XL
BCL-2
β-Actin
Leukemia
ML2
OCI AML3
SKML
U937
Lymphoma
Farage
Namalwa
Pfeiffer
REC-1
SUDHL4
SUDHL5
SUDHL16
b
r=0.0678
p=0.3562
BTSA1.2 log IC 50
MCL1 : anti-apoptotics protein expression
r=-0.377
p=0.0830
BCL-2 : anti-apoptotics protein expression
c
r=0.102
p=0.2894
Relative Bim protein expression
r=0.372
p=0.0179
Relative BAK protein expression
d
r=-0.772
p<0.0001
BAX:BCLXL protein expression

Supplementary Figure 4. BCL-2 family protein expression levels in solid tumor and hematological cancer cell lines and correlation analysis of BTSA1.2 activity.

a, Protein expression levels of key BCL-2 family members detected by Licor. β-Actin was used as loading control. **b-d**, Correlation of sensitivity to BTSA1.2 with **b**, MCL-1, BCL-2, **c**, BIM, BAK and **d**, BAX:BCL-XL relative protein levels using Pearson-Correlation. Relative protein levels were first normalized to β-Actin loading control, p value was calculated using two-tailed student t-test. Data are representative of at least n=2 independent experiments. Source data are provided as a Source Data file.

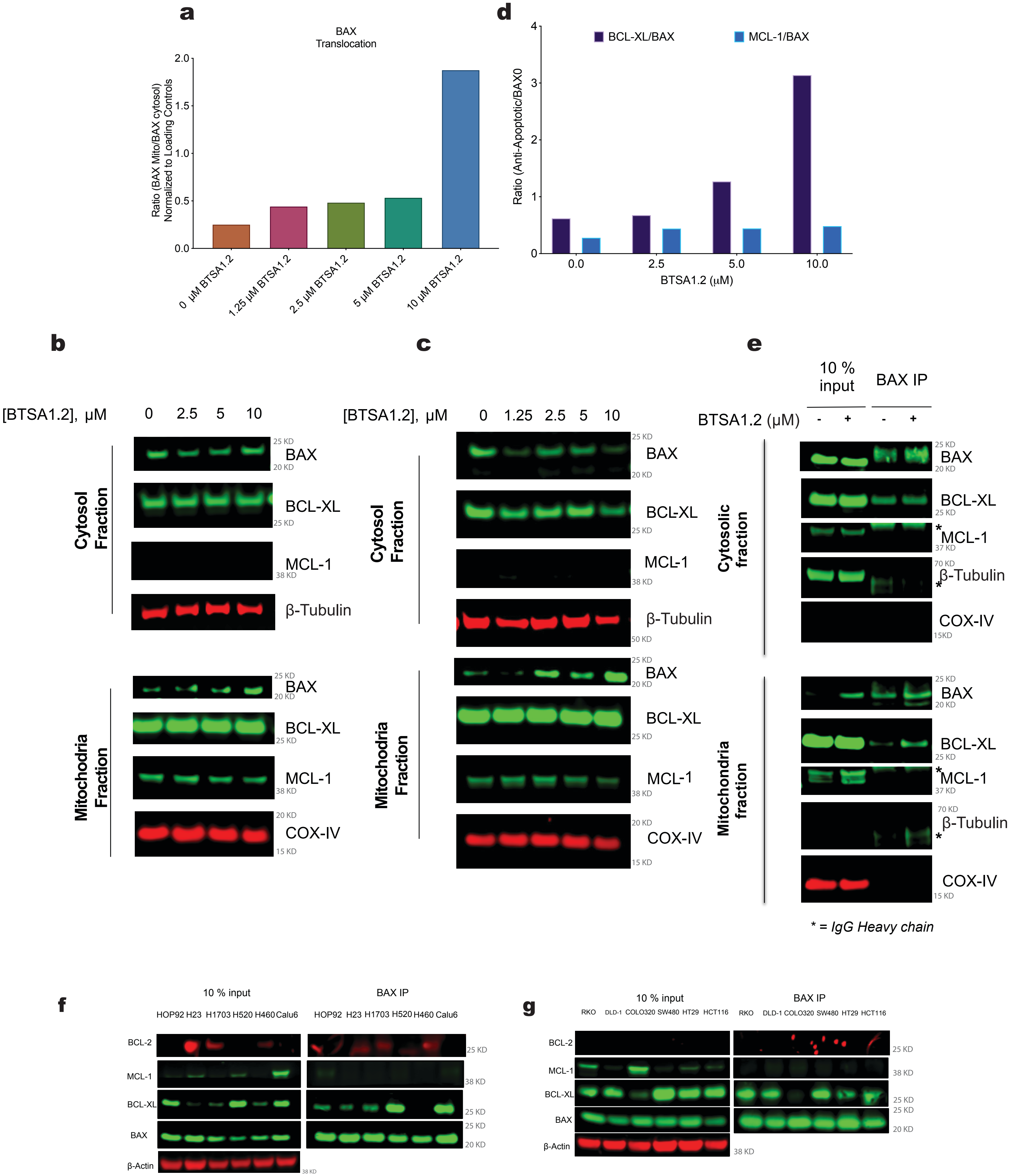

a
BAX Translocation
Ratio (BAX Mito/BAX cytosol) Normalized to Loading Controls
2.0
1.5
1.0
0.5
0.0
0 μM BTSA1.2
1.25 μM BTSA1.2
2.5 μM BTSA1.2
5 μM BTSA1.2
10 μM BTSA1.2
d
BCL-XL/BAX
MCL-1/BAX
Ratio (Anti-Apoptotic/BAX0
4
3
2
1
0
0.0
2.5
5.0
10.0
BTSA1.2 (μM)
b
[BTSA1.2], μM
0
2.5
5
10
Cytosol Fraction
BAX
BCL-XL
MCL-1
β-Tubulin
Mitochondria Fraction
BAX
BCL-XL
MCL-1
COX-IV
25 KD
20 KD
38 KD
15 KD
c
[BTSA1.2], μM
0
1.25
2.5
5
10
Cytosol Fraction
BAX
BCL-XL
MCL-1
β-Tubulin
Mitochondria Fraction
BAX
BCL-XL
MCL-1
COX-IV
50 KD
e
10 % input
BAX IP
BTSA1.2 (μM)
-
+
Cytosolic fraction
BAX
BCL-XL
MCL-1
β-Tubulin
COX-IV
Mitochondria fraction
BAX
BCL-XL
MCL-1
β-Tubulin
COX-IV
37 KD
70 KD
15KD
* = IgG Heavy chain
f
10 % input
BAX IP
HOP92 H23 H1703 H520 H460 Calu6
BCL-2
MCL-1
BCL-XL
BAX
β-Actin
g
10 % input
BAX IP
RKO DLD-1 COLO320 SW480 HT29 HCT116
BCL-2
MCL-1
BCL-XL
BAX
β-Actin

Supplementary Figure 5. BCL-XL regulates BAX activation by BTSA1.2

a, Quantification of translocated BAX upon 4 hrs treatment with BTSA1.2 in BxPC-3 cell line (related to Fig. 1c). **b**, BAX translocation upon 4 hrs treatment with BTSA1.2 in SW480 cell line. **c**, BAX translocation upon 18 hrs treatment with BTSA1.2 in BxPC-3 cell line. **d**, Quantification of co-immunoprecipitated BAX with anti-apoptotic BCL-XL and MCL-1 upon 4 hrs treatment with BTSA1.2 in BxPC-3 cell line (related to Fig. 1d). **e**, Mitochondrial and cytosolic BAX co-IP upon 4 hrs treatment with 10 μM BTSA1.2 in BxPC-3 cell line. Data are representative of at least n=3 independent experiments. Western blot analysis of BAX Co-IP in a panel of **f**, NSCLC and **g**, colorectal cells. Data are representative of n=3 independent experiments. Source data are provided as a Source Data file.

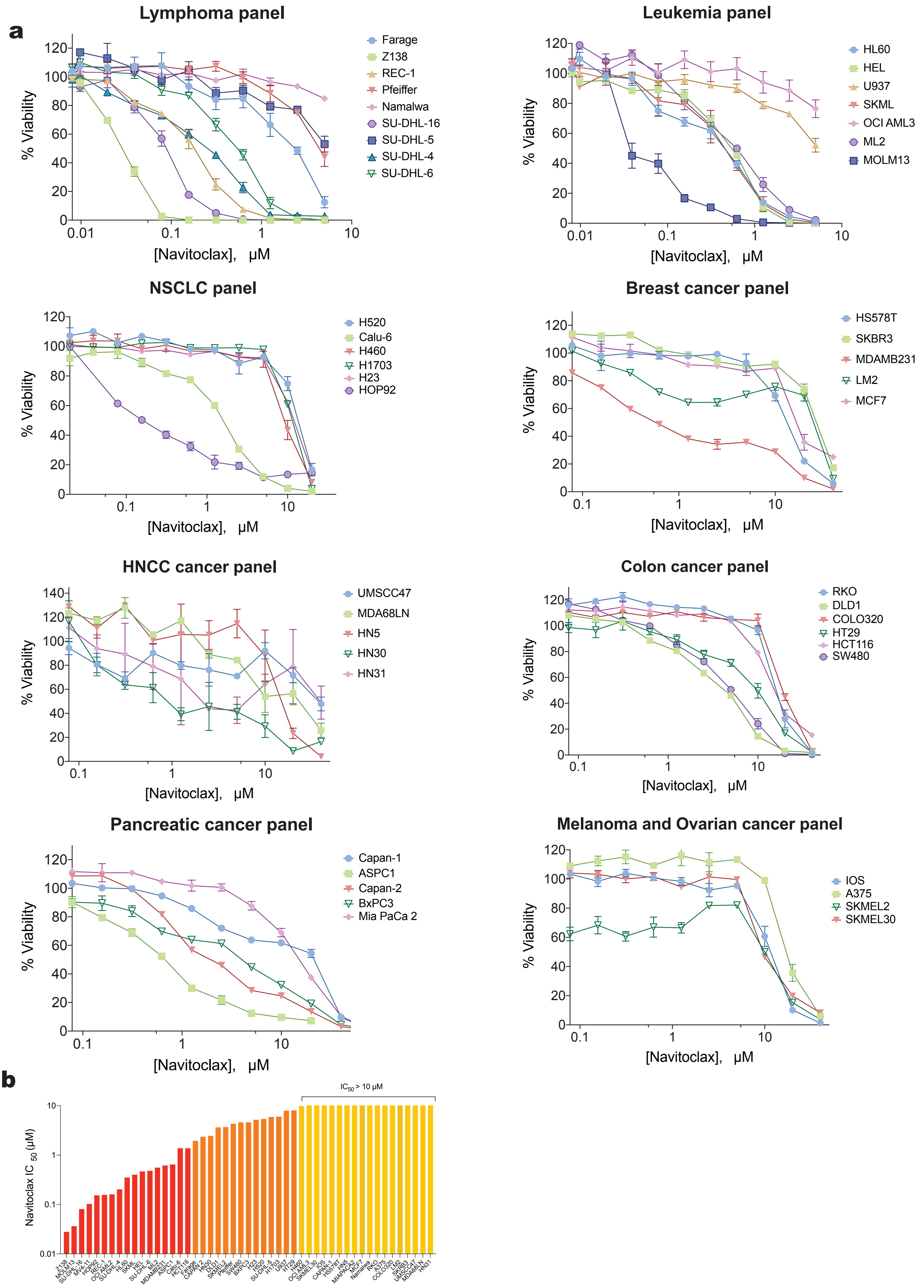
a
Lymphoma panel
% Viability
[Navitoclax], µM
Farage
Z138
REC-1
Pfeiffer
Namalwa
SU-DHL-16
SU-DHL-5
SU-DHL-4
SU-DHL-6
Leukemia panel
HL60
HEL
U937
SKML
OCI AML3
ML2
MOLM13
NSCLC panel
H520
Calu-6
H460
H1703
H23
HOP92
Breast cancer panel
HS578T
SKBR3
MDAMB231
LM2
MCF7
HNCC cancer panel
UMSCC47
MDA68LN
HN5
HN30
HN31
Colon cancer panel
RKO
DLD1
COLO320
HT29
HCT116
SW480
Pancreatic cancer panel
Capan-1
ASPC1
Capan-2
BxPC3
Mia PaCa 2
Melanoma and Ovarian cancer panel
IOS
A375
SKMEL2
SKMEL30
b
IC50 > 10 µM
Navitoclax IC 50 (µM)

c

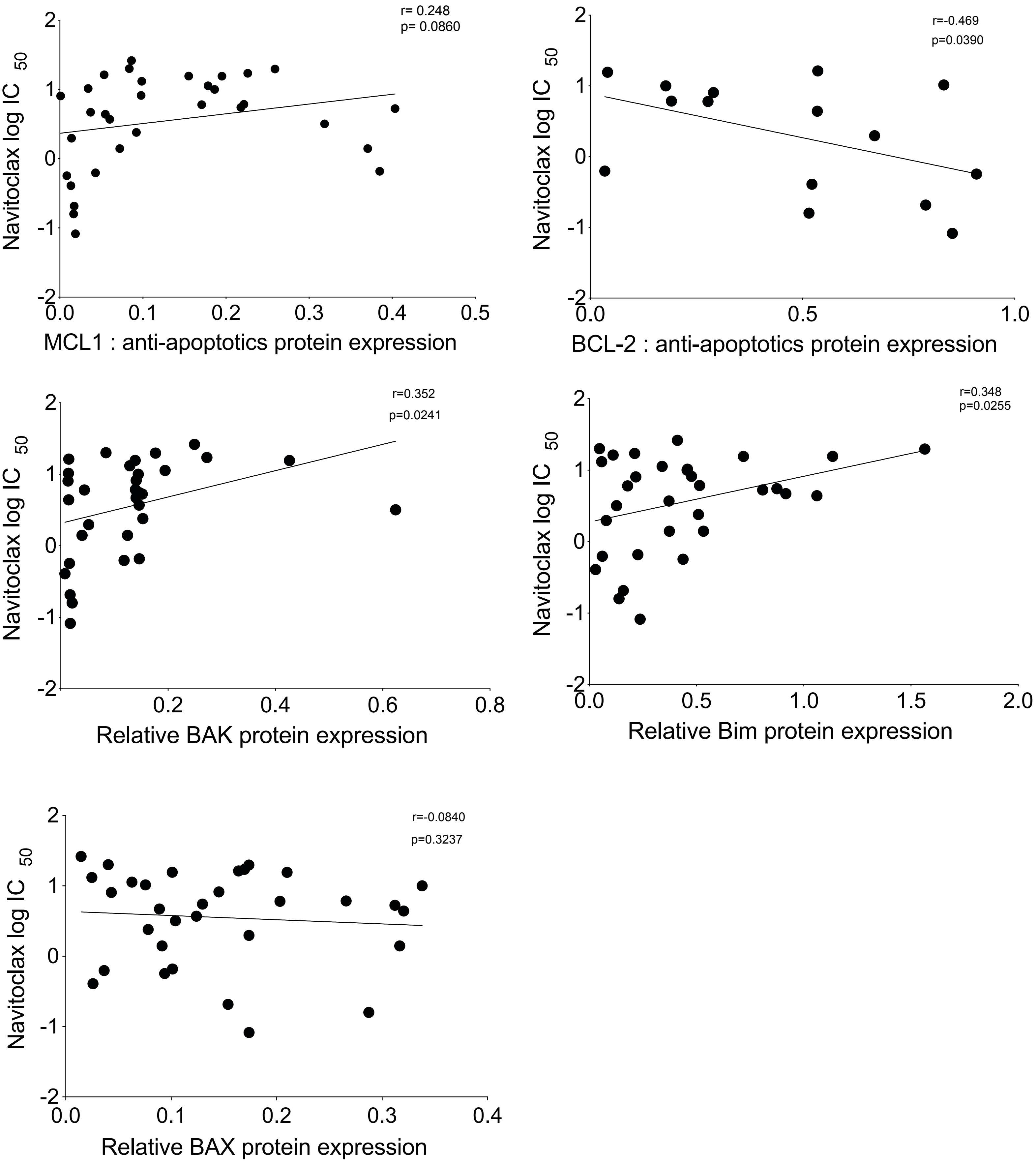
r= 0.248
p= 0.0860
Navitoclax log IC 50
MCL1 : anti-apoptotics protein expression
r=-0.469
p=0.0390
Navitoclax log IC 50
BCL-2 : anti-apoptotics protein expression
r=0.352
p=0.0241
Navitoclax log IC 50
Relative BAK protein expression
r=0.348
p=0.0255
Navitoclax log IC 50
Relative Bim protein expression
r=-0.0840
p=0.3237
Navitoclax log IC 50
Relative BAX protein expression

Supplementary Figure 6. Navitoclax activity in a diverse collection of human cancer cell lines and correlation analysis of Navitoclax activity.

a, Cell viability curves of cell lines upon treatment with Navitoclax for 72 hrs. Data are mean ± SD of three technical replicates from n=2 independent experiments. **b**, Bar graph plot of the cell viability IC_{50} (µM) arranged by sensitivity, red IC_{50}<1.5 µM; orange 1.5<IC_{50}<10 µM; yellow IC_{50}>10 µM. Correlation of sensitivity to Navitoclax with MCL-1, BCL-2, BIM, BAK and BAX relative protein levels using Pearson-Correlation. **c**, Relative protein levels were first normalized to β-Actin loading control, p value was calculated using student t-test. Source data are provided as a Source Data file.

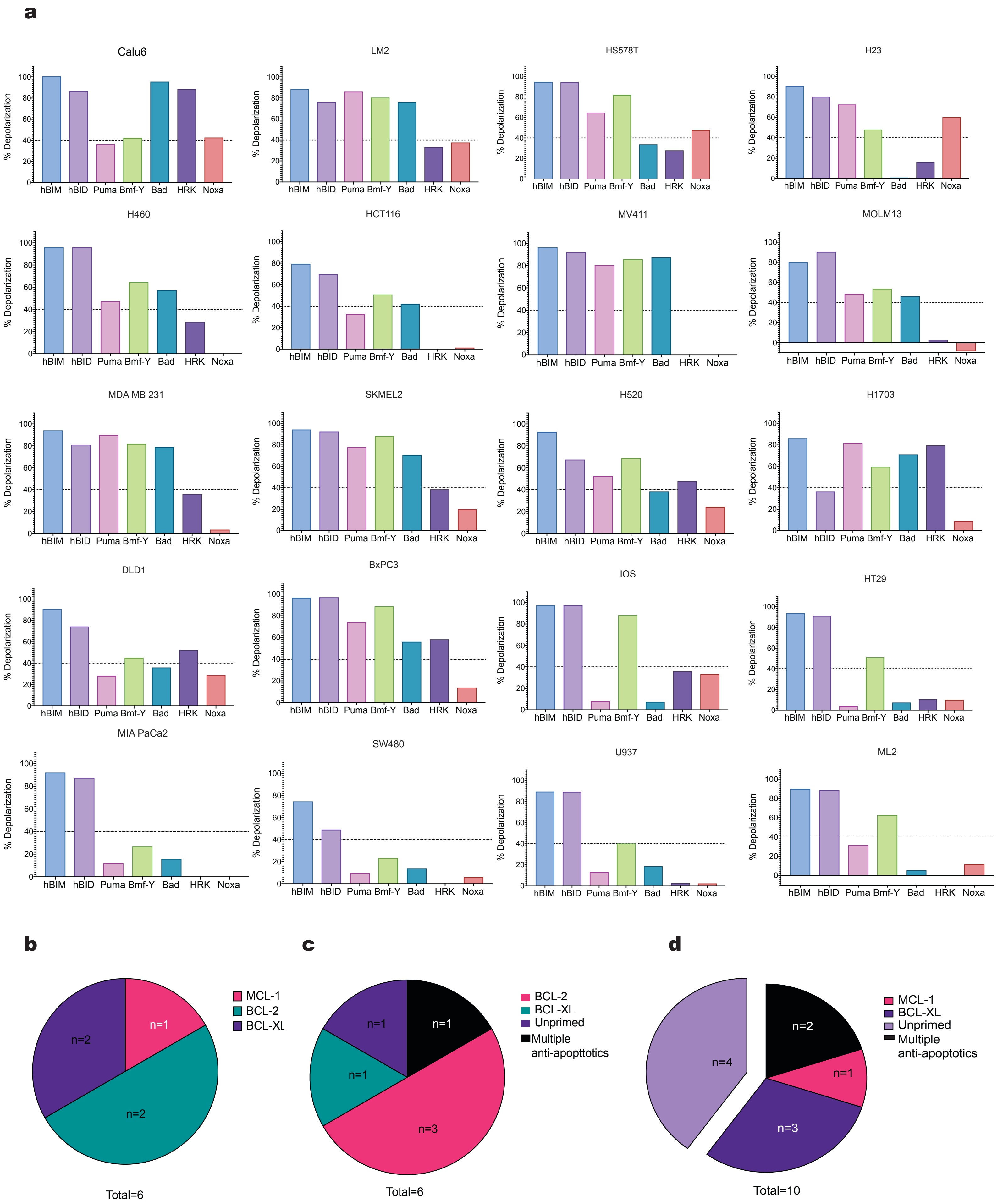

a
Calu6
LM2
HS578T
H23
H460
HCT116
MV411
MOLM13
MDA MB 231
SKMEL2
H520
H1703
DLD1
BxPC3
IOS
HT29
MIA PaCa2
SW480
U937
ML2
% Depolarization
0
20
40
60
80
100
hBIM
hBID
Puma
Bmf-Y
Bad
HRK
Noxa
b
MCL-1
BCL-2
BCL-XL
n=1
n=2
n=2
Total=6
c
BCL-2
BCL-XL
Unprimed
Multiple anti-apopttotics
n=1
n=1
n=1
n=3
Total=6
d
MCL-1
BCL-XL
Unprimed
Multiple anti-apoptotics
n=2
n=1
n=3
n=4
Total=10

Supplementary Figure 7. BH3-Profiling of solid tumor and hematological cancer cell lines.

a, % Mitochondria depolarization upon treatment with BH3-only derived peptides. **b**, BH3 profiling predicts apoptotic blocks correlated with BTSA1.2 sensitivity. Data are mean from n=3 replicates **c**, BH3-profiling predicts apoptotic blocks correlated with Navitoclax sensitivity. **d**, BH3-profiling predicts apoptotic blocks correlated with BTSA1.2 and Navitoclax resistance. Source data are provided as a Source Data file.

a

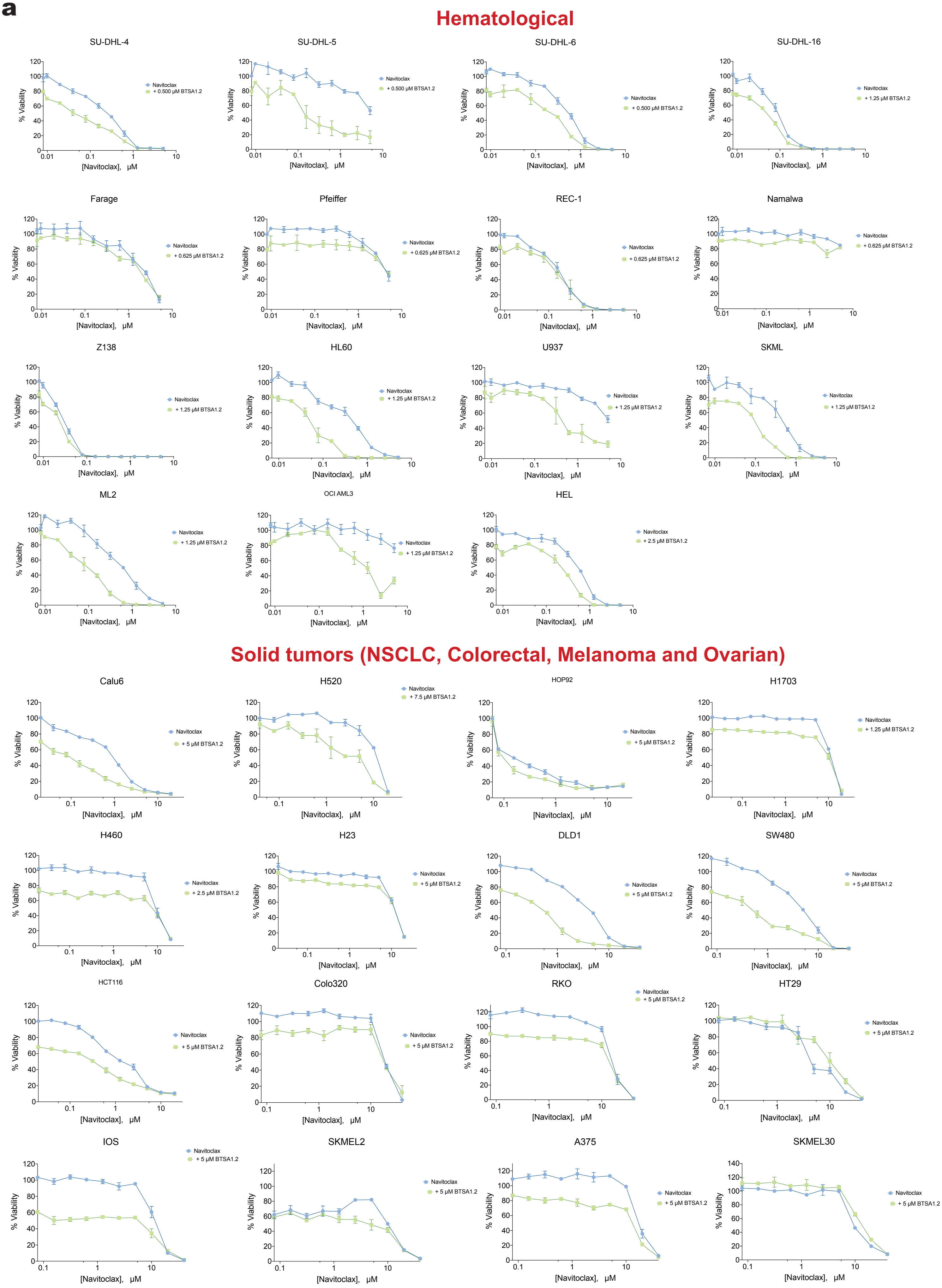
Hematological
SU-DHL-4
Navitoclax
+ 0.500 µM BTSA1.2
SU-DHL-5
Navitoclax
+ 0.500 µM BTSA1.2
SU-DHL-6
Navitoclax
+ 0.500 µM BTSA1.2
SU-DHL-16
Navitoclax
+ 1.25 µM BTSA1.2
Farage
Navitoclax
+ 0.625 µM BTSA1.2
Pfeiffer
Navitoclax
+ 0.625 µM BTSA1.2
REC-1
Navitoclax
+ 0.625 µM BTSA1.2
Namalwa
Navitoclax
+ 0.625 µM BTSA1.2
Z138
Navitoclax
+ 1.25 µM BTSA1.2
HL60
Navitoclax
+ 1.25 µM BTSA1.2
U937
Navitoclax
+ 1.25 µM BTSA1.2
SKML
Navitoclax
+ 1.25 µM BTSA1.2
ML2
Navitoclax
+ 1.25 µM BTSA1.2
OCI AML3
Navitoclax
+ 1.25 µM BTSA1.2
HEL
Navitoclax
+ 2.5 µM BTSA1.2
Solid tumors (NSCLC, Colorectal, Melanoma and Ovarian)
Calu6
Navitoclax
+ 5 µM BTSA1.2
H520
Navitoclax
+ 7.5 µM BTSA1.2
HOP92
Navitoclax
+ 5 µM BTSA1.2
H1703
Navitoclax
+ 1.25 µM BTSA1.2
H460
Navitoclax
+ 2.5 µM BTSA1.2
H23
Navitoclax
+ 5 µM BTSA1.2
DLD1
Navitoclax
+ 5 µM BTSA1.2
SW480
Navitoclax
+ 5 µM BTSA1.2
HCT116
Navitoclax
+ 5 µM BTSA1.2
Colo320
Navitoclax
+ 5 µM BTSA1.2
RKO
Navitoclax
+ 5 µM BTSA1.2
HT29
Navitoclax
+ 5 µM BTSA1.2
IOS
Navitoclax
+ 5 µM BTSA1.2
SKMEL2
Navitoclax
+ 5 µM BTSA1.2
A375
Navitoclax
+ 5 µM BTSA1.2
SKMEL30
Navitoclax
+ 5 µM BTSA1.2
% Viability
[Navitoclax], µM

Solid tumors (Pancreatic, Breast, HNCC)

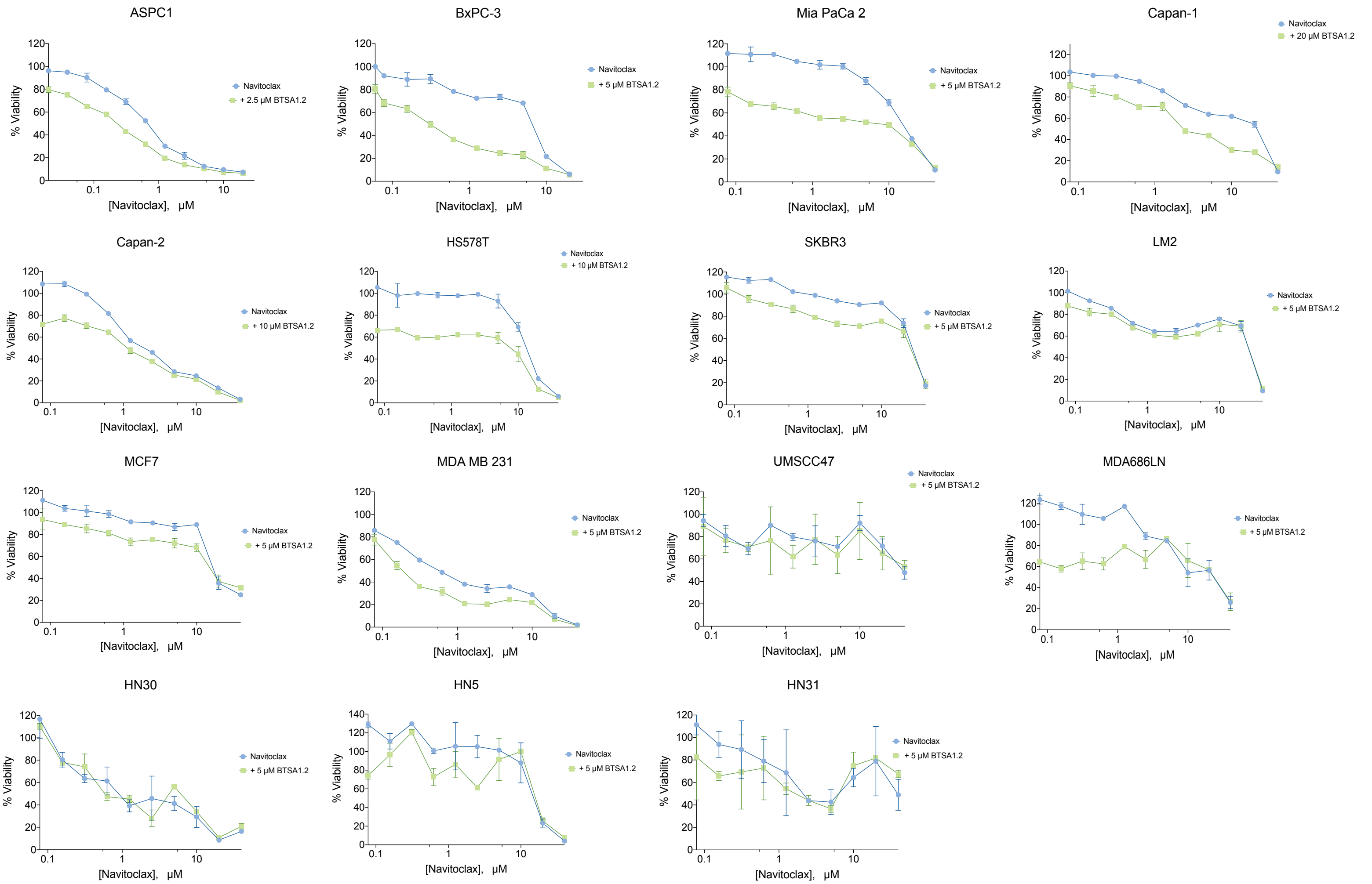

Supplementary Figure 8. BTSA1.2 and Navitoclax combination to inhibit cell viability in resistant tumor cell lines.

a, Viability curves of cell lines treated with Navitoclax in combination with a constant sensitizing concentration (induce <20% viability loss) of BTSA1.2 (corresponding to Fig. 2a,b). Data are mean ± SD of three technical replicates from n=2 independent experiments. Source data are provided as a Source Data file.

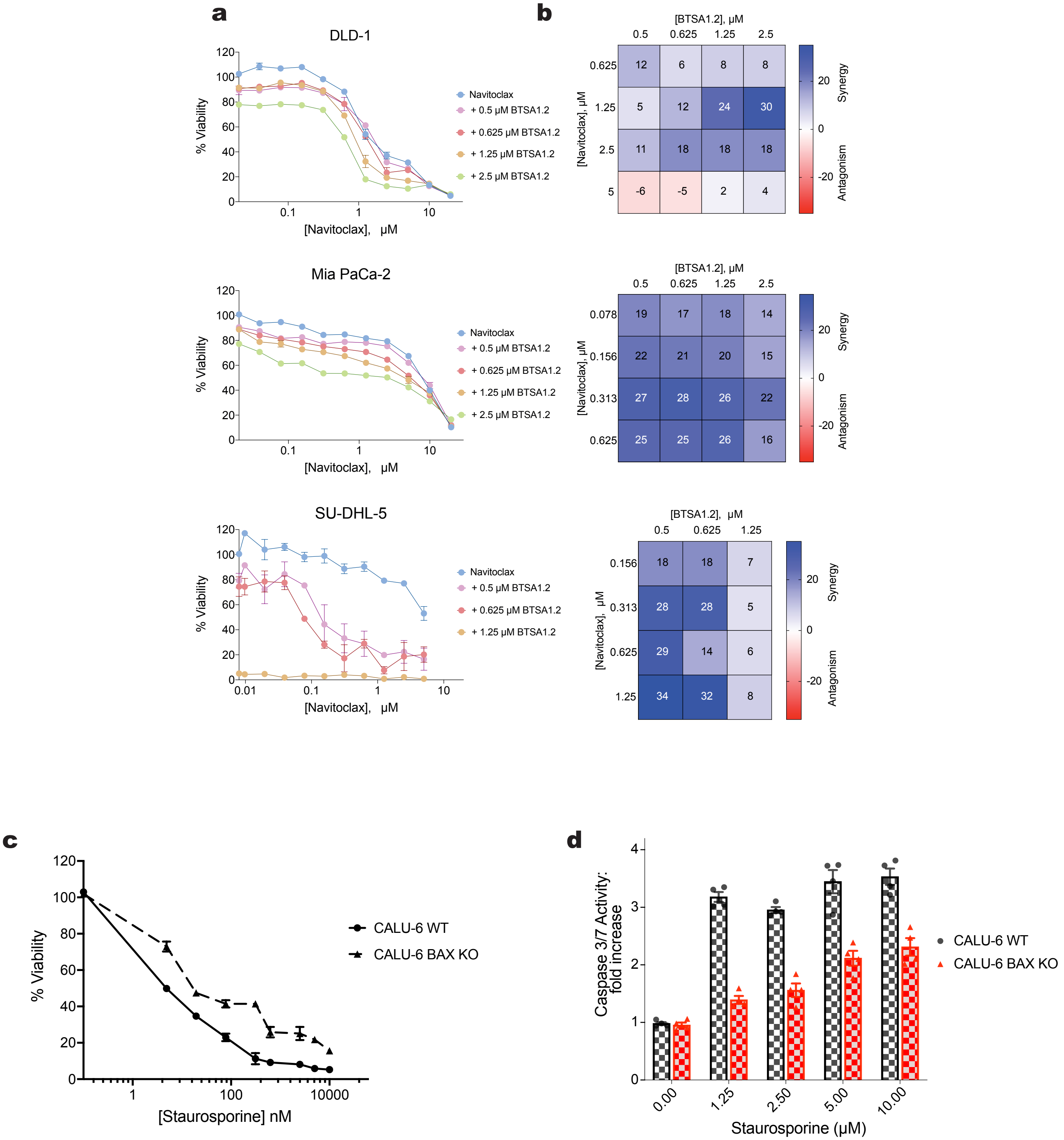
a
DLD-1
% Viability
[Navitoclax], μM
Navitoclax
+ 0.5 μM BTSA1.2
+ 0.625 μM BTSA1.2
+ 1.25 μM BTSA1.2
+ 2.5 μM BTSA1.2
Mia PaCa-2
SU-DHL-5
b
[BTSA1.2], μM
[Navitoclax], μM
0.5 0.625 1.25 2.5
0.625 12 6 8 8
1.25 5 12 24 30
2.5 11 18 18 18
5 -6 -5 2 4
0.5 0.625 1.25 2.5
0.078 19 17 18 14
0.156 22 21 20 15
0.313 27 28 26 22
0.625 25 25 26 16
0.5 0.625 1.25
0.156 18 18 7
0.313 28 28 5
0.625 29 14 6
1.25 34 32 8
Synergy
Antagonism
c
% Viability
[Staurosporine] nM
CALU-6 WT
CALU-6 BAX KO
d
Caspase 3/7 Activity:
fold increase
Staurosporine (μM)
0.00
1.25
2.50
5.00
10.00
CALU-6 WT
CALU-6 BAX KO

Supplementary Figure 9. BTSA1.2 and Navitoclax synergize to inhibit cell viability in resistant tumor cell lines.

a, Dose-response curves of Navitoclax in the presence of various doses of BTSA1.2 in a panel of resistant cancer cell lines to single agents from various tissue types (Colon=DLD1, Pancreatic=Mia PaCa-2, Lymphoma=SU-DHL-5). Effects on cell viability were measured by CellTiter-Glo after 72 hrs of treatment. **b**, Bliss synergy score heat map from combinatorial treatment of BTSA1.2 and Navitoclax in different cancer tissue types. Data are mean ± SD of three technical replicates from n=3 independent experiments. **c,** Cell viability at 72 hrs in WT and CRISPR/Cas9 BAX KO Calu-6 cell lines treated with various doses of Staurosporine. **d**, Caspase 3/7 activity in WT and CRISPR/Cas9 BAX KO Calu-6 cell lines at 24 hrs treatment with various doses of Staurosporine. Data are mean ± SD of four technical replicates from n=3 independent experiments. Source data are provided as a Source Data file.

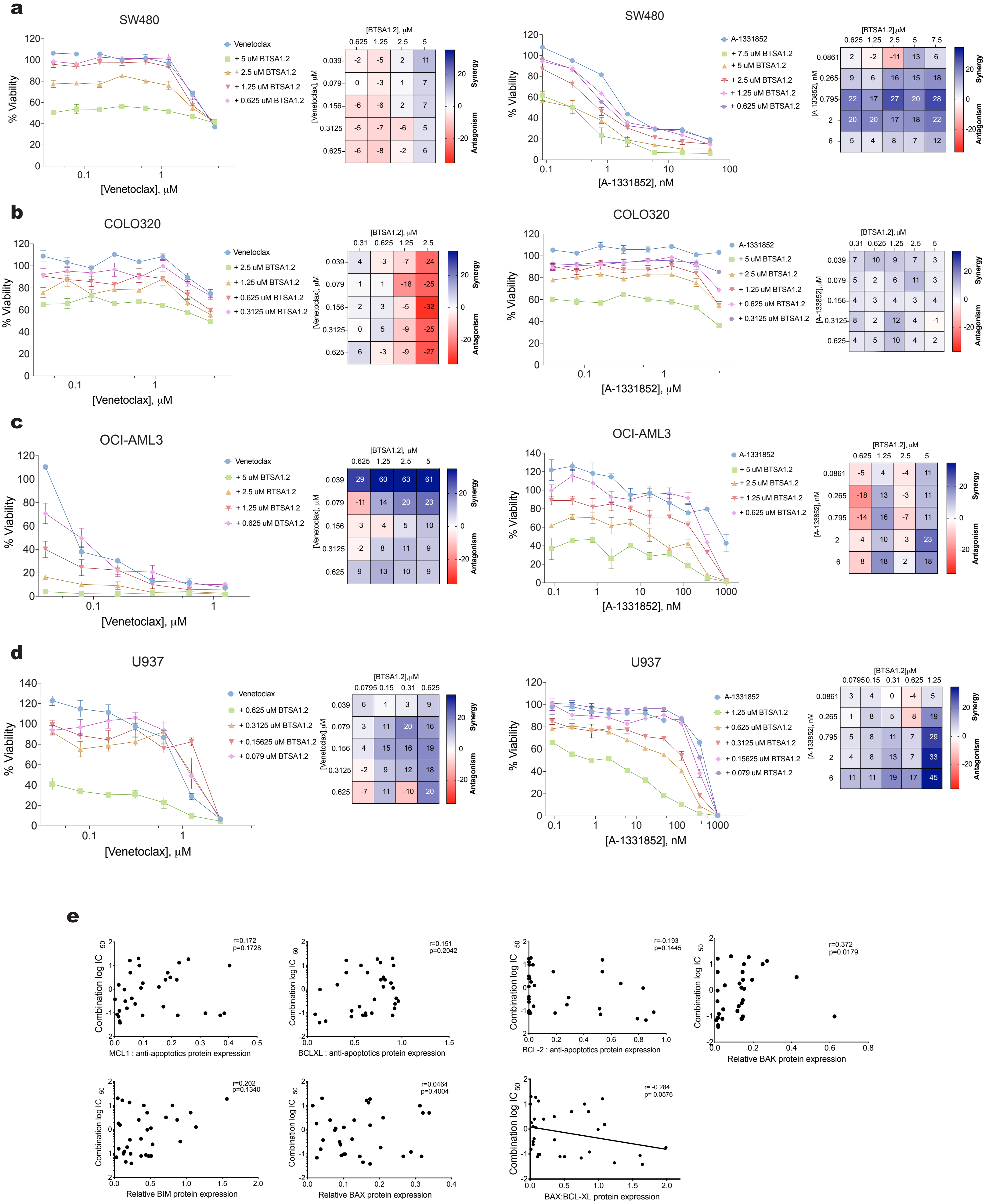
a
SW480
Venetoclax
+ 5 uM BTSA1.2
+ 2.5 uM BTSA1.2
+ 1.25 uM BTSA1.2
+ 0.625 uM BTSA1.2
% Viability
[Venetoclax], µM
[BTSA1.2], µM
Synergy
Antagonism
SW480
A-1331852
+ 7.5 uM BTSA1.2
+ 5 uM BTSA1.2
+ 2.5 uM BTSA1.2
+ 1.25 uM BTSA1.2
+ 0.625 uM BTSA1.2
[A-1331852], nM
b
COLO320
+ 0.3125 uM BTSA1.2
[A-1331852], µM
c
OCI-AML3
d
U937
+ 0.15625 uM BTSA1.2
+ 0.079 uM BTSA1.2
e
r=0.172
p=0.1728
MCL1 : anti-apoptotics protein expression
Combination log IC50
r=0.151
p=0.2042
BCLXL : anti-apoptotics protein expression
r=-0.193
p=0.1445
BCL-2 : anti-apoptotics protein expression
r=0.372
p=0.0179
Relative BAK protein expression
r=0.202
p=0.1340
Relative BIM protein expression
r=0.0464
p=0.4004
Relative BAX protein expression
r= -0.284
p= 0.0576
BAX:BCL-XL protein expression

Supplementary Figure 10. BTSA1.2 and BCL-XL selective inhibitor A1331852 synergize to inhibit cell viability in sensitive tumor cell lines to the Navitoclax/BTSA1.2 combination.

a,**b,** Dose-response curves of the BCL-XL selective inhibitor A1331852 or the BCL-2 selective inhibitor Venetoclax in the presence of various doses of BTSA1.2 in a sensitive (SW480) or resistant (COLO-320) cancer cell line to the Navitoclax/BTSA1.2 combination. Effects on cell viability were measured by CellTiter-Glo after 72 hrs of treatment. Bliss synergy score heat map from combinatorial treatment. Data are mean ± SD of three technical replicates from n=3 independent experiments. **c**,**d**, Dose-response curves of the BCL-XL selective inhibitor A1331852 or the BCL-2 selective inhibitor Venetoclax in the presence of various doses of BTSA1.2 in OCI-AML3 or U937 hematologic cell line sensitive to the Navitoclax/BTSA1.2 combination. Effects on cell viability were measured by CellTiter-Glo after 72 hrs of treatment. Bliss synergy score heat map from combinatorial treatment. Data are mean ± SD of three technical replicates from n=3 independent experiments. **e**, BCL-2 family protein levels compared with sensitivity to the BTSA1.2 and Navitoclax combination: Protein expression levels correlation with Combination. Correlation of sensitivity to BTSA1.2 and Navitoclax combination with MCL-1, BCL-XL BCL-2, BIM, BAK and BAX relative protein levels using Pearson-Correlation. Relative protein levels were first normalized to b-Actin loading control, p value was calculated using student t-test. Data are representative of at least n=2 independent experiments. Source data are provided as a Source Data file.

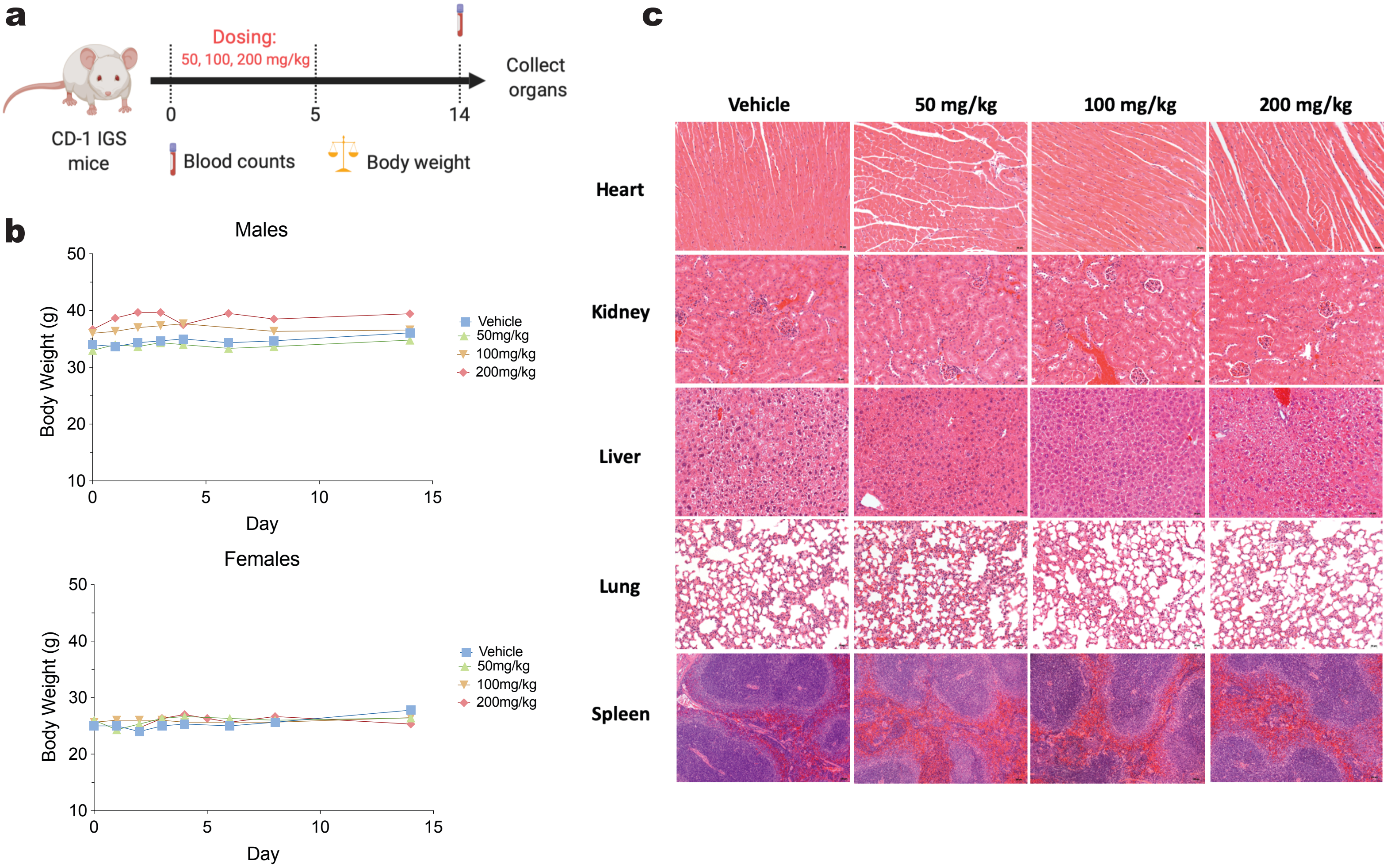

a
Dosing:
50, 100, 200 mg/kg
Collect organs
0
5
14
CD-1 IGS mice
Blood counts
Body weight
b
Males
Body Weight (g)
Day
Vehicle
50mg/kg
100mg/kg
200mg/kg
Females
Body Weight (g)
Day
Vehicle
50mg/kg
100mg/kg
200mg/kg
c
Vehicle
50 mg/kg
100 mg/kg
200 mg/kg
Heart
Kidney
Liver
Lung
Spleen

Supplementary Figure 11. Maximum tolerated dose analysis of BTSA1.2.

a, BTSA1.2 is well-tolerated *in vivo*: Schematic of MTD and toxicity study of BTSA1.2. CD-IGS female and male mice were treated daily with increasing concentration of BTSA1.2 orally administered for 5 days. Body weights and blood counts were measured at indicated days. Mice were sacrificed 14 days after the first treatment and organs were collected for pathology analysis. **b**, Body weight measurements of CD1-IGS mice after treatment with vehicle or BTSA1.2. Data in **a** and **b** are mean ± SD from n=6 mice. **c**, Representative tissue sections spleen, heart, liver, lungs and kidney using Hematoxylin and Eosin (H&E) staining from mice after treatment of vehicle or BTSA1.2. Scale bars, 100 μm. Data is representative of n=3 independent samples per group. Source data are provided as a Source Data file.

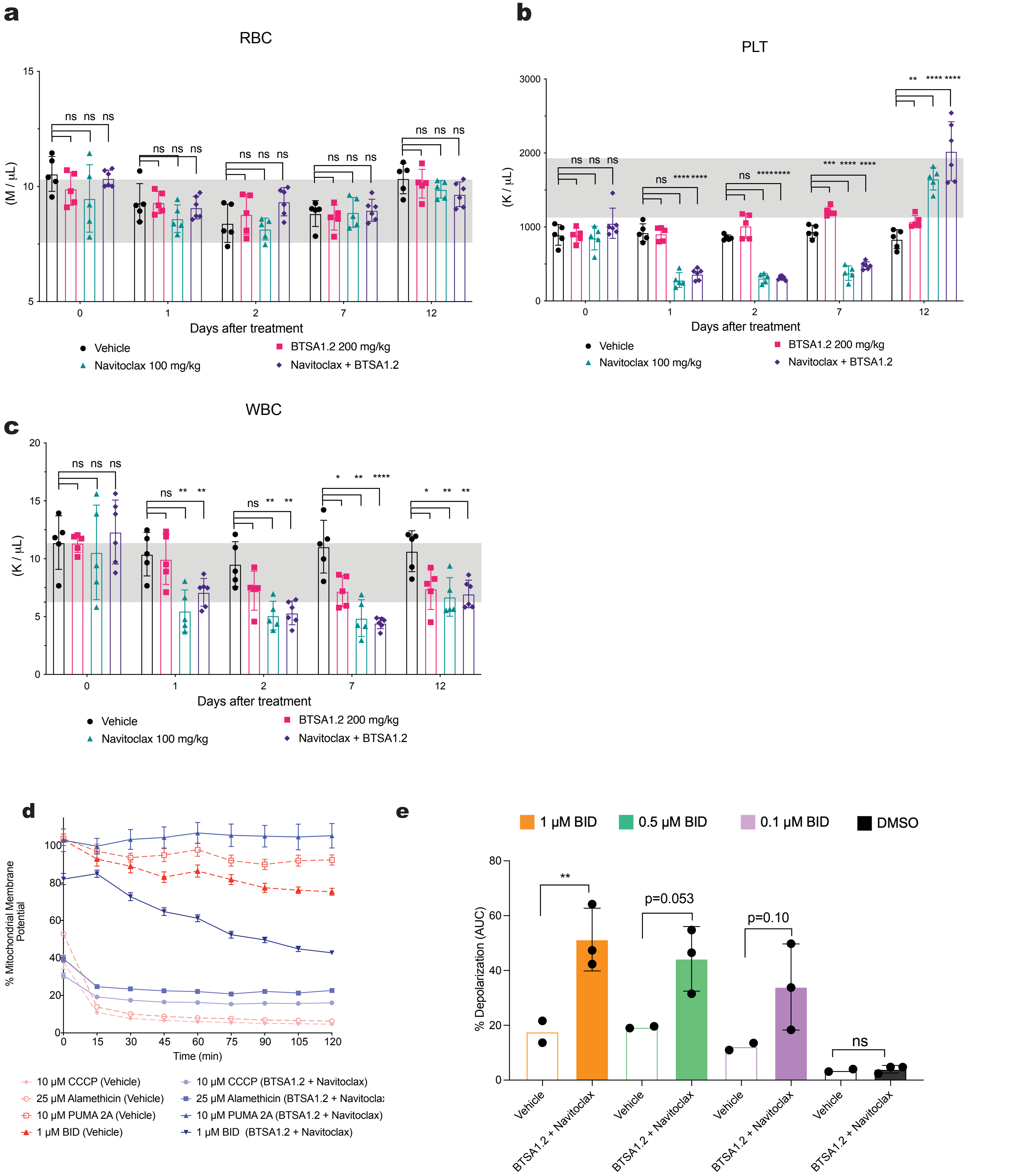
a
RBC
15
10
5
(M / µL)
ns ns ns
0
1
2
7
12
Days after treatment
Vehicle
BTSA1.2 200 mg/kg
Navitoclax 100 mg/kg
Navitoclax + BTSA1.2
b
PLT
3000
2000
1000
0
(K / µL)
ns ns ns
ns **** ****
ns ********
*** **** ****
** **** ****
Days after treatment
Vehicle
BTSA1.2 200 mg/kg
Navitoclax 100 mg/kg
Navitoclax + BTSA1.2
c
WBC
20
15
10
5
0
(K / µL)
ns ns ns
ns ** **
ns ** **
* ** ****
* ** **
Days after treatment
Vehicle
BTSA1.2 200 mg/kg
Navitoclax 100 mg/kg
Navitoclax + BTSA1.2
d
% Mitochondrial Membrane Potential
100
80
60
40
20
0
0 15 30 45 60 75 90 105 120
Time (min)
10 µM CCCP (Vehicle)
25 µM Alamethicin (Vehicle)
10 µM PUMA 2A (Vehicle)
1 µM BID (Vehicle)
10 µM CCCP (BTSA1.2 + Navitoclax)
25 µM Alamethicin (BTSA1.2 + Navitocla:
10 µM PUMA 2A (BTSA1.2 + Navitoclax)
1 µM BID (BTSA1.2 + Navitoclax)
e
1 µM BID
0.5 µM BID
0.1 µM BID
DMSO
% Depolarization (AUC)
80
60
40
20
0
**
p=0.053
p=0.10
ns
Vehicle
BTSA1.2 + Navitoclax
Vehicle
BTSA1.2 + Navitoclax
Vehicle
BTSA1.2 + Navitoclax
Vehicle
BTSA1.2 + Navitoclax

Supplementary Figure 12. Combinatorial therapy of BTSA1.2 and Navitoclax is well tolerated *in vivo*, does not enhance Navitoclax driven toxicity in the hematopoietic system and primes tumors *in vivo* to apoptosis.

a-c, Combinatorial therapy of BTSA1.2 and Navitoclax is well tolerated *in vivo*: Counts of peripheral **a**, red blood cells; **b**, platelets; and **c**, white blood cells in CD1-IGS mice treated with vehicle, 100 mg/kg Navitoclax, 200 mg/kg BTSA1.2 or the combination 0, 1, 2, 7 and 12 days after treatment. Data in **a-c** represents mean ± SD from n = 5 mice in Vehicle, BTSA1.2 and Navitoclax and n=6 mice in Combination groups. Statistics for these panels were obtained using one-way ANOVA: *, p<0.05; **, p<0.01; ***, p<0.001; ****, p<0.0001. **d,e**, Dynamic BH3 profiling of tumors from mice treated with vehicle or Navitoclax and BTSA1.2 combination: **d**, Example of kinetic curve of mitochondria potential in tumors treated with vehicle or combination upon stimuli of BH3-BID peptide, Puma2A, CCCP or Alamethicin. Data represent mean ± SD of three replicates from n=2 independent experiments. **e**, Bar graph represent % of mitochondria depolarization of tumor cells detected by JC-1 upon treatment BH3-BID derived peptide or DMSO. Data represent mean ± SD from n = 2 mice in Vehicle and n=3 mice in Combination groups. Statistics for this panel were obtained using one-way Anova: *, p<0.05; **, p<0.01; ***, p<0.001; ****, p<0.0001. Source data are provided as a Source Data file.

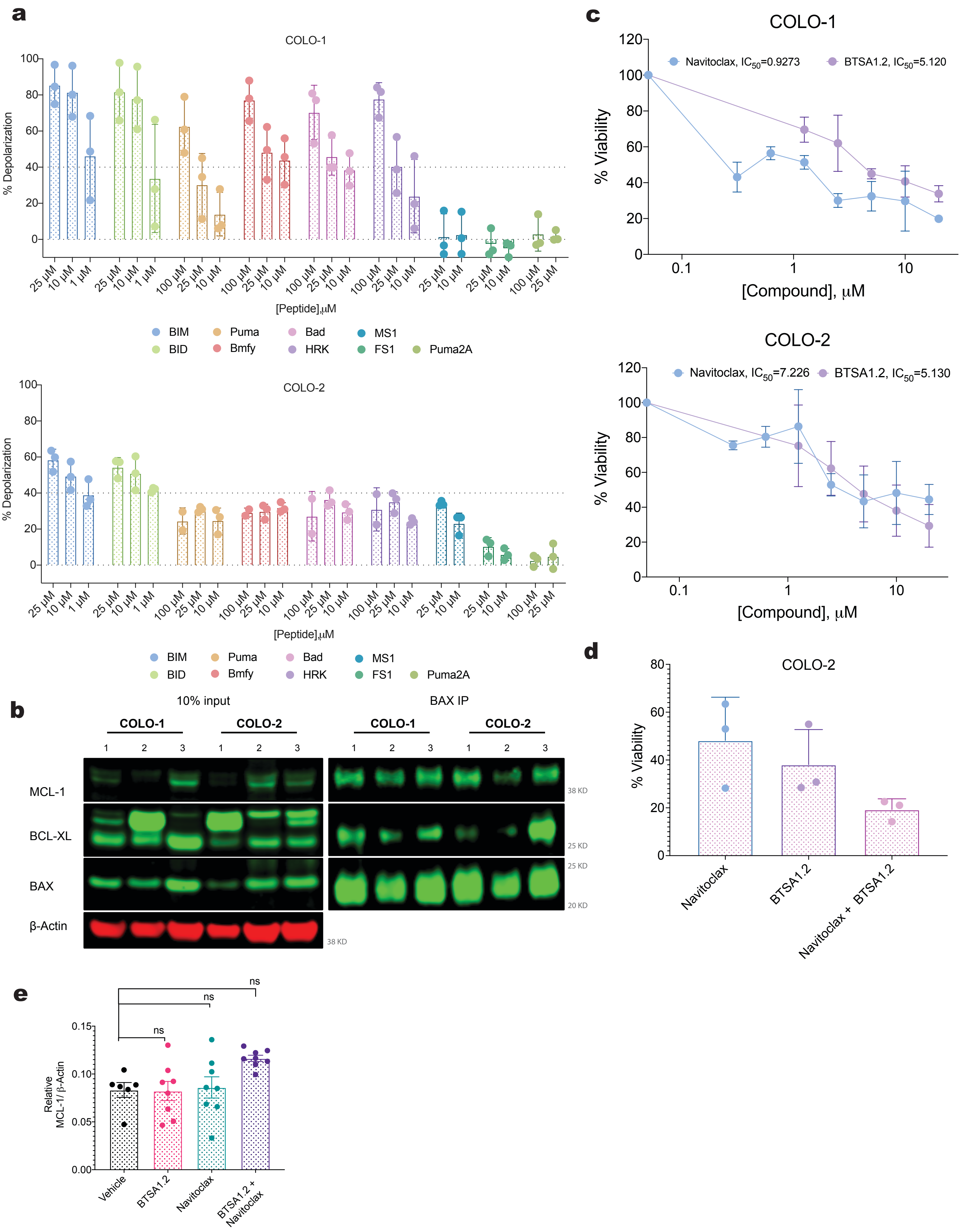
a
COLO-1
% Depolarization
[Peptide],µM
25 µM
10 µM
1 µM
100 µM
BIM
BID
Puma
Bmfy
Bad
HRK
MS1
FS1
Puma2A
COLO-2
b
10% input
BAX IP
COLO-1
COLO-2
1
2
3
MCL-1
BCL-XL
BAX
β-Actin
38 KD
25 KD
20 KD
c
COLO-1
Navitoclax, IC50=0.9273
BTSA1.2, IC50=5.120
% Viability
[Compound], µM
COLO-2
Navitoclax, IC50=7.226
BTSA1.2, IC50=5.130
d
COLO-2
% Viability
Navitoclax
BTSA1.2
Navitoclax + BTSA1.2
e
Relative MCL-1/ β-Actin
ns
Vehicle
BTSA1.2
Navitoclax
BTSA1.2 + Navitoclax

Supplementary Figure 13. Characterization of colorectal PDX.

a, BH3-profile of two colorectal PDXs. Bar graph represent % of mitochondria depolarization of isolated tumor cells detected by JC-1 upon treatment BH3-derived peptides. Data represent mean ± SD of three replicates from n=3 mice. **b**, Western blot analysis of BAX Co-IP in colorectal PDX tumors. Blot is representative of n=3 independent experiments. **c**, Cell viability of COLO-1 and COLO-2 PDX isolated cells after 24 hrs treatment with Navitoclax or BTSA1.2. **d**, Cell viability of COLO-2 PDX isolated cells after 24 hrs treatment with 10 μM Navitoclax, 10 μM BTSA1.2 or combination. Data represent mean ± SD of three replicates from n=3 independent experiments. **e**, Relative MCL-1 levels of COLO-1 tumors. Relative protein levels were normalized to β-Actin loading control. Data represent mean ± SD from n=6 mice (vehicle) and from n=8 mice (BTSA1.2, Navitoclax, Combination). Source data are provided as a Source Data file.

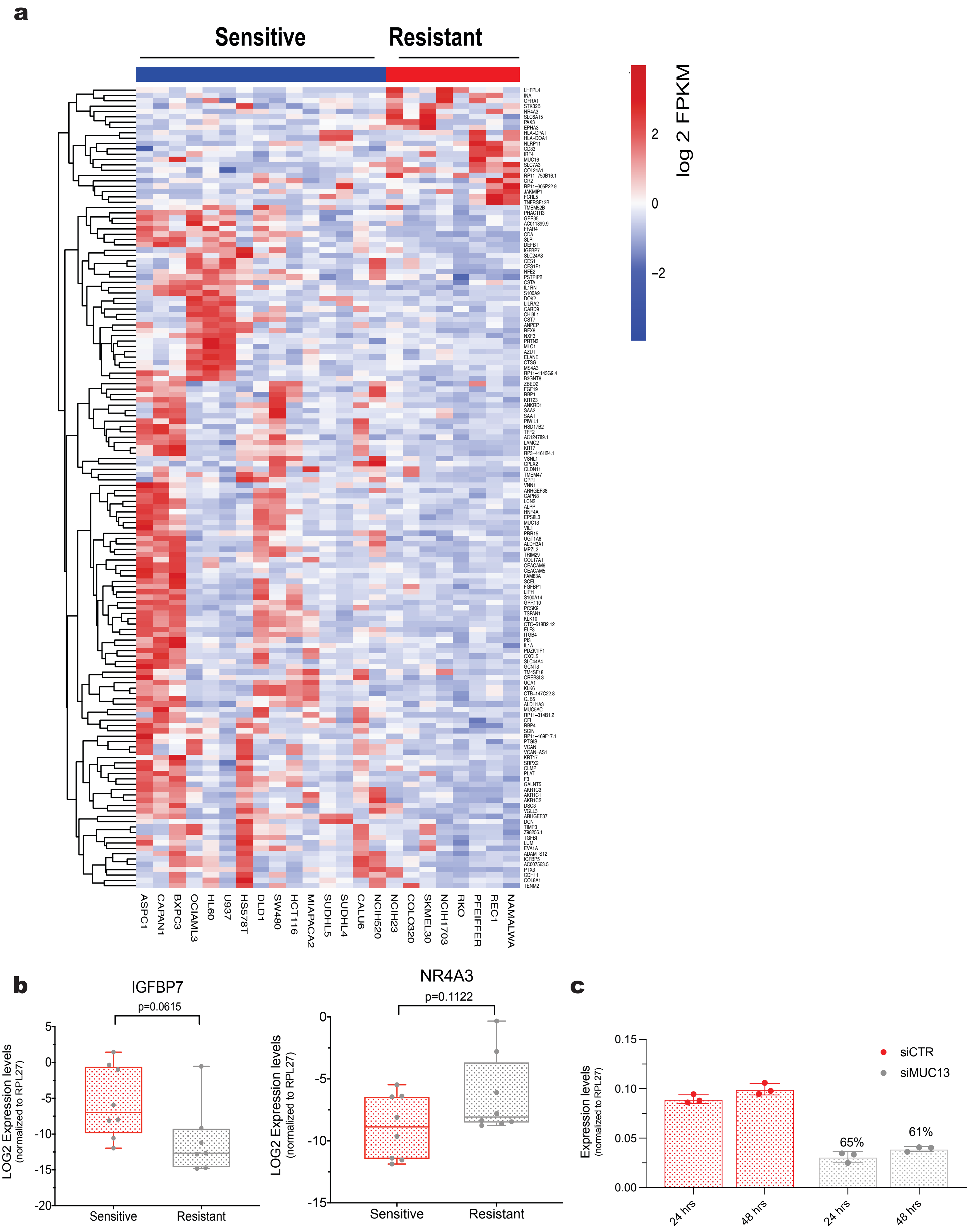
a
Sensitive
Resistant
log 2 FPKM
2
0
−2
ASPC1
CAPAN1
BXPC3
OCIAML3
HL60
U937
HS578T
DLD1
SW480
HCT116
MIAPACA2
SUDHL5
SUDHL4
CALU6
NCIH520
NCIH23
COLO320
SKMEL30
NCIH1703
RKO
PFEIFFER
REC1
NAMALWA
b
IGFBP7
p=0.0615
LOG2 Expression levels
(normalized to RPL27)
5
0
-5
-10
-15
-20
Sensitive
Resistant
NR4A3
p=0.1122
LOG2 Expression levels
(normalized to RPL27)
0
-5
-10
-15
Sensitive
Resistant
c
Expression levels
(normalized to RPL27)
0.15
0.10
0.05
0.00
siCTR
siMUC13
65%
61%
24 hrs
48 hrs
24 hrs
48 hrs

Supplementary Figure 14. Predictive biomarkers for Navitoclax and BTSA1.2 combination sensitivity.

a, Heatmap showing top 150 (selected by adjusted p-value) differentially expressed genes comparing resistant and sensitive cell lines based on IC50 change from Navitoclax alone to BTSA1.2 and Navitoclax combined. **b**, Box plot of validation of top hits associated with sensitivity and resistance to the combination by RT-qPCR in cell lines categorized as sensitive or resistant to the combination (corresponding to Fig. 2a). Relative gene expression was normalized using RPL27. Analysis was done in 7 sensitive cell lines and 6 resistant cell lines. The lines within the boxes show the median expression values, the box denotes the IQR, while the whiskers indicate maxima and minima values. Data are mean ± SD from n=3 independent experiments. Statistics was calculated using unpaired t-test. **c**, Gene expression of MUC13 in SW480 cell transfected with siRNA CTR or siRNA MUC13. Data are mean ± SD from n=3 independent experiments. Source data are provided as a Source Data file.

Supplementary Table 1. Structure-activity relationships of BAX activators based on *in vitro* binding and cellular activity

Compounds	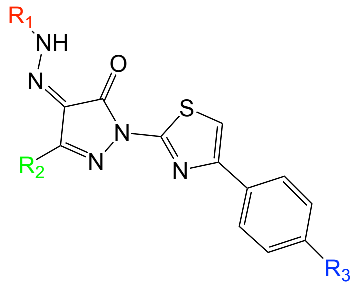 Definition of R1, R2, R3 groups	BIM SAHB - BAX Competition - FPA IC50 (nM)	Viability Inhibition - SU-DHL5 - IC50 (μM)
BTSA1.2	R1 = 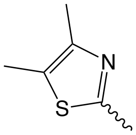 R2 = 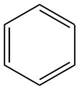 R3= H	149 ± 36	0.23 ± 0.02
BTSA1	R1 = 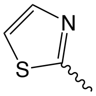 R2 = 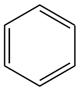 R3= H	247 ± 30	0.44 ± 0.04
BAM7	R1 = 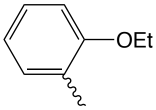 R2 = Me R3= H	3483 ± 979	>25
Compound 4	R1 = 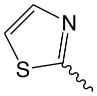 R2 = Me R3= H	2344 ± 838	>25
Compound 5	R1 = 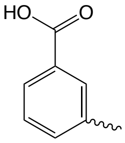 R2 = 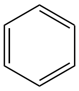 R3= H	497 ± 74	2.8 ± 1
Compound 6	R1 = 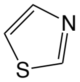 R2 = 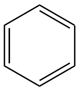 R3= 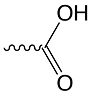	504 ± 92	16.1 ± 4.2

Measurements of IC50 based on competitive fluorescence polarization assay of the FITC-BIM SAHB - BAX complex and viability assays in SU-DHL-5 cell line.

Supplementary Table 2. Stability of compounds in the presence of mouse liver microsomes.

	% Remaining of Initial Compound				
**Compound**	0 min	10 min	20 min	30 min	60 min
BTSA1.2	100	107	109	100	97
BTSA1	100	98	93	96	68

Supplementary Table 3. Genomic alterations in panel of human cancer cell lines

Tissue	Cell line	TP53 status	RAS status	BRAF status	PIK3CA status
Colorectal	RKO	WT	WT	Mutant	Mutant
	HCT-116	WT	Mutant	Mutant	Mutant
	HT-29	Mutant	WT	Mutant	Mutant
	SW480	Mutant	Mutant	WT	WT
	COLO320	Mutant	WT	WT	WT
	DLD1	Mutant	Not avaliable	Not avaliable	Not avaliable
Breast	Hs-578-T	Mutant	WT	WT	WT
	MCF-7	WT	WT	WT	Mutant
	MDA-MB-231	Mutant	Mutant	Mutant	WT
	LM2	Mutant	Mutant	Mutant	Not avaliable
	SK-BR-3	Mutant	WT	WT	WT
Pancreatic	BxPC-3	Mutant	WT	Mutant	WT
	MIA-PaCa-2	Mutant	Mutant	WT	WT
	Capan-1	Mutant	Mutant	WT	WT
	Capan-2	Negative	Mutant	WT	WT
	AsPC-1	Negative	Mutant	WT	WT
NSCLC	NCI-H460	WT	Mutant	WT	Mutant
	HOP-92	Mutant	Mutant	WT	WT
	NCI-H23	Mutant	Mutant	WT	WT
	NCI-H1703	Negative	WT	WT	WT
	NCI-H520	Mutant	WT	WT	WT
	Calu-6	Mutant	Mutant	WT	WT
Melanoma	SK-MEL-28	Mutant	WT	Mutant	Mutant
	SK-MEL-30	WT	WT	Mutant	WT
	A375	WT	WT	Mutant	WT
Lymphoma	Farage	Mutant	WT	WT	WT
	SU-DHL-4	Mutant	WT	WT	WT
	SU-DHL-5	WT	WT	WT	WT
	SU-DHL-6	Mutant	WT	WT	WT
	SU-DHL-16	Negative	WT	WT	WT
	Namalwa	Mutant	WT	WT	WT
	Pfeiffer	Negative	WT	WT	WT
	REC-1	Mutant	WT	WT	WT
Leukemia	SKM1	Mutant	Mutant	WT	WT
	U937	Mutant	WT	WT	WT
	HEL	Mutant	WT	WT	WT
	HL60	Negative	Mutant (NRAS)	WT	WT
	OCIAML3	WT	Mutant (NRAS)	WT	WT
	ML2	WT	Mutant	WT	WT

Supplementary Table 4. Top 150 differentially expressed genes in cell lines resistant or sensitive to the combination

gene	log2FC	padj*
ENSG00000038427.11:VCAN	8.07	0
ENSG00000148798.5:INA	-7.75	2E-15
ENSG00000198758.6:EPS8L3	7.98	7E-15
ENSG00000257764.2:RP11-1143G9.4	7.68	4.064E-12
ENSG00000249835.2:VCAN-AS1	7.79	1.3369E-11
ENSG00000129451.7:KLK10	6.86	3.8403E-11
ENSG00000162366.3:PDZK1IP1	6.98	1.0235E-10
ENSG00000173702.3:MUC13	5.64	1.781E-10
ENSG00000123405.9:NFE2	6.22	1.781E-10
ENSG00000114115.5:RBP1	6.25	1.781E-10
ENSG00000183111.7:ARHGEF37	4.97	1.781E-10
ENSG00000147443.8:DOK2	6.54	3.416E-10
ENSG00000189280.3:GJB5	6.32	5.2806E-10
ENSG00000144810.11:COL8A1	7.23	7.0533E-10
ENSG00000136689.14:IL1RN	6.92	9.324E-10
ENSG00000151388.6:ADAMTS12	6.67	9.3405E-10
ENSG00000115461.4:IGFBP5	6.63	9.3405E-10
ENSG00000163735.6:CXCL5	6.38	1.8778E-09
ENSG00000117525.9:F3	5.96	2.2505E-09
ENSG00000128422.11:KRT17	6.17	4.5033E-09
ENSG00000162344.3:FGF19	6.97	5.7673E-09
ENSG00000101076.12:HNF4A	5.43	7.9375E-09
ENSG00000100448.3:CTSG	7.35	8.2191E-09
ENSG00000108244.12:KRT23	6.27	8.3882E-09
ENSG00000214049.6:UCA1	5.77	8.8483E-09
ENSG00000135480.10:KRT7	6.2	9.1776E-09
ENSG00000198848.8:CES1	5.76	9.1776E-09
ENSG00000172232.5:AZU1	6.6	1.0157E-08
ENSG00000204385.6:SLC44A4	5.97	1.5157E-08
ENSG00000120708.12:TGFBI	5.91	1.7683E-08
ENSG00000196415.5:PRTN3	6.4	1.9607E-08
ENSG00000163032.7:VSNL1	5.48	2.29E-08
ENSG00000134762.12:DSC3	5.87	2.5095E-08
ENSG00000178623.7:GPR35	4.67	4.2854E-08
ENSG00000268739.1:CTC-518B2.12	7.08	4.448E-08
ENSG00000167165.14:UGT1A6	5.57	4.448E-08

ENSG00000147027.3:TMEM47	6	4.5726E-08
ENSG00000167755.9:KLK6	5.49	5.0234E-08
ENSG00000148346.7:LCN2	5.61	5.0933E-08
ENSG00000086696.6:HSD17B2	6.29	6.7697E-08
ENSG00000108602.13:ALDH3A1	6.18	7.3833E-08
ENSG00000153292.11:GPR110	6.32	7.3833E-08
ENSG00000124212.5:PTGIS	5.72	1.6141E-07
ENSG00000136542.4:GALNT5	5.81	1.8016E-07
ENSG00000166825.9:ANPEP	5.99	1.9406E-07
ENSG00000140297.8:GCNT3	5.19	2.3699E-07
ENSG00000138207.8:RBP4	4.91	2.4075E-07
ENSG00000257671.1:RP3-416H24.1	5.6	2.6048E-07
ENSG00000215182.6:MUC5AC	5.3	2.7323E-07
ENSG00000149516.9:MS4A3	6.94	2.8385E-07
ENSG00000086548.8:CEACAM6	5.55	2.9162E-07
ENSG00000100427.11:MLC1	5.64	3.1312E-07
ENSG00000117472.5:TSPAN1	5.64	3.1312E-07
ENSG00000124107.5:SLPI	5.64	3.6677E-07
ENSG00000206538.3:VGLL3	5.52	5.1676E-07
ENSG00000272259.1:RP11-305P22.9	-7.07	6.0774E-07
ENSG00000160181.4:TFF2	6.32	7.5091E-07
ENSG00000236886.2:AC007563.5	6.43	7.5091E-07
ENSG00000156959.7:LHFPL4	-6.21	7.8082E-07
ENSG00000133048.8:CHI3L1	5.05	8.4329E-07
ENSG00000165349.7:SLC7A3	-6.42	8.9781E-07
ENSG00000197561.2:ELANE	6.34	1.2126E-06
ENSG00000105388.10:CEACAM5	4.84	1.3457E-06
ENSG00000177191.2:B3GNT8	4.58	1.3587E-06
ENSG00000186188.6:FFAR4	6.13	1.3971E-06
ENSG00000149573.4:MPZL2	5.34	1.4141E-06
ENSG00000228695.5:CES1P1	5.89	1.5455E-06
ENSG00000184254.12:ALDH1A3	5.07	1.5892E-06
ENSG00000119508.13:NR4A3	-4.31	1.616E-06
ENSG00000139329.4:LUM	5.18	1.7938E-06
ENSG00000236699.4:ARHGEF38	5.03	1.9035E-06
ENSG00000145934.11:TENM2	4.46	1.9228E-06
ENSG00000104368.13:PLAT	5.37	1.9355E-06
ENSG00000147206.12:NXF3	5.53	1.9355E-06
ENSG00000196139.7:AKR1C3	5.04	2.4444E-06
ENSG00000179873.10:NLRP11	-5.29	2.479E-06
ENSG00000163435.11:ELF3	5.21	2.8223E-06

ENSG00000177494.5:ZBED2	4.84	2.8893E-06
ENSG00000163283.6:ALPP	5.03	2.8893E-06
ENSG00000187134.8:AKR1C1	4.83	3.0885E-06
ENSG00000100234.11:TIMP3	5.62	3.0981E-06
ENSG00000145920.10:CPLX2	4.92	3.2478E-06
ENSG00000231389.3:HLA-DPA1	-5.19	3.2478E-06
ENSG00000143297.14:FCRL5	-6.02	3.7815E-06
ENSG00000163453.7:IGFBP7	4.2	4.0792E-06
ENSG00000269495.1:CTB-147C22.8	5.15	4.3001E-06
ENSG00000102359.5:SRPX2	5.26	4.4848E-06
ENSG00000171502.10:COL24A1	-3.59	4.6451E-06
ENSG00000240505.4:TNFRSF13B	-5.04	4.6939E-06
ENSG00000077984.4:CST7	5.67	5.0956E-06
ENSG00000151892.10:GFRA1	-4.57	5.3997E-06
ENSG00000203697.7:CAPN8	5.5	5.3997E-06
ENSG00000183531.1:Z98256.1	5.62	5.3997E-06
ENSG00000196460.8:RFX8	5.65	5.6735E-06
ENSG00000013297.6:CLDN11	4.43	5.6888E-06
ENSG00000163220.10:S100A9	5.02	5.8496E-06
ENSG00000183671.8:GPR1	5.16	7.2305E-06
ENSG00000152953.8:STK32B	-4.65	7.2305E-06
ENSG00000124102.4:PI3	5.09	7.2305E-06
ENSG00000169174.9:PCSK9	5.23	7.2305E-06
ENSG00000127831.6:VIL1	3.84	7.2305E-06
ENSG00000112299.7:VNN1	5.19	7.2382E-06
ENSG00000058085.10:LAMC2	5.06	7.2382E-06
ENSG00000152229.14:PSTPIP2	4.04	7.6674E-06
ENSG00000163762.2:TM4SF18	5.27	7.7029E-06
ENSG00000263711.1:RP11-169F17.1	6.38	7.9139E-06
ENSG00000112149.5:CD83	-3.62	8.1629E-06
ENSG00000273301.1:RP11-314B1.2	6.02	9.0249E-06
ENSG00000164825.3:DEFB1	5.85	9.0249E-06
ENSG00000137699.12:TRIM29	4.7	9.1608E-06
ENSG00000185052.7:SLC24A3	6.12	1.0066E-05
ENSG00000165685.4:TMEM52B	5.16	1.0066E-05
ENSG00000136155.12:SCEL	4.49	1.0066E-05
ENSG00000137440.3:FGFBP1	4.15	1.0066E-05
ENSG00000134339.4:SAA2	5.09	1.0066E-05
ENSG00000132470.9:ITGB4	4.6	1.0265E-05
ENSG00000011465.12:DCN	5	1.0556E-05
ENSG00000163661.3:PTX3	4.79	1.0624E-05

ENSG00000148677.6:ANKRD1	4.51	1.0632E-05
ENSG00000137265.10:IRF4	-5.18	1.107E-05
ENSG00000181143.11:MUC16	-4.1	1.107E-05
ENSG00000233038.1:AC011899.9	5.57	1.1159E-05
ENSG00000158825.5:CDA	4.84	1.1623E-05
ENSG00000163898.5:LIPH	5.03	1.1623E-05
ENSG00000152969.12:JAKMIP1	-5.89	1.1749E-05
ENSG00000196735.7:HLA-DQA1	-4.81	1.1749E-05
ENSG00000072041.12:SLC6A15	-5.24	1.2699E-05
ENSG00000189334.4:S100A14	5.21	1.3559E-05
ENSG00000173432.6:SAA1	4.74	1.5325E-05
ENSG00000205403.8:CFI	4.72	1.5457E-05
ENSG00000115363.9:EVA1A	4.7	1.5542E-05
ENSG00000166250.7:CLMP	4.22	1.6195E-05
ENSG00000087495.12:PHACTR3	5.65	1.6195E-05
ENSG00000121552.3:CSTA	5.09	1.6195E-05
ENSG00000140937.9:CDH11	4.97	1.6889E-05
ENSG00000176532.3:PRR15	4.95	1.7318E-05
ENSG00000151632.12:AKR1C2	4.05	1.9542E-05
ENSG00000115008.5:IL1A	3.22	2.0043E-05
ENSG00000065618.12:COL17A1	4.17	2.0043E-05
ENSG00000117322.12:CR2	-4.83	2.0172E-05
ENSG00000125207.3:PIWIL1	5.88	2.0545E-05
ENSG00000135903.14:PAX3	-6.01	2.3041E-05
ENSG00000260833.2:AC124789.1	5.5	2.3208E-05
ENSG00000006747.10:SCIN	3.96	2.4457E-05
ENSG00000044524.6:EPHA3	-4.51	2.4663E-05
ENSG00000262902.1:RP11-750B16.1	-4.4	2.5078E-05
ENSG00000187796.9:CARD9	4	2.5121E-05
ENSG00000060566.9:CREB3L3	4.89	2.5121E-05
ENSG00000147689.12:FAM83A	4.48	2.5714E-05

* Statistics were obtained using a two sided Wald test, p-values attained were corrected for multiple testing using the Benjamini and Hochberg method (FDR, shown as padj in the table).

Supplementary Table 5. Characteristics of colorectal patient samples

PDXs	Age	Tissue Source	TNM staging	MSS/MSI	BRAF Mutation	KRAS Mut	PIK3CA Mut
COLO-1	74	CRC Primary - Right colon	T4aN0Mx	MSI-H	BRAF_v600E		
COLO-2	35	CRC - Liver metastasis	T2N1Mx	MSI-H		KRAS_G13D	PIK3CA_E112K

Supplementary Table 6. Primers used in the study for gene expression quantification

Gene	Forward primer	Reverse primer
BCL2L1	TGCAGGTATTGGTGAGTCGG	ACAAAAGTATCCCAGCCGCC
EPS8L3	CCTACCAACCCACATTCTCAG	TCCCTAACCTATGACTTCCCC
IGFBP7	TGCCATGCATCCAATTCCCA	TATAGCTCGGCACCTTCACCTTT
IRF4	GTGAAAATGGTTGCCAGGTGA	AGGCTTCGGCAGACCTTATG
MUC13	GTAACCAGACTGCGGATGACT	AGACTGGAAGCAACGCAGAAA
NR4A3	GCTGGGCAGAAAAGATTCCG	CAGCAGTGTTTGACCTGATGG
RPL27	CATGGGCAAGAAGAAGATCG	TCCAAGGGGATATCCACAGA
SLC7A3	TAAGACTCTGCAGGGGTCCA	CCGAGAGCCAACAATCCAGT

Supplementary Methods:

Chemical Synthesis

Materials and Methods for the Chemical Syntheses. All chemical reagents and solvents were obtained from commercial sources (Aldrich, Acros, Fisher) and used without further purification unless otherwise noted. Anhydrous solvents (tetrahydrofurane, toluene, dichloromethane, diethyl ether) were obtained using a Pure Solv™ AL-258 solvent purification system. Ethanol was dried over activated 4 Å molecular sieves. Microwave reactions were performed on an Anton Paar Monowave 300. Chromatography was performed on a Teledyne ISCO CombiFlash R_f 200i using disposable silica cartridges (4, 12, and 24 g). Analytical thin layer chromatography (TLC) was performed on aluminum-backed Silicycle silica gel plates (250 μm film thickness, indicator F254). Compounds were visualized using a dual wave length (254 and 365 nm) UV lamp, and/or staining with CAM (cerium ammonium molybdate) or $KMnO_4$ stains. NMR spectra were recorded on Bruker DRX 300 and DRX 600 spectrometers. ^{1}H and ^{13}C chemical shifts (δ) are reported relative to tetramethyl silane (TMS, 0.00/0.00 ppm) as internal standard or to residual solvent (CD_3OD: 3.31/49.00 ppm; $CDCl_3$: 7.26/77.16 ppm; dmso-d_6: 2.50/39.52 ppm). Mass spectra were recorded on a Shimadzu LCMS 2010EV (direct injection unless otherwise noted). High resolution electrospray ionization mass spectra (ESI-MS) were obtained at the Albert Einstein College of Medicine's Laboratory for Macromolecular Analysis and Proteomics. Or obtained externally from Intertek USA, Inc. (Whitehouse, NJ;

http://www.intertek.com/pharmaceutical/analysis/whitehouse-nj/). Unless otherwise noted the purity of the compounds synthesized was ≥ 95% as judged by the ^{1}H-NMR trace.

Synthesis of BTSA 1.2 (S5)

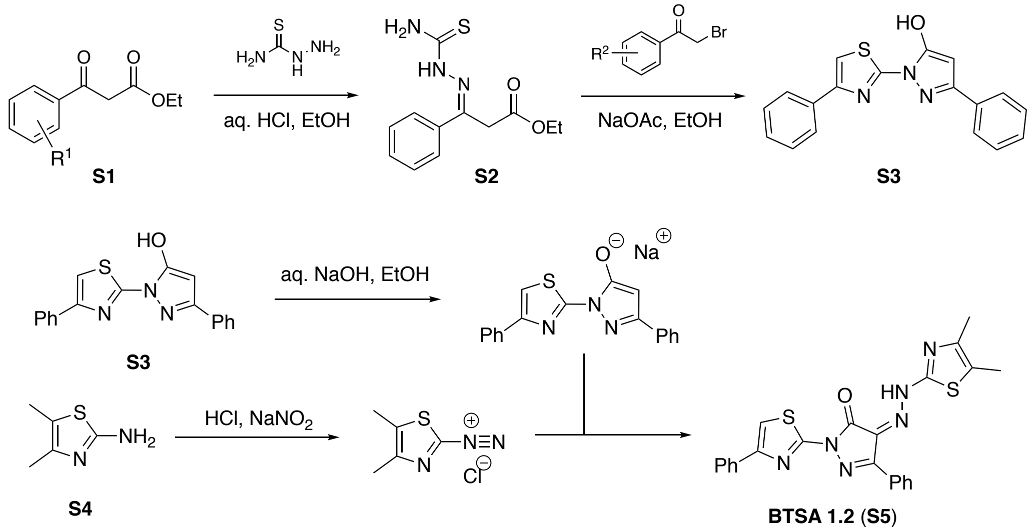

Scheme S1: Synthesis of **BTSA 1.2** (**S5**; overview)

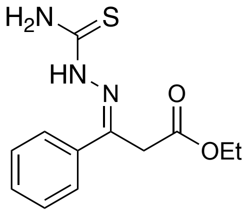

Synthesis of ethyl-3-(2-carbamothioylhydrazono)-3-phenylpropanoate (S2):

Hydrazinecarbothioamide (2.00 g, 22.0 mmol, 1.10 equiv) was dissolved in 5% aq. HCl (64.2 mL, 90 mmol, 4.50 equiv). and ethanol (30.0 mL). Ethyl 3-oxo-3-phenylpropanoate (3.83 g, 20.0 mmol, 1.00 equiv) was added with vigorous stirring. The resulting mixture

was kept stirring vigorously at RT overnight (>12 h). White precipitate that had formed was filtered and washed with little water. The resulting white, fluffy solid (4.24 g) was washed with EtOH (15.0 mL), filtered and dried in high vacuum to obtain ethyl (Z)-3-(2-carbamothioylhydrazono)-3-phenylpropanoate (**S2**; 3.54 g, 13.3 mmol, 67 %, purity ≥90 % by ^{1}H-NMR). The crude product was used directly in the next step without further purification.

^{1}H-NMR (600 MHz, dmso-d_6): δ 10.62 (s, 1H), 8.38 (s, 1H), 8.03 (s, 1H), 7.88–7.85 (m, 2H), 7.39–7.37 (m, 3H), 4.10–4.06 (m, 4H), 1.16 (t, J = 7.1 Hz, 3H). **^{13}C-NMR** (151 MHz, dmso-d_6): δ 179.2, 168.3, 142.3, 136.8, 129.2, 128.3, 126.5, 60.8, 33.2, 14.0.

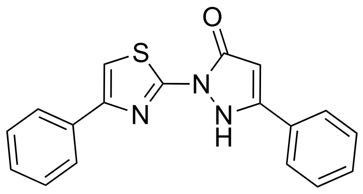

Synthesis of 5-phenyl-2-(4-phenylthiazol-2-yl)-1,2-dihydro-3H-pyrazol-3-one (S3):

In a 60 mL centrifuge tube with stir bar and cap, ethyl-3-(2-carbamothioylhydrazono)-3-phenylpropanoate (**S2**; 1.06 g, 4.00 mmol, 1.00 equiv) and 2-bromo-1-phenylethan-1-one (1.04 g, 5.20 mmol, 1.30 equiv) were suspended in Ethanol (21 mL). The mixture was stirred at RT. Almost immediately, all material dissolved, and ca. 1 min later, a thick, white precipitate formed. After 60 min, sodium acetate (492 mg, 6.00 mmol, 1.50 equiv) was added, and the mixture was kept stirring overnight (>12 h) at RT. 20.0 mL of water were added, the mixture filtered under reduced pressure. Residual material in the reaction vessel was rinsed out with little more water. The residue obtained was washed with more water (total volume of the collected aqueous phases: 60.0 mL). The crude product was

obtained as an off white solid that was dried in high vacuum. After purification on the Isco CombiFlash (0.0→60.0 % CH_2Cl_2 in hexanes), **5**-phenyl-2-(4-phenylthiazol-2-yl)-1,2-dihydro-3H-pyrazol-3-one (**S3**) was obtained as colorless solid (850 mg, 2.66 mmol, 71 %).

TLC: R_f 0.75 (50 % CH_2Cl_2 in hexanes). **^{1}H-NMR** (600 MHz, dmso-d_6 + 2dr py- d_5)[1]: δ 8.03 (d, J = 7.3 Hz, 2H), 7.89 (d, J = 7.3 Hz, 2H), 7.86 (s, 1H), 7.51–7.45 (m, 5H), 7.37–7.34 (m, 1H), 6.08 (s, 1H). **^{13}C-NMR** (151 MHz, dmso-d_6 + 2dr py- d_6): δ 158.5, 155.9, 153.0, 150.1, 133.9, 130.4, 129.7, 128.8, 128.7, 128.0, 126.1, 126.0, 109.8, 88.2. **ESI-MS** *m/z* (rel int): (320.1 ([M+H]$^+$, 100). **HRMS** calculated for $C_{18}H_{14}N_3OS$ (M+H) 320.0852, found: 320.0853.

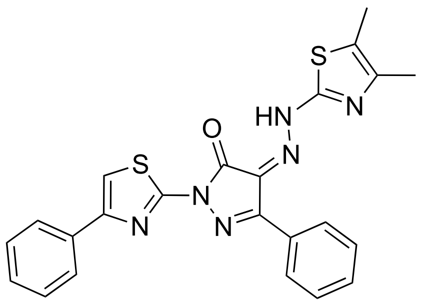

Synthesis of 4-(2-(4,5-dimethylthiazol-2-yl)hydrazono)-5-phenyl-2-(4-phenyl-thiazol-2-yl)-2,4-dihydro-3H-pyrazol-3-one (S5; BTSA 1.2):

To a suspension of 4,5-dimethylthiazol-2-amine (120 mg, 0.939 mmol) in hydrochloric acid (half concentrated, 0.75 mL, 12.4 mmol, 13.2 equiv) in an open vessel in a wet ice/NaCl cooling bath (temperature was kept at ≤ –5 ˚C) was added drop-wise a solution of sodium nitrite (64.8 mg, 0.939 mmol, 1.00 equiv) in water (0.47 mL) via pipette. The

[1] If the NMR is taken in pure dmso-d_6, a mixture of tautomers is observed. Addition of pyridine shifts the equilibrium entirely to one side, allowing for the detection of one distinct set of signals.

diazonium salt was formed as a deep yellow solution. The starting material had dissolved completely after ca 10 min and the solution was stirred at ≤ –5 ˚C for another 15 min. In parallel, 5-phenyl-2-(4-phenylthiazol-2-yl)-2,4-dihydro-3H-pyrazol-3-one (**S3**; 300 mg, 0.939 mmol, 1.00 equiv) was dissolved in aqueous sodium hydroxide (2.50 M; 0.94 mL, 2.35 mmol, 2.50 equiv) and ethanol (0.94 mL). A clear solution formed after a few minutes and was stirred for another 10 min at RT [*Note*: in case of some of the substituted analogs, the solubility is considerably lower, resulting either in incomplete dissolution or precipitate forming from a solution initially formed. In these cases, water/ethanol (1:1) is added in small increments until a solution is formed. The solution of the anionic species formed above was then added to the diazonium salt in a drop-wise fashion. [*Note*: It is crucial to maintain proper cooling and mixing throughout the addition!]. Deep red precipitate formed almost instantaneously. After complete addition, the mixture was warmed to RT and stirred for another 20 min. TLC analysis of a reaction aliquot (either directly diluted in little MeOH or subjected to a H_2O/EtOAc micro-workup) indicated consumption of the thiazol amine, formation of a new product, some more polar colored byproducts, as well remaining **S3**. The mixture was diluted with water (1.0 mL), filtered into a Buchner funnel with filter paper, washed with little water (ca. 3.0 mL), then dried in the air stream of the filtration apparatus. The pre-dried material was transferred into a flask and dried additionally in high vacuum. After purification on the Isco CombiFlash (0.1→5.0 % MeOH in CH_2Cl_2), 4-(2-(4,5-dimethylthiazol-2-yl)hydrazono)-5-phenyl-2-(4-phenyl-thiazol-2-yl)-2,4-dihydro-3H-pyrazol-3-one (**BTSA 1.2** (**S5**); 261 mg, 0.569 mmol 61 %) was obtained as bright red solid.

[*Note:* This synthetic protocol is highly sensitive to mixing/cooling issues, which are complicated by the fact that the product and re-protonated intermediate precipitate during the reaction. We noticed that in some cases, considerable amounts of impurities, stemming from the decomposition of the diazonium reagent, can be formed. Additional chromatography and/or re-crystallization (most commonly from dioxane) of the product is required in these cases. As a result, yields can be low, despite often acceptable conversions, as judged by TLC.

TLC: R_f 0.90 (5 % MeOH in CH_2Cl_2). **^{1}H-NMR** (600 MHz, dmso-d_6): δ 8.14 (d, J = 7.1 Hz, 2H), 7.99 (dd, J = 8.1, 1.0 Hz, 2H), 7.80 (s, 1H), 7.54–7.45 (m, 5H), 7.35 (t, J = 7.3 Hz, 1H), 2.25 (s, 3H), 2.18 (s, 3H). **^{13}C-NMR** (151 MHz, dmso-d_6): δ 177.7, 154.7, 152.5, 149.7, 149.2, 134.9, 134.2, 130.9, 129.7, 129.6, 128.7, 128.4, 128.0, 127.9, 125.9, 119.1, 108.8, 11.7, 11.4. **ESI-MS** *m/z* (rel int): (459.1 ([M+H]$^+$, 100). **HRMS** calculated for $C_{22}H_{13}N_6O_3S_2$ (M+H) 459.1056, found: 459.1051.

^{1}H- and ^{13}C-NMR traces of BTSA 1.2 (S5)

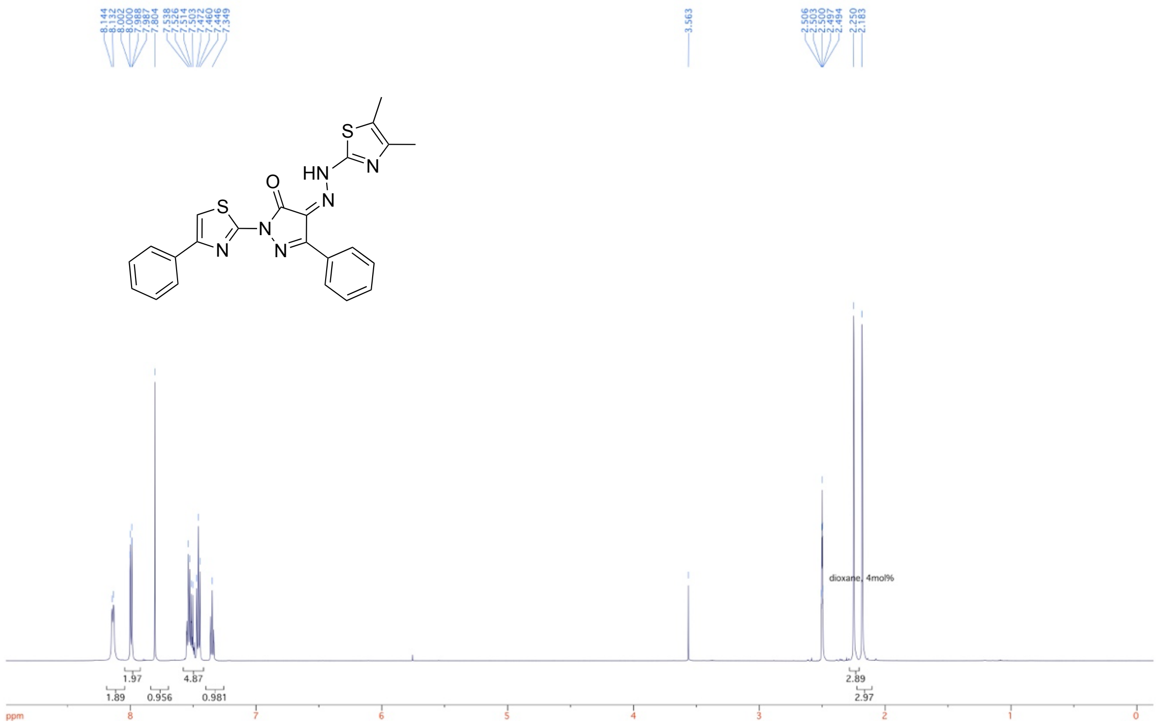
8.144
8.132
8.002
8.000
7.988
7.987
7.804
7.538
7.526
7.514
7.503
7.472
7.460
7.446
7.349
3.563
2.506
2.503
2.500
2.497
2.494
2.250
2.183
HN
N
S
N
O
N
N
S
N
dioxane, 4mol%
1.97
1.89
0.956
4.87
0.981
2.89
2.97
ppm
8
7
6
5
4
3
2
1
0

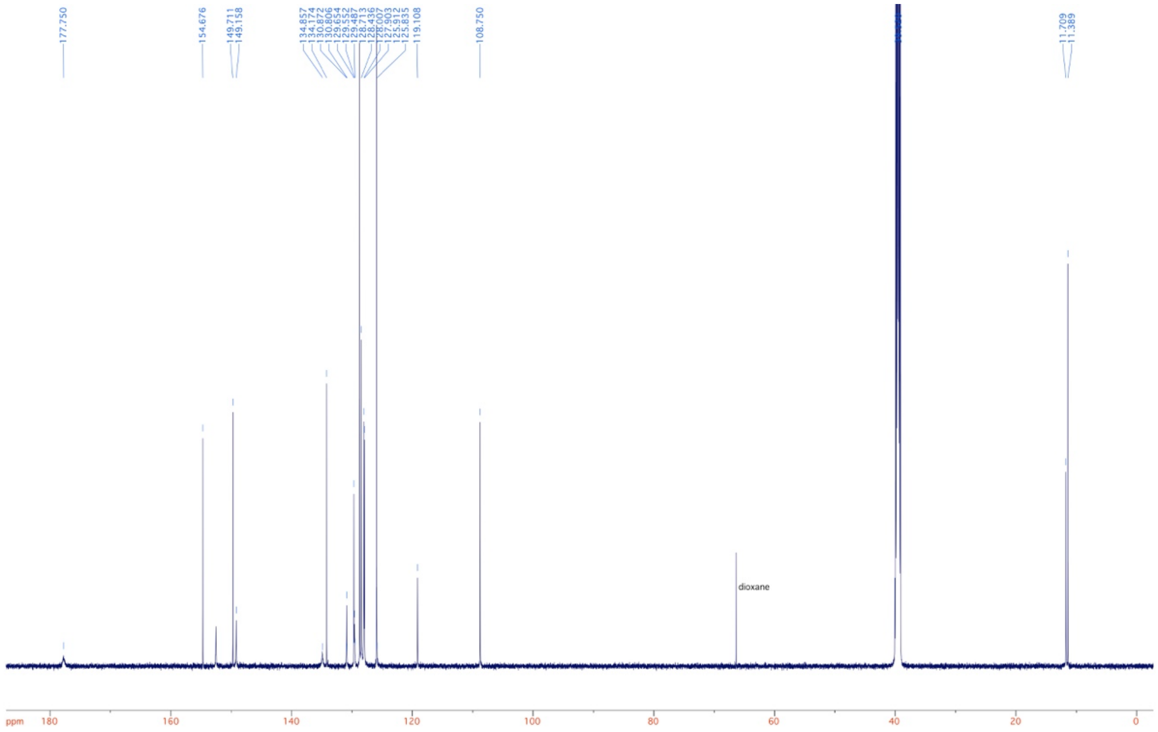
177.750
154.676
149.711
149.158
134.857
134.174
130.872
130.806
129.654
129.552
129.487
128.713
128.436
128.007
127.903
125.912
125.835
119.108
108.750
11.709
11.389
dioxane
ppm
180
160
140
120
100
80
60
40
20
0
