# Supplementary material for: Co-targeting of BAX and BCL-XL proteins broadly overcomes resistance to apoptosis in cancer
Source: Nat Commun. 2022 Mar 7;13:1199. doi: 10.1038/s41467-022-28741-7 (PMC8901805; doi:10.1038/s41467-022-28741-7)
Supplement: Supplementary file 2 — Reporting Summary [file 41467_2022_28741_MOESM2_ESM.pdf]

nature research

Corresponding author(s): Evripidis Gavathiotis

Last updated by author(s): 1-27-2022

Reporting Summary

Nature Research wishes to improve the reproducibility of the work that we publish. This form provides structure for consistency and transparency in reporting. For further information on Nature Research policies, see our Editorial Policies and the Editorial Policy Checklist.

Statistics

For all statistical analyses, confirm that the following items are present in the figure legend, table legend, main text, or Methods section.

n/a	Confirmed	
☐	☒	The exact sample size (n) for each experimental group/condition, given as a discrete number and unit of measurement
☐	☒	A statement on whether measurements were taken from distinct samples or whether the same sample was measured repeatedly
☐	☒	The statistical test(s) used AND whether they are one- or two-sided<br>*Only common tests should be described solely by name; describe more complex techniques in the Methods section.*
☒	☐	A description of all covariates tested
☒	☐	A description of any assumptions or corrections, such as tests of normality and adjustment for multiple comparisons
☐	☒	A full description of the statistical parameters including central tendency (e.g. means) or other basic estimates (e.g. regression coefficient) AND variation (e.g. standard deviation) or associated estimates of uncertainty (e.g. confidence intervals)
☐	☒	For null hypothesis testing, the test statistic (e.g. F, t, r) with confidence intervals, effect sizes, degrees of freedom and P value noted<br>*Give P values as exact values whenever suitable.*
☒	☐	For Bayesian analysis, information on the choice of priors and Markov chain Monte Carlo settings
☒	☐	For hierarchical and complex designs, identification of the appropriate level for tests and full reporting of outcomes
☐	☒	Estimates of effect sizes (e.g. Cohen's d, Pearson's r), indicating how they were calculated

Our web collection on statistics for biologists contains articles on many of the points above.

Software and code

Policy information about availability of computer code

Data collection	Western blot data were collected with Odyssey Infrared Imaging System, Image Studio (Version 3.1, LI-COR Biosciences).<br>NMR data were collected and processed with Topspin software (Version 2.1, 2016, Bruker).
Data analysis	Data analysis and statistical comparisons were performed by Graphpad Prism 8.0-9.0 software. Western blot data were analyzed with Image Studio (Version 3.1, LICOR). Primers were designed using NCBI Primer-BLAST tool. The BLISS calculation was determined using the Combenefit program, version (2.021). Differential expression analysis was conducted using R package DESeq2 (version 1.32.0). Pharmacokinetics parameters were calculated using Phoenix WinNonlin 6.3.

For manuscripts utilizing custom algorithms or software that are central to the research but not yet described in published literature, software must be made available to editors and reviewers. We strongly encourage code deposition in a community repository (e.g. GitHub). See the Nature Research guidelines for submitting code & software for further information.

Data

Policy information about availability of data

All manuscripts must include a data availability statement. This statement should provide the following information, where applicable:

- Accession codes, unique identifiers, or web links for publicly available datasets
- A list of figures that have associated raw data
- A description of any restrictions on data availability

RNA-Seq raw counts data were retrieved from the Cancer Cell Line Encyclopedia (CCLE) database at BROAD Institute (https://portals.broadinstitute.org/ccle/data). TCGA expression data were obtained from publicly available datasets at cBioPortal (https://www.cbioportal.org). All the information generated and analyzed is included in the manuscript and all figures have associated raw data that is provided as an Excel worksheet organized by figures (Data Source file). Source data are provided with this paper.

Field-specific reporting

Please select the one below that is the best fit for your research. If you are not sure, read the appropriate sections before making your selection.

- [x] Life sciences
- [ ] Behavioural & social sciences
- [ ] Ecological, evolutionary & environmental sciences

For a reference copy of the document with all sections, see nature.com/documents/nr-reporting-summary-flat.pdf

Life sciences study design

All studies must disclose on these points even when the disclosure is negative.

Sample size	Sample sizes were designed based on related assays with our previous publications Reyna et al. Cancer Cell 2017, 32:490-505, doi: 10.1016/j.ccell.2017.09.001. and Dulguun et al., Nature Cancer, 2020, 1:315-328. doi: 10.1038/s43018-020-0039-1 . For the in vivo studies, a minimal number of animals for statistically significant results were used in compliance with the IACUC guidelines. Age and sex matched animals were used for all experiments. Sample sizes and statistical data are reported in figure legends.
Data exclusions	No data were excluded from the analyses.
Replication	Technical replicates and independent experiments were performed to verify reproducibility of the assays. All attempts at replication were successful. The experimental findings were reliably reproduced as described in the figure legends.
Randomization	For xenograft studies after confirmation of tumor engraftment, the mice were randomly assigned to four treatment groups. In other in vivo experiments, mice were randomly assigned into experimental groups. For studies involving cells in culture treatment groups were attributed randomly between wells and plates to account for well or tplate positioning effects.
Blinding	Binding was not possible for the mouse treatment experiments as drugs have distinct colors, red for BTSA1.2 and yellow for Navitoclax, and vehicle has transparent color. Investigators were blinded to group allocation and during data analysis for both in vivo and in vitro experiments and unblinding was done when the analysis was completed for plotting and image composition of the figures.

Reporting for specific materials, systems and methods

We require information from authors about some types of materials, experimental systems and methods used in many studies. Here, indicate whether each material, system or method listed is relevant to your study. If you are not sure if a list item applies to your research, read the appropriate section before selecting a response.

Materials & experimental systems

n/a	Involved in the study	
[ ]	[x]	Antibodies
[ ]	[x]	Eukaryotic cell lines
[x]	[ ]	Palaeontology and archaeology
[ ]	[x]	Animals and other organisms
[x]	[ ]	Human research participants
[x]	[ ]	Clinical data
[x]	[ ]	Dual use research of concern

Methods

n/a	Involved in the study	
[x]	[ ]	ChIP-seq
[x]	[ ]	Flow cytometry
[x]	[ ]	MRI-based neuroimaging

Antibodies

Antibodies used

BCL-XL (Cell Signaling Cat. 2762). Dilution 1:1000
MCL-1 (Cell Signaling Cat. 4572). Dilution 1:1000
BAX(Cell Signaling Cat. 2772). Dilution 1:1000
BAX (Santa Cruz Cat. sc-20067). Dilution 1:500
BCL-2 (BD. Cat. 610539). Dilution 1:1000
BAK (Millipore Cat. 06-536). Dilution 1:1000
BIM (Cell Signaling Cat. 2933S). Dilution 1:1000
Cleaved Caspase-3 (Cell Signaling Cat. 9664S). Dilution 1:500
Cleaved PARP (Cell Signaling Cat. 5625S). Dilution 1:1000
COX-IV (Cell Signaling Cat. 4850S). Dilution 1:1000
β-Actin (Sigma Cat. A1978). Dilution 1:1000
β-Tubulin (Cell Signaling Cat. 2146S). Dilution 1:1000
IRDye800CW (LICOR, 926-32211). Dilution 1:10000
IRDye800CW (LI-COR, 925-32210). Dilution 1:10000
IRDye680RD (LICOR, 926-68071). Dilution 1:20000

Validation	All antibodies used in this study were from commercial sources and have been validated in the literature and our previous publications. Such information is provided in the manufacturer's website.<br>BCL-XL, Sample PMID: 34645797<br>MCL-1, Sample PMID: 34663829<br>BAX, Sample PMID:33602934<br>BAX, Sample PMID:34389733<br>BCL-2, Sample PMID:30232009<br>BAK, Sample PMID:27488021<br>BIM, Sample PMID: 33852868<br>Cleaved Caspase-3, Sample PMID: 31453810<br>Cleaved PARP, Sample PMID:33406417<br>COX-IV, Sample PMID:34556855<br>β-Actin, Sample PMID: 33602934<br>β-Tubulin, Sample PMID:34764490<br>IRDye800CW, Sample PMID:31819006<br>IRDye800CW, Sample PMID:31819006<br>IRDye680RD, Sample PMID:31819006

Eukaryotic cell lines

Policy information about cell lines

Cell line source(s)	Head and neck cancer cell lines HN30, HN31, UMSCC6, MDA686LN, and HN5, were provided by Dr. Thomas Ow, and were originally obtained from a repository maintained by Dr. Jeffrey N. Myers, MD, PhD at the University of Texas, MD Anderson Cancer Center. All other cell lines mentioned in the figures and methods were purchased from ATCC and DSMZ.
Authentication	Cell lines were authenticated from their vendor and Einstein Genomics Core Facility. Morphology, karyotyping, and STR profiling to confirm the identity of human cell lines and to rule out both intra- and interspecies contamination.
Mycoplasma contamination	All cell lines were tested negative for mycoplasma contamination.
Commonly misidentified lines<br>(See ICLAC register)	No commonly misidentified lines used in our study.

Animals and other organisms

Policy information about studies involving animals; ARRIVE guidelines recommended for reporting animal research

Laboratory animals	For xenograft studies and pharmacodynamics analysis experiments, 6-8 weeks old Nu/Nu nude mice and NOD SCID male mice were purchased from Charles River. For toxicity studies 6-8 weeks old CD1-IGS male and female mice were purchased from Charles River. Animals were housed in a controlled environment, target conditions: temperature 18-29°C, relative humidity 30 to 70%. Temperature and relative humidity were monitored daily. An electronic time-controlled lighting system was used to provide a 12 hr light/12 hr dark cycle.
Wild animals	The study does not involve wild animals.
Field-collected samples	The study did not involve samples collected from the field.
Ethics oversight	Mice experiments were performed at Albert Einstein College of Medicine in accordance with protocols approved by the Institutional Animal Care and Use Committee of the Albert Einstein College of Medicine.

Note that full information on the approval of the study protocol must also be provided in the manuscript.
